# A T-Cell Epitope-Based Multi-Epitope Vaccine Designed Using Human HLA Specific T Cell Epitopes Induces a Near-Sterile Immunity against Experimental Visceral Leishmaniasis in Hamsters

**DOI:** 10.3390/vaccines9101058

**Published:** 2021-09-23

**Authors:** Aryandra Arya, Sunil K. Arora

**Affiliations:** Molecular Immunology, Department of Immunopathology, Post Graduate Institute of Medical Education and Research (PGIMER), Chandigarh 160012, India; aryandra.arya@gmail.com

**Keywords:** *Leishmania*, vaccine, T cell, epitopes, hamster, IFN-γ epitope, sterile immunity

## Abstract

Visceral leishmaniasis is a neglected tropical disease affecting 12 million people annually. Even in the second decade of the 21st century, it has remained without an effective vaccine for human use. In the current study, we designed three multiepitope vaccine candidates by the selection of multiple IFN-γ inducing MHC-I and MHC-II binder T-cell specific epitopes from three previously identified antigen genes of *Leishmania donovani* from our lab by an immuno-informatic approach using IFNepitope, the Immune Epitope Database (IEDB) T cell epitope identification tools, NET-MHC-1, and NET MHC-2 webservers. We tested the protective potential of these three multiepitope proteins as a vaccine in a hamster model of visceral leishmaniasis. The immunization data revealed that the vaccine candidates induced a very high level of Th1 biased protective immune response in-vivo in a hamster model of experimental visceral leishmaniasis, with one of the candidates inducing a near-sterile immunity. The vaccinated animals displayed highly activated monocyte macrophages with the capability of clearing intracellular parasites due to increased respiratory burst. Additionally, these proteins induced activation of polyfunctional T cells secreting INF-γ, TNF-α, and IL-2 in an ex-vivo stimulation of human peripheral blood mononuclear cells, further supporting the protective nature of the designed candidates.

## 1. Introduction

After 100 years of discovery, and despite extensive efforts, visceral leishmaniasis (VL) has remained without any vaccine formulation approved for human use. The development of a vaccine for kala-azar has manifested itself into an immunological challenge of its own [1]. According to World Health Organization estimates, annually 12 million new cases are being registered worldwide [2,3]. VL is a systemic infection of the reticuloendothelial system with macrophages being the primary host, if left untreated, the condition easily becomes perilous. The immunology of the disease is centered around the Th1 cytokine axis [4]. In the natural course of infection, there is a polyclonal hypergammaglobulinemia with non-specific antibody response along with the elevated levels of immunosuppressive cytokines like IL-10 and TGF-β, whereas recovering patients develop elevated levels of IFN-γ and TNF-α [5,6]. Acute infection in recovered patients results in lifelong immunity, which indicates that an effective vaccine is possible [7].

The efficacy of a T cell-based prophylactic vaccine for any infection depends upon multiple factors including primary signal generated through T cell receptor (TCR) (CD3 complex) and peptide conjugated major histocompatibility complex (MHC) molecules [8,9], while the secondary stimuli are based on coreceptor activation like CD80/86-CD28 [10]. Additionally, the generation of specific cytokine milieu affecting the outcome depends upon the pathogen-associated molecular pattern (PAMP) and Toll-like receptors (TLR) associated with antigen-presenting cells (APC) such as dendritic cells [8]. In the events leading to T cell activation, besides the role of T cell receptor (TCR) and coreceptors, the role of a core epitope molecule is not completely understood [11]. Many theories have been proposed to account for antigen discrimination for T cell selectivity and sensitivity, but the extent of the role of epitope has remained unexplored [12,13,14,15,16,17]. In this regard, many researchers have categorized T cell epitopes as dominant and subdominant or optimal and suboptimal epitopes [18,19,20,21,22]. Dominant epitopes are strong binders of MHC molecules and create a strong signal when linked in an MHC-p-TCR complex [23,24]. Optimal epitopes and strong TCR signal strength have also been linked with Th1 immune profile generation, specifically MHC-I epitopes [25,26,27,28,29]. 

In the past, many vaccine candidates have been proposed for VL, which have either utilized the whole parasite in an attenuated form or a complete antigenic protein [30]. Regarding novel vaccine candidates with a tailored response, immunoinformatic-predicted T cell-specific epitopes can be used to generate newer candidates [31,32]. Advancement in prediction models of linear and conformational epitopes has significantly boosted vaccine research in the last decades with the emergence of many epitope-based vaccine candidates for *Leishmania donovani*, *L. major*, and, *L. infantum* [33,34,35,36,37,38,39], and also for other diseases [40,41,42,43,44]. Keeping in mind the immune-correlates of protection in *L. donovani* infection, we have used the strategy of designing multiple vaccine candidates either by the addition of various T cell epitopes in a single multi-epitope candidate or with the use of multiple IFN-γ inducing epitopes to generate different multi-epitope constructs. Three previously identified and tested indigenous vaccine candidates were selected as parent antigens for epitope mapping based on their IFN-γ inducive protective nature [45,46,47]. We have used various online servers like the Immune Epitope Database (IEDB) T cell epitope identification tools for MHC-I and MHC-II, NET MHC-1, and NET MHC-2 including ‘IFNepitope’ to map T cell epitopes [48]. T cell-stimulating nature of vaccine candidates was tested on healthy human peripheral blood mononuclear cell (PBMC) for polyfunctional T cell activation along with IFN-γ, TNF-α, IL-2, and IL-10 cytokine generation, the results of which pointed toward the generation of polyfunctional T cells and strong IFN-γ and TNF-α responses. We selected the Syrian golden hamster *(Mesocricetus auratus)* as a disease model for protective efficacy evaluation in-vivo. Syrian golden hamster makes a good disease model for visceral leishmaniasis because the clinical features are similar to the humans and the animal succumb to the disease in 10–12 weeks if left untreated [49]. The protective potential and immunogenicity data in immunized hamsters revealed a very high level of protective efficacy against a virulent challenge, along with the induction of a strong Th1 biased immune response. 

## 2. Material and Methods

This study was carried out following the principles of the Basel Declaration and recommendations of the Committee for the Purpose of Control and Supervision of Experiments on Animals (CPCSEA-Guidelines). The protocol was approved by the Institute Animal Ethics Committee with endorsement No.85/IAEC/559, the Institute Bio-Safety Committee with endorsement number 265/IBC/2016, and Institute Ethics Committee INT/IEC/2021/SPL-888.

### 2.1. Epitope Identification and Multiepitope Candidate Designing

The three novel antigen genes of *L. donovani*, A2/1 (AY-377788), B4/1 (AY-161269), and F2/1 (AY-180912), were selected for epitope mapping [45]. We identified T cell epitopes using three different web servers, IEDB T cell epitope identification tools for MHC-I and MHC-II (http://tools.iedb.org/main/tcell/ accessed on 15 September 2021), NET MHC-1 (http://www.cbs.dtu.dk/services/NetMHC/ accessed on 15 September 2021) [50,51], NET MHC-2 (http://www.cbs.dtu.dk/services/NetMHCII/ accessed on 15 September 2021) [52], and IFNepitope (https://webs.iiitd.edu.in/raghava/ifnepitope/developer.php/ accessed on 15 September 2021) [48].

For IEDB web server-based MHC-I T cell epitope identification tool, the query was submitted in FASTA format for all three genes. Other parameters were set at default. For each gene, the following MHC-I alleles were selected: HLA:A01, HLA:A02, HLA:A03, HLA:A24, HLB:B07, HLB:B08, HLB:B27, HLB:B39, HLB:B44, HLB:B58, HLAC:C04, and HLAC:C06 to maximize the population coverage. Similarly, for MHC-II specific T cell epitopes, the following MHC-II alleles were selected for each gene, DP1 DP2, DQ7, DR4, DRB1, DRB3, DRB5, and main DQ. The parameters were set at default values. All the results were downloaded in .XLS format. Epitopes were selected based on IC_50_ values of <50 nM.

The NETMHC-1 4.0 input sequence was given in FASTA format, with other parameters set at the default value. HLA allele selection was as follows: HLA: A01, HLA: A02, HLA: A03, HLA: A24, HLA: A25, HLA: B07, HLA: B15, HLA: B27, HLA: B39, HLA: B40, and HLA: B58, similarly for NETMHC-2 4.0, MHC-II alleles selected were as following: DP, DQ, DR4, DRB3, and Main-DR. The results were obtained in .XLS format. The selection was based on strong binders defined as having a %rank <0.5.

For IFNepitope, the input sequences were submitted in FASTA format and parameter values were set at default. Since IFNepitope does not require HLA allele submission for epitope prediction, the predicted epitopes were checked for HLA alleles by Propred and Propred1 (MHC class 1 and class 2 binding peptide prediction servers, respectively) later on [53,54].

**Figure 1:**
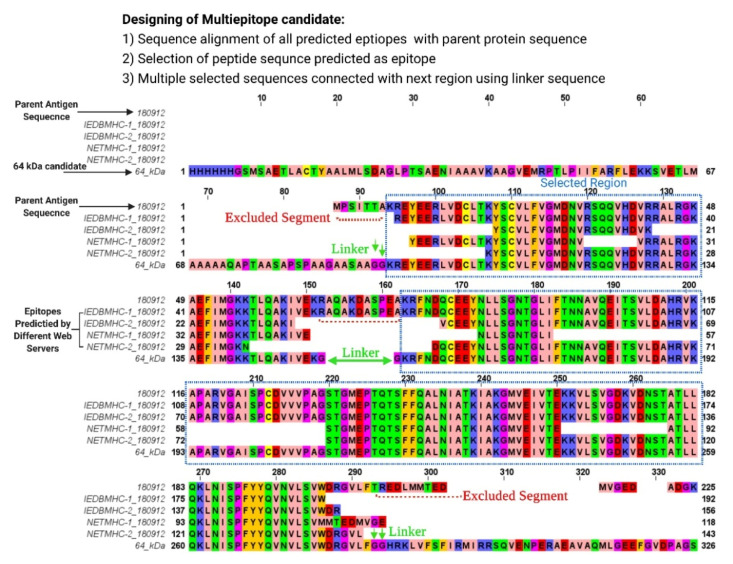
General scheme for epitope selection and designing of multiepitope construct using peptide regions identified as T cell epitopes by IEDB for MHC-I and MHC-II, NETMHC-1, and NETMHC-2. Representative image for epitope selection from F2/1 (AY-180912) for the 64 kDa construct. Sequence alignment by Clustal Omega (EMBL-EBI). Details are available in the Supplementary Materials.

Based on epitope mapping results, three different multiepitope candidate proteins were designed (Figure 1, Table 1, Figures S1–S6, and Table S1):

Construct 1: The design was based on epitopes identified through NETMHC-1 and NETMHC-2 and IEDB T-cell epitope prediction servers for both MHC-I and MHC-II. For each HLA allele, high-affinity epitopes (strong binder) with <50 nM IC_50_ were selected from the IEDB epitope server, similarly epitopes with percent rank <0.5% were selected using NETMHC-I and NETMHC-II. Integration of results from both NETMHC-1&2 and IEDB for MHC-I and MHC-II gave large sequences or island regions containing multiple overlaps of partially overlapping epitopes. These regions of multiple epitopes were linked by diglycine (-G-G-) residues to provide optimum steric flexibility and avoid any scrambling of epitope sequences by additional amino acids from the linker sequence. The sequence order of epitopes was kept similar to their native sequential order as in the parent antigen molecule (Figure S4).

Construct 2: The second construct was designed based on IFN-γ inducing MHC-I binders. All IFNepitope predicted IFN-γ epitopes were additionally screened for MHC-I affinity by Propred1. The final epitopes were IFN-γ inducing epitopes, able to bind CTL of specific HLA allele. Similar to Construct 1, the epitope-rich regions were kept in native order and linked by the diglycine linker sequence (Figure S5).

Construct 3: The third construct was designed based on the results of IFNepitope Propred, and NetChop3.0. Epitopes picked were MHC-II specific IFN-γ inducers. The flanking N-terminal and C-terminal regions of the epitope sequences were selected based on NETChop3.0 predicted cleavage sites of the proteasome complex [55,56], and these native N-terminal and C-terminal sequences were used to link epitope sequences (Figure S6).

### 2.2. Molecular Cloning and Recombinant Protein Production

For the production of multiepitope vaccine candidate proteins, the multiepitope sequences were reverse translated into their respective nucleotide sequences and obtained in pMAT cloning vectors through Gene Art (ThermoFisher, Waltham, MA, USA) (Tables S2 and S3). The construct sequences were sub-cloned into the pQE30 expression vector containing a poly-histidine (poly-HIS) site at 5′ end (Qiagen GmbH, Hilden, Germany), and transformation was carried out in M15 *Escherichia coli* for protein expression (Figures S7–S12). Protein expression was conducted at 37 °C by induction with 0.1 mM Isopropyl β- d-1-thiogalactopyranoside and the over-expressed protein was isolated from inclusion bodies by solubilizing in Tris-buffer (pH 8.0). Membrane-bound protein was repeatedly washed in a buffer containing deoxycholic acid, Tris-HCl, and ethylenediaminetetraacetic acid (EDTA). Protein was finally dissolved in 6 M guanidium HCL (Gu HCl). The excess Gu HCl was removed by overnight dialysis against double distilled water (ddH20) (Figures S13 and S14). The purified product was tested for endotoxin levels using an Endotoxin Detection Kit (THG10-0250, Hi-Media, Mumbai, India), the levels were within an acceptable range (i.e., less than 0.25 EU). The final product was lyophilized, weighed, and stored at −20 °C. The purified proteins were confirmed for their molecular weight by 12% sodium dodecyl sulfate-polyacrylamide gel electrophoresis separation and western blot analysis. Individual bands were visualized by staining with horse-radish peroxidase-conjugated anti-histidine (poly-HIS) antibody. Details are available in the Supplementary Materials.

### 2.3. Protective Efficacy Study in Hamster Model of Experimental Visceral Leishmaniasis

The protective efficacy of the multiepitope vaccine proteins was evaluated in a hamster (*Mesocricetus auratus*) model of VL. A total of 48 hamsters were divided into six groups. Different groups of animals were immunized intraperitoneally (i.p.) with 50 µg each of proteins expressed from Construct 1 (64 kDa), Construct 2 (36 kDa), and Construct 3 (29 kDa), admixed 1:1 (*v*/*v*) with Complete Freund’s adjuvant (CFA) (Sigma, St. Louis, MO, USA). The control groups of animals included an adjuvant control group, an unimmunized but infected group, and one healthy (unimmunized-uninfected) group. Freund’s adjuvant was selected to generate a sustained and slow release of antigen [57,58]. The animals in the vaccinated groups were given two booster doses of proteins i.p. at days 14 and 28 in emulsion form with Incomplete Freund’s adjuvant [59] (details are available in Table S4). Additionally, promastigotes for any in-vitro challenge were obtained from an infected hamster by culturing the crushed spleen in RPMI-1640 (Sigma, St. Louis, MO, USA) culture media supplemented with 10% fetal bovine serum (FBS) (US origin, Gibco, Waltham, MA, USA) and maintained at 25 °C.

The hamsters were challenged with 1 × 10^7^ amastigotes (*L. donovani*, Dd8) intra-cardinally on day 42 post-immunization and were maintained in Animal Biosafety Level 2 cages for 60 days post-challenge before termination of the experiment. The immunization experiments with all three multi-epitope candidate proteins were repeated twice with a fresh batch of hamsters using the same immunization protocol. 

### 2.4. Parasitological Parameters 

On the day of the termination of the experiment, the hamsters were euthanized by an overdose of anesthesia (ketamine and xylazine by Cipla, Mumbai, India), in a sterile environment and the peritoneal cavity was dissected to reveal the spleen aseptically. The spleen was weighed and bisected into two halves so that a small portion from the middle was taken for touch impressions on a glass slide, while the other half of the spleen was used for splenic culture for viable parasite detection. The touch imprints were fixed with methanol and stained with Giemsa stain for 30–40 min. The stained touch impressions were analyzed for intracellular amastigotes under an oil immersion lens in an inverted bright field microscope (Olympus BX-51, Tokyo, Japan). The data were represented as Leishman Donovan units (LDU) calculated as the number of amastigotes/number of nucleated cells × weight of the organ (mg) (Table S5).

### 2.5. Physical Parameters

Basic physical parameters of hamsters were analyzed in terms of body weight, spleen size, and spleen weight. Taken together, these parameters were used to calculate the splenic index, which is spleen weight to whole body weight ratio multiplied by 100.

### 2.6. Spleen Culture Assay

The sterile portion of the resected spleen (100 mg) from the euthanized hamsters was crushed to a single cell suspension in 2 mL of RPMI-1640 medium in a sterile tissue homogenizer. Out of 2 mL medium, 50 µL of the suspension was used to inoculate 1 mL RPMI-1640 complete medium containing 10% FBS and antibiotics (penicillin and streptomycin at a 1X concentration (i.e., 100 units of penicillin and 0.10 mg streptomycin per mL of culture media, Sigma, USA) in a sterile culture tube. The cultures were incubated at 25 °C in a Bio-Oxygen Demand incubator and an aliquot was observed every alternate day for the development of promastigotes. The promastigote population was counted using a hemocytometer (Table S6).

### 2.7. Immunological Parameters 

#### Macrophage Function Assays

The functional status of macrophages obtained from the peritoneum of experimental hamsters was assessed for intracellular parasite clearance and generation of the oxidative burst in terms of ROS production. To obtain the macrophages, the peritoneal cavity of experimental hamsters was washed with a cold RPMI-1640 culture medium before the removal of the spleen. The cells obtained from wash were seeded in a 24-well plate and left for 6 h at 37 °C with 5% CO_2_ in RPMI-1640 medium supplemented with 10% FBS and antibiotics (penicillin and streptomycin at 1X concentration, i.e., 100 units of penicillin and 0.10 mg streptomycin per mL of culture media). After 6 h, the cultures were agitated to remove the non-adherent cells and the culture media was replaced with fresh medium, leaving only adherent cells, mainly monocytes that differentiated into macrophages overnight. For the parasite clearance assay, metacyclic promastigotes were obtained by isolating amastigotes from infected hamster spleen and culturing them in blood agar slants overlaid with RPMI-1640 supplemented with 10% FBS at 25 °C temperature. After promastigote formation, the metacyclic form was obtained by culturing the promastigotes for 7–9 days without changing the culture medium (M199, 10% FBS). For evaluation of these two sets of parameters, cells were laid in individual 24-well culture plates.

- Parasite clearance: To check the ability of macrophages to clear the intracellular parasites, the adherent macrophages (50,000 cells/well) were layered with live metacyclic carboxyfluorescein succinimidyl ester (CFSE) (Sigma, St. Louis, MO, USA) stained promastigotes in a 1:10 ratio and incubated at 34 °C for 10 h. The wells were washed to remove any uninternalized promastigotes. The cells were de-adhered using Trypsin EDTA solution, at specific time intervals of 12 h, 24 h, and 48 h, washed twice in cold sheath fluid, and the acquisition was done on a flow cytometer (FACS Calibur, BD Biosciences, Franklin Lakes, NJ, USA) for CFSE signal. A quantitative assessment of internalized promastigotes was made in terms of mean fluorescence intensity (MFI). A decrease in MFI indicated parasite clearance. The data analysis was conducted using BD CellQuest™ Pro (BD Bioscience, Franklin Lakes, USA) by gating granulocytes and monocytes based on forward scatter (FSC) and side scatter (SSC) pattern. The baseline fluorescence was set based on uninfected-healthy cells.
- Oxidative burst: The capability of macrophages from the immunized hamsters for the production of reactive oxygen species (ROS) would indicate the restoration of normal function in terms of generation of oxidative burst. To assess this, 50,000 peritoneal adherent cells were cultured in a 24-well culture plate in the presence of RPMI-1640 medium supplemented with 10% FBS. At the beginning of the culture, the cells were stimulated with bacterial lipopolysaccharide (LPS, 5 µg/mL). At 12 and 24 h, the cells were stained with 2′,7′-dichlorofluorescein diacetate (DCFDA) (Sigma, St. Louis, CA, USA) for 35 min, and the acquisition was done on a flow cytometer (BD FACS Calibur, BD Biosciences, Franklin Lakes, USA) for change in MFI due to ROS mediated conversion of DCFDA to 2′,7′-dichlorofluorescein (DCF). Post-acquisition analysis was done by BD CellQuest™ Pro and the baseline was set by unstimulated-stained cells. ROS production levels were calculated in terms of ROS production index (RPI) as the ratio of the MFI test group to the MFI healthy group. The baseline fluorescence was set based on unstained-unstimulated healthy cells.

### 2.8. Post-Immunization Immune-Profiling

To assess the type and quantum of immune-response present at the end of 60 days post-immunization and infection, the immunological parameters were analyzed from the spleen tissue by evaluating the gene expression of a set of immune-response related genes (list of genes selected for study is presented in Table S7) by real-time quantitative PCR. The RNA was isolated from splenic tissue using Trizol (Sigma, St. Louis, MO, USA) and cDNA was prepared using a first-strand synthesis kit (iScript cDNA Synthesis Kits, Bio-Rad, Hercules, CA, USA). Primers for selected genes of interest were designed using Primer-BLAST (NCBI). 

The RT qPCR was carried out on the LC480 platform (Roche Diagnostics Nederland BV). The reaction parameters were set for an annealing temperature of 59 °C for 60 s with a consecutive 45-s amplification step at 72 °C. The amplification reaction was programmed for 37 cycles. The data were obtained in the form of cycle threshold (Ct) values. The expression levels were normalized to the reference gene (γ-Actin). The 2^−ΔΔCT^ method was employed to calculate the relative expression of each gene. The ΔCT values were calculated using the formula: ∆Ct = Ct (Gene of interest)—Ct (Reference gene), and then, ∆∆Ct was calculated using the equation: ∆∆Ct = ∆Ct (treated sample i.e., immunized or unimmunized animals)—∆Ct (untreated sample, i.e., healthy uninfected animals), and final values were given in terms of fold change (2^−ΔΔCT^) to the healthy control.

### 2.9. Polyfunctional T Cell Activation

Polyfunctional T cells are defined by their secretion of multiple cytokines at the same time. IFN-γ, TNF-α, and simultaneously IL-2 secreting T cells are directly correlated to the protective response. To assess the capability of multiepitope protein constructs in inducing a polyfunctional T-cell response, the peripheral blood mononuclear cells (PBMC) from healthy human volunteers were stimulated in-vitro with the 64 kDa, 36 kDa, and 29 kDa proteins at 20 µg/mL concentration. Phorbol 12-myristate 13-acetate (PMA at 100 ng/mL) (Sigma Aldrich, St. Louis, MO, USA) along with 50 ng/mL ionomycin (Sigma Aldrich, St. Louis, MO, USA) and phytohemagglutinin (PHA at 10 µg/mL) (Sigma Aldrich, St. Louis, MO, USA) were used as control mitogen activators in separate wells. Total *Leishmania* promastigote soluble antigen (LPSA) at a 20 µg/mL concentration was used as the antigen control. At 14 h post-stimulation, 5 µg/mL Brefeldin A (Sigma Aldrich, St. Louis, MO, USA) was added, and the cells were harvested after a further 2 h incubation, washed with sterile *phosphate buffered saline*, and fixed with Cytoperm Cytofix buffer (BD Biosciences, Franklin Lakes, NJ, USA). The cells were then stained with PE Mouse Anti-Human CD3 (552127, BD Biosciences, Franklin Lakes, USA) for surface staining and with PE-Cy™7 Mouse Anti-Human IFN-γ (557643, BD Biosciences, Franklin Lakes, USA), BV421 Mouse Anti-Human TNF (562783, BD Biosciences, Franklin Lakes, NJ, USA), FITC Mouse Anti-Human IL-2 (340448, BD Biosciences Franklin Lakes, NJ, USA), and Allophycocyanin (APC) Rat Anti-Human IL-10 (554707, BD Biosciences, Franklin Lakes, USA) for intracellular cytokine staining. Finally, the acquisition was undertaken on a flow cytometer (FACS CANTO, BD Biosciences, Franklin Lakes, NJ, USA). The lymphocytes were gated based on the forward scatter (FSC) and side scatter (SSC) pattern. The gating and baseline settings were based on unstimulated-stained controls. The frequency of triple-positive (INF-γ^+^, TNF-α^+^, and IL-2^+^) and dual positive (INF-γ^+^, and TNF-α^+^) cells was calculated based on Boolean gating for INF-γ^+^, TNFα^+^, and IL-2^+^ triple-positive CD3^+^ T-cells, using FlowJo version 10.1 (Figures S15–S18).

### 2.10. Statistical Analysis

A two-way comparison between the test and control group was performed using the Student’s *t*-test. Multiple comparisons amongst different groups were performed by analysis of variance (ANOVA). The statistical analyses were made using GraphPad Prism 6.0 software and were considered significant at the level of *p* < 0.05.

## 3. Results

### 3.1. Multiepitope Vaccine Designing

Three multiepitope constructs were designed utilizing the results obtained from the prediction tools. The first construct of molecular mass 64 kDa was based on the dominant epitopes of the high affinity for both MHC-I and MHC-II alleles. It included multiple overlaying epitopes from the regions aa 1–83 from B4/1 (AY161269), aa 8–65, aa 77–206 from F2/1 (AY180912), and aa 12–103, aa 125–223, aa 242–310, and aa 264–403 from A2/1 (AY 377788). The second construct of 36 kDa mass was based on MHC-I (CTL) specific to the IFN-γ inducing epitope as predicted by the IFNepitope and Propred1 results. A total number of 63 epitopes were used out of which 17 were dual binders for the MHC-I and MHC-II alleles. The third construct of 29 kDa was designed using 24 epitopes positive for MHC-II alleles out of which seven were also MHC-I (CTL) binders (Table 1).

### 3.2. Multi-Epitope Construct Proteins Induced Significant Protection in the Hamster Model of Experimental Visceral Leishmaniasis

Survival efficacy: post-challenge, all the animals in the three immunized groups survived, while two animals in the un-immunized infection-alone group and one animal from the CFA-only group died due to disease-induced morbidity. Postmortem analysis revealed an enlarged spleen with a high parasite burden.

### 3.3. Parasitological Parameters and Parasite Load

**Figure 2:**
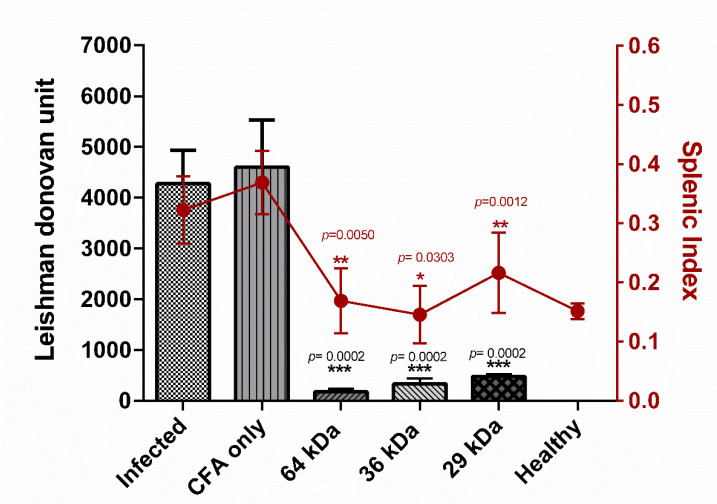
Mean parasite load and splenic index from the spleen of different animal groups. LDU = Leishman Donovan unit. (number of amastigotes/number of nucleated cells × weight of the organ(mg)). Splenic index, the ratio of (spleen weight/whole body weight) × 100. *n* = 8. Mean ± SEM. Statistical comparison between Infected and test group. Student’s *t*-test. Level of significance, ns *p* > 0.05, * *p* ≤ 0.05, ** *p* ≤ 0.01, *** *p* ≤ 0.001.

The animals from all the immunized groups resisted an increase in parasite burden as observed by a significantly lower parasite number (Leishman Donovan unit: LDU) in spleen tissue touch imprints (Figure S19A,B): 178 (±47.03 SEM, *p* = 0.0002) in the 64 kDa immunized group, 332 (±105.32 SEM, *p* = 0.0002) in the 36 kDa immunized group, and 478 (±50.10 SEM, *p* = 0.0002) in the 29 kDa immunized group, compared to 4277 (±661.1 SEM) and 4602 (±931.02 SEM) in the unimmunized infected group and adjuvant control group, respectively (Figure 2). The splenic index of immunized animals also corresponded with the parasite burden (Table S5).

### 3.4. Splenic Culture, Parasite Growth

**Figure 3:**
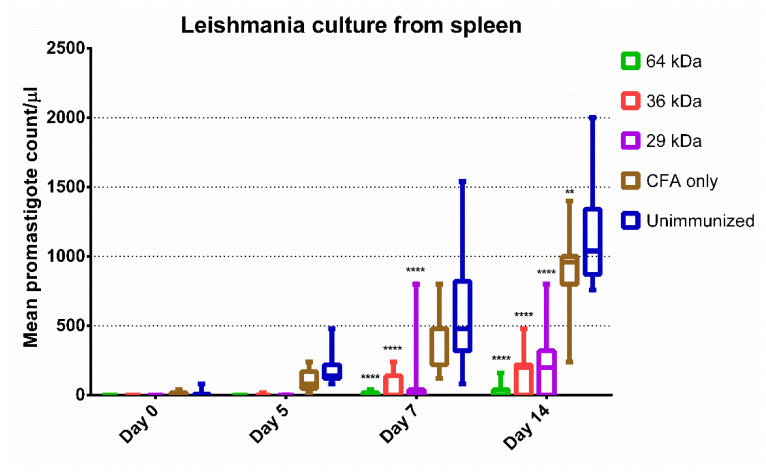
Mean promastigote growth in different groups at day 0, day 5, day 7, and day 14. *n* = 8. Box and whiskers for median value, lower, and upper quartile. * Difference statistically significant as determined by ANOVA. Level of significance, ns *p* > 0.05, ** *p* ≤ 0.01, **** *p*≤ 0.00001.

To account for any residual viable parasites in the spleen of immunized animals, we conducted the spleen tissue culture. The spleen cultures from unimmunized animals showed motile promastigotes as early as day 5 post-inoculation in concordance with a high parasitic load. (Figure 3). The promastigotes became visible in cultures from animals immunized with the 36 kDa and 29 kDa proteins by day 14, indicating a significantly lower number of viable parasites in these animals compared to the unimmunized groups. However, no promastigotes could be detected in the majority of samples from 64 kDa immunized group animal spleen cultures throughout the 14-day culture, suggesting the absence of viable parasites or an extremely low number of viable parasite burden in the spleen (Table S6).

### 3.5. The Immunization with Multiepitope Antigens Induced Normalization of Macrophage Functions for Effective Clearance of Intracellular Parasites

To further evaluate the mechanism of protection in immunized animals, we checked the functional status of peritoneal macrophages from these animals in terms of their capability to clear the intracellular parasites and produce reactive oxygen species (ROS) as a signature of normalized oxidative burst. 

### 3.6. Parasite Clearance Assay

**Figure 4:**
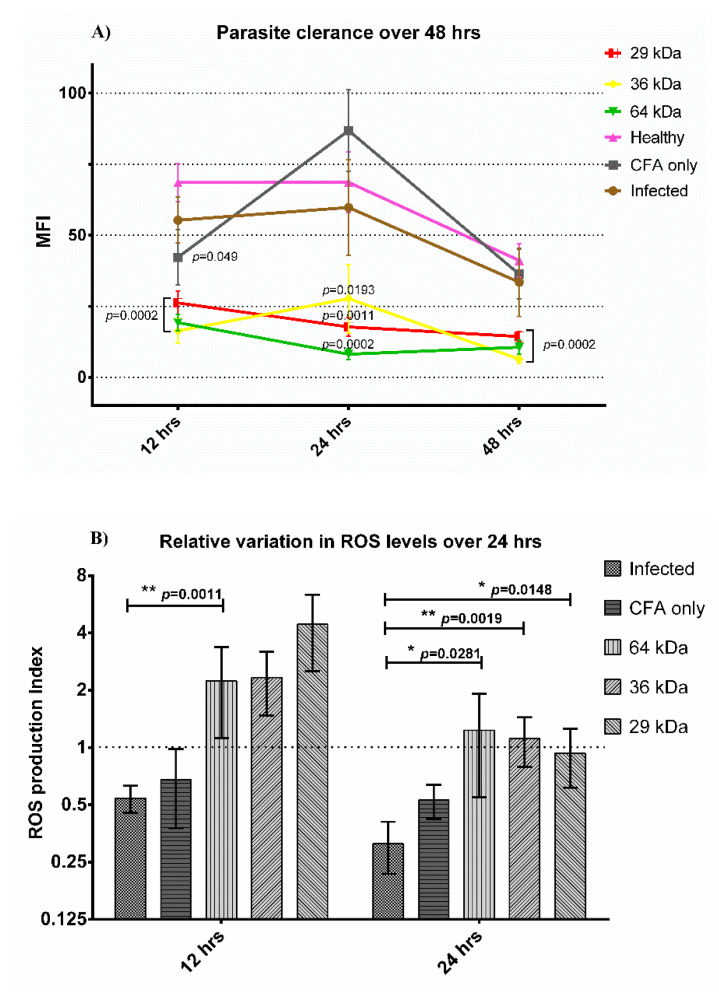
(**A**) Parasite clearance observed at different time intervals. Statistical comparison between healthy and test groups. Average MFI (mean fluorescent intensity) at different time points from adherent peritoneal cells of different animal groups. (**B**) Variation in ROS production levels over 24 h. ROS is expressed relative to healthy control, ROS production index (RPI) = MFI test group/MFI healthy group. The dashed line indicates the expression levels of healthy cells. CFA: Freund’s adjuvant. *n* = 8, mean values ± SEM. (**A**) Statistical comparison of Student’s *t*-test between the test group and healthy group. Level of significance, ns *p* > 0.05, * *p* ≤ 0.05, ** *p* ≤ 0.01.

The CFSE-stained parasite clearance assay showed a significant difference in the level of infection at 12 h, 24 h, and 48 h post-infection in-vitro compared to cells from unimmunized-infected hamsters (Figure 4A, Figure S20A–D, and Table S8). At 12 h, the MFI from cells of all three immunized groups was significantly lower compared to the MFI values from uninfected or unimmunized-infected animals, indicating facilitated parasite clearance by cells from immunized animals. The parasite clearance was also significant at 24 h and 48 h, indicating the normalized functional status of macrophages to clear the intracellular parasites after immunization. 

The ability of macrophages to clear the intracellular parasites depends upon their ability to produce ROS. The *Leishmania*-infected macrophages during active disease are shown to have a stunted ability to produce ROS due to *Leishmania* mediated down-modulation of the oxidative burst. Therefore, the restoration of macrophage functions can be determined by their capability to induce ROS upon stimulation. We assessed the functional state of macrophages from immunized animals for the production of ROS, as determined by the DCFDA assay. For estimation of ROS, we estimated the level of fluorescence emitted due to 2′7-dichlorofluorescein (DCF), which is directly proportional to the production of ROS (Figure S21A,B), interpreted in the terms of the relative level of ROS production in cells from the immunized compared to unimmunized animals, termed as the ‘ROS production index (RPI)’. The difference in ROS production in cells from immunized animals was significantly higher compared to unimmunized-infected samples from 12 h onward for the 64 kDa (2.24 ± 1.12 SEM, *p* = 0.0011), while this difference became statistically significant for all three proteins at 24 h with an RPI of 1.23 (±0.68 SEM *p* = 0.0019) for the 64 kDa group; 1.11 (±0.32 SEM *p* = 0.0002) for 36 kDa and 0.93 (±0.31 SEM, *p* = 0.0002) for 29 kDa (Figure 4B, Table S8). A value of RPI close to 1 was equivalent to ROS produced by naïve cells, and an indicator of the normalized functional status of macrophages from immunized groups. These findings were in perfect correlation with the parasite clearance assay, where higher parasite clearance levels from macrophages of immunized animals were achieved, indicating the enhanced leishmanicidal activity due to enhanced oxidative burst against *Leishmania donovani* in immunized animals.

### 3.7. Immunization with Multiepitope Protein (64 kDa) Induces a Strong Th1 Polarized Response in the Spleen of Immunized Animals

**Figure 5:**
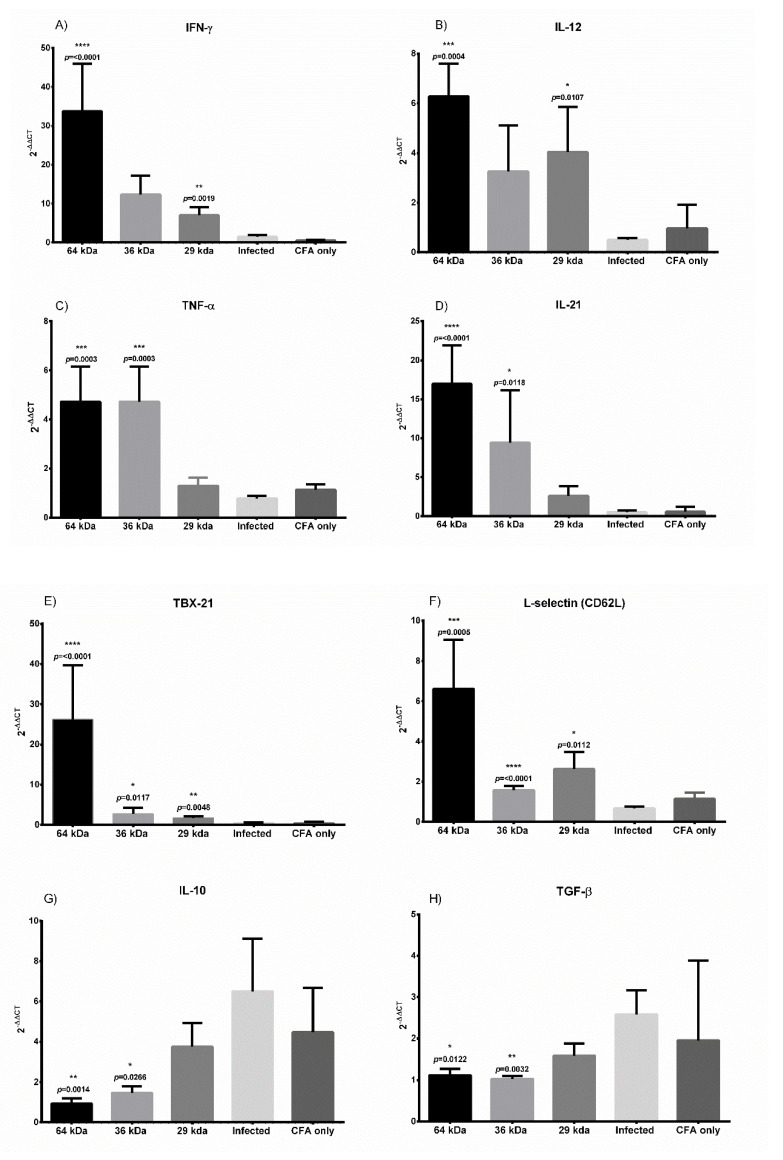
Relative fold change in gene expression levels of (**A**) IFN-γ, (**B**) IL-12, (**C**) TNF-α, (**D**) IL-21, (**E**) TBX21, (**F**) L selectin, (**G**) IL-10, and (**H**) TGF-β from immunized and unimmunized animal’s spleen. *n* = 8 for 64 kDa and 36 kDa, *n* = 7 for 29 kDa and *n* = 5 for the infected. Mean values ± SEM. Statistical comparison between infected and test group, Student’s *t*-test. Level of significance, ns *p* > 0.05, * *p* ≤ 0.05, ** *p* ≤ 0.01, *** *p* ≤ 0.001, **** *p* ≤ 0.00001.

To further understand the nature of protective responses generated in the spleen of immunized animals, we conducted a quantitative real-time PCR analysis for Th1 cytokines and genes related to T-cell activation. The results for real-time PCR were interpreted in terms of fold change to unimmunized healthy animals (Figure 5). Real-time PCR-based immuno-profiling revealed a strong Th1-biased immune profile of the spleen for all three immunized groups at the end of 60 days (post-infection), indicating a significant upregulation of IFN-γ (33-fold in the case of the 64 kDa immunized animals) compared to the unimmunized controls (Figure 5A). A similar trend was observed for the 36 kDa immunized group (11.6-fold) and, for 29 kDa the increase was 6.9-fold. The expression of signature Th1 cytokines like IFN-γ, IL-12, and TNF-α was found to be significantly upregulated in immunized animals with strikingly better results with the 64 kDa group (Figure 5B,C), which is in agreement with macrophage activation and ROS production [4,60,61]. IL-21, which is an activator of cytotoxic T-lymphocytes (CTL) and has a significant role in the clearance of intracellular infection, was also found to be significantly upregulated in the 64 kDa and 36 kDa at 16- and 9.8-fold, respectively (Figure 5D). IL-21 is produced by T helper cells and affects NK cells along with CD8^+^ T cells. Additionally, IL-21 has also been reported to revert CTL exhaustion and promote antigen-specific T cell activation [62,63,64,65]. Furthermore, immune-profiling suggested a high level of inflammation and recruitment of activated T cells in the case of the 64 kDa group based on elevated levels of chemokine (C-X-C motif) ligand 9 (CXCL9), the chemokine receptor CXCR3 and C-C chemokine receptor type 7 (CCR7). The levels of CXCL-9 were 41 times higher in the 64 kDa immunized group and 6-fold higher in the case of 36 kDa, although the levels for 29 kDa remained at only 3.5-fold higher, but were better compared to the infection alone group (0.8-fold change). Statistically, the data were significant only for the 64 kDa protein. The interleukin 23 receptor (IL23R), which is an indicator of the inflammatory profile, was expressed 11-fold higher in 64 kDa immunized animals, but only 1.6-fold and 2.6-fold higher for the 36 kDa group and the 29 kDa group, respectively, not being statistically significant. The level of CCR7, a lymphoid homing receptor, was at a 12-fold increase for the 64 kDa immunized group, 2.7-fold higher for the 29 kDa immunized group, and 1.3-fold higher for the 36 kDa immunized animal group (Figure S22). The expression levels of (T-box transcription factor) TBX21 for the 64 kDa were 26-fold higher along with 2.8-fold for 36 kDa and 1.7-fold for the 29 kDa groups, respectively, all of which were significantly higher than the unimmunized group, further indicating the development of Th1 biased immune-response with immunization (Figure 5E). The expression level of L-selectin, which is a secondary lymphoid tissue homing receptor, an indicator of memory T cell response, was significantly upregulated at 6.6, 1.6, and 2.6-fold change in the 64 kDa, 36 kDa, and 29 kDa immunized animals compared to infected unimmunized animal groups, indicating T cell activation and formation of memory component (Figure 5F). In contrast to the Th1 profile, the markers for the Th2 cytokine profile showed a low level of expression in the case of immunized animals with all three antigen constructs. Most crucially, the levels of IL-10 and TGF-β remained significantly low for all immunized groups except 29 kDa (at a 3.7-fold increase) (Figure 5G,H), further indicating the predominance of a Th1 polarized response in immunized animals compared to the unimmunized-infected group. 

Furthermore, the levels of programmed death-ligand 1(PD-L1: CD-274), a major immunosuppressor was significantly lower in all the immunized groups compared to unimmunized infected animals (Figure S22).

### 3.8. The Multiepitope Proteins Induced Activation of Human Polyfunctional T-Cells Ex-Vivo

**Figure 6:**
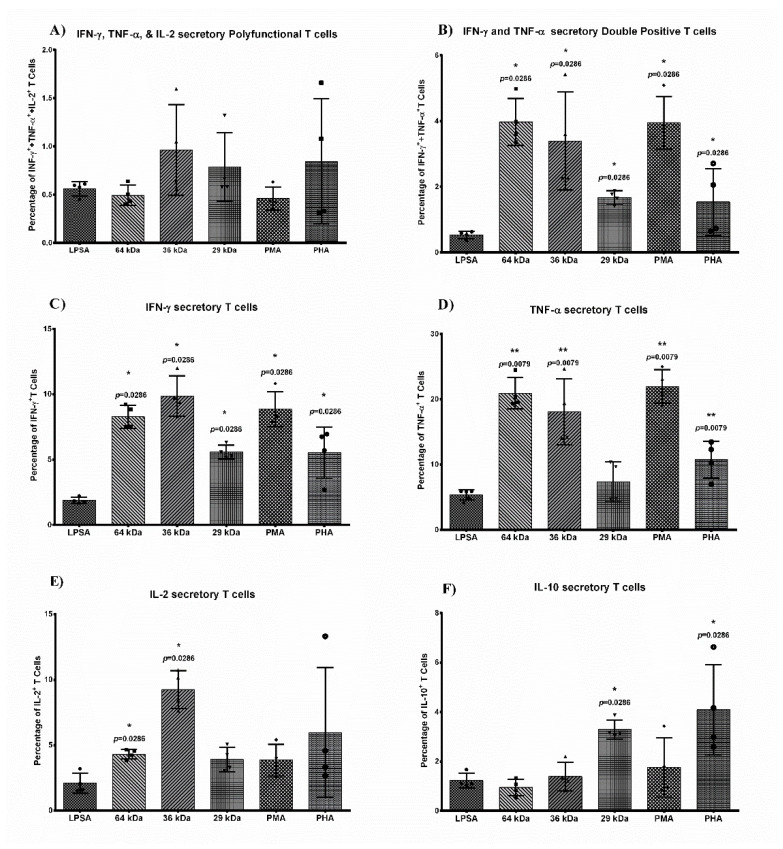
Bar graph comparing the frequency of CD3+ T cells secreting IFN-γ, TNF-α, IL-2, and IL-10. (**A**) Frequency of polyfunctional CD3+ T cells expressing IFN-γ, TNF-α, and IL-2 simultaneously. (**B**) Frequency of dual positive CD3+ T cells expressing IFN-γ and TNF-α simultaneously. (**C**) Frequency of CD3+ T cells expressing IFN-γ. (**D**) Frequency of CD3+ T cells expressing TNF-α. (**E**) Frequency of CD3+ T cells expressing IL-2. (**F**) Frequency of cells expressing IL-10. *n* = 4. Symbols for individual values, • LPSA, ■ 64 kDa, ▲ 36 kDa, ▼ 29 kDa, ♦ PMA & ● PHA. Mean values and SD. Statistical comparison between LPSA and test group. Student’s *t*-test. Level of significance, ns *p* > 0.05, * *p* ≤ 0.05, ** *p* ≤ 0.01.

To evaluate the T-cell activation potential of the vaccine proteins, we checked the ex-vivo stimulation of polyfunctional T-cells in healthy human PBMC with these proteins. Based on the cytokine profile of individual T cells as calculated using Boolean Gating for INF-γ, TNF-α, and IL-2, and IL-10 secretion, we found the 36 kDa construct to be the highest inducer of triple-positive T cells (cells making IFN-γ, TNF-α, and IL-2 simultaneously) at 0.96% (mean ± 0.47 SD), which was higher than LPSA induced T cells at 0.55% (mean ± 0.073 SD), respectively (Table S9). The 29 kDa and 64 kDa antigens were also able to induce the cells at a comparable frequency of 0.78% (mean ± 0.35 SD) and 0.49% (mean ± 0.10 SD), respectively (Figure 6A). These values were higher than PMA-ionomycin induced T cells and comparable to PHA-induced T cells at 0.46 (mean ± 0.11 SD) and 0.84% (mean ± 0.64 SD), respectively. Besides triple-positive cells, the frequency of dual positive (INF-γ and TNF-α) T cells were also significantly higher for all three proteins when compared to LPSA. The highest frequency of dual positive cells, for IFN-γ and TNF-α, was present for the 64 kDa protein at an average 3.97% (mean ± 0.71 SD, *p* = 0.0286) compared to the LPSA induced population of 0.53% (mean ± 0.11 SD) T cells. Similarly, a significantly higher number was observed for the 36 kDa protein at an average frequency of 3.39% (mean ± 1.4 SD, *p* = 0.0286) cells and 1.67% (mean ± 0.20 SD, *p* = 0.0286) cells for 29 kDa (Figure 6B). 

The frequency of T cells producing IFN-γ alone was found to be very high post-stimulation with the 36 kDa and 64 kDa at an average of 10% (mean ± 1.54 SD, *p* = 0.0286) and 8.27% (mean ± 0.8 SD, *p* = 0.0286) of total T cells, respectively, while the 29 kDa antigen-induced 5.5% cells (mean ± 0.52 SD, *p* = 0.0286) (Figure 6C). This was further supported by the high level of IFN-γ in the culture supernatant as checked by ELISA (Figure S23). These data support our premise of selecting T cell-specific epitopes using the IFNepitope tool in the case of the 36 kDa and 29 kDa being strong IFN-γ inducers. In addition to IFN-γ, we detected a high frequency of T cells also producing TNF-α, which is a protective cytokine and supports the IFN-γ based Th1 cytokine axis through IL-12 and enhances IFN-γ production [66,67]. The 64 kDa antigen was able to induce an average 21% (mean ± 2.4 SD, *p* = 0.0079) of T cells for TNF-α production, while 36 kDa induced 18% (mean ± 5.0 SD, *p* = 0.0079) and 29 kDa induced only 7.35% (mean ± 3.06 SD) of cells (Figure 6D). Furthermore, IL-2 production was also found to be significant for the 36 kDa at a mean frequency of 9.2% (mean ±1.4 SD, *p* = 0.0286) T cells, which was 4.3% for 64 kDa (mean ± 0.37 SD, *p* = 0.0286) and 3.9% for 29 kDa (mean ± 0.93 SD) (Figure 6E). It was interesting to note that the frequency of IL-10 producing T-cells in the same experiment was very low at only 0.9% (±0.33 SD) for 64 kDa and 1.39% (±0.57 SD) for the 36 kDa, which was comparable to the number of cells induced by LPSA [1.2% (±0.29 SD)] (Figure 6F). The low levels of IL-10 correlated with higher levels of protection, as in the case of both 64 kDa and 36 kDa minimal IL-10 presence was detected in both human PBMC stimulation experiment as well as in the spleens of both 64 kDa and 36 kDa immunized animals.

## 4. Discussion

The previous generation of vaccines for VL has mainly relied on the generation of enhanced IFN-γ responses, with few attempts at a possible suppression of IL-10 [30]. So far, vaccine designs have been based on either whole attenuated parasite form or multiple antigens and due to partial efficacy, none is licensed for human use yet [68]. These whole antigen-based vaccines could carry a mix of high affinity and low-affinity random epitopes. In this regard, the selection of strong binders (high-affinity epitopes) for vaccine design has been shown to induce inflammatory responses and the generation of memory T cells [69,70,71,72,73,74]. An epitope has a quantitative and qualitative role in immune-response generation, specifically TCR-dependent memory cell formation [14]. The role of a single epitope in TCR activation and its downstream effect is a matter of debate and research [9] because the substitution of a single amino acid in the epitope sequence can alter the immune response to a great extent [75,76]. The prevailing qualitative model of memory formation depends on the cytokine environment generated by APC and other supportive cells [14,77]. The quantitative model, however, relies on TCR and the immune-signalosome complex involving peptide–MHC–TCR interaction strength, amount of antigen, and interaction of costimulatory molecules [28,78,79,80]. In vaccine design, the level of control on the quantitative model is debatable and cannot be controlled directly. In the context of the qualitative model, the modern immunoinformatic approach can be utilized to dictate the choice of the epitope, which can affect the TCR–peptide–MHC signalosome response by utilization of either high or low-affinity epitopes [81,82]. The approach of MHC-I and MHC-II specific epitope identification has been utilized by researchers in the last five years for Leishmaniasis vaccine research [83]. However, most of these vaccine ideas are limited to *in-silico* docking models. Additionally, the number of epitopes included in a majority of research has remained limited to a selective few epitopes, limiting the overall size of vaccine candidates [83,84,85,86]. Since a larger size antigen is known to generate a higher degree of immunogenicity, this is a limitation in the case of vaccine design because smaller size antigens are generally poor immunogens [87]. On the other hand, the variants with a large number of epitopes such as in chimeric molecules have shown promising results [88].

Previous studies from our lab have identified three antigenic genes of *L. donovani* from an indigenously made cDNA library [45]. When repeatedly tested as potential vaccines in the hamster and mice model, the responses indicated strong IFN-γ induction and an optimal level (70–80%) of protection. Although a significant reduction in splenic parasite burden was observed, sterile immunity could not be achieved, probably due to co-induction of IL-10 in the immunized animals [46,47,89]. Taking our previous research work as the background, we modified our approach from a whole antigen-based subunit vaccine to a T cell epitope-based vaccine. In the present proof-of-concept study, we have tested the possibility of developing vaccine candidates based on the selection of MHC-I and MHC-II specific multiple T cell epitopes, along with INF-γ specific T cell epitopes, all from the three previously identified cDNA clones of *L. donovani* [45]. Based on the immunoinformatic mapping, the data produced a large number of MHC-I and MHC-II binding epitopes with the majority of epitopes being overlapping and present in a cluster of epitope-rich islands. Similar results were obtained with the usage of the IFNepitope tool. The presence of T-cell epitopes in clusters could be of some evolutionary significance or just a chance occurrence with our three antigen genes, therefore, this needs to be studied and resolved further. We could not come up with any previous study in the literature to account for the clustering of T cell epitopes. In our opinion, the clustering of immunodominant epitopes could occur due to evolutionary selective pressure in which certain regions of antigen escape the MHC affinity due to reasons not known yet. One study does point toward common amino acid polymorphisms and epitope binding repertoires in HLA-DRB1 associated with lower Leishmaniasis susceptibility [90]. Our study further raises the question regarding epitope representation on the MHC molecule, since the epitope identification is based on human HLA, despite this fact, our study showed their ability to raise a T cell-based immunity in a hamster model. This points toward the conserved nature of high-affinity epitopes across species. The hypothesis of specific epitopes, both conformational and linear, being conserved through evolution and shared in antigens from different species, have been validated by different publications [91,92,93,94]. 

The three designed multi-epitope constructs in this study, when tested as vaccines in the hamster model of experimental VL along with CFA, induced a significant protective response to a virulent challenge along with a strong Th1 biased immune response. Our observations for the designed vaccine candidates, especially the 64 kDa and 36 kDa, indicated a robust immune response, as evident from a significant protection level, even achieving a near-sterile immunity in the case of the 64 kDa protein and a notable reduction in IL-10 cytokine production also correlated with increased vaccine efficacy [46,47]. Scant parasites seen in splenic touch imprints of 64 kDa immunized animals could most likely be the remnants of dead parasites, as no motile promastigotes were observed in most of the splenic culture assay. The splenic culture assay is considered as the gold-standard method of detecting the viable parasites in the spleen as only living and viable parasites would be able to differentiate to promastigote form [95,96]. Active *Leishmania* infection would normally lead to hepato-splenomegaly with an increase in spleen size and weight, which could be observed in the infected but unimmunized animals. However, the parasite load as well as the splenic index, which is a proxy indicator of spleen weight, was consistently low in animals immunized with all three multiepitope proteins designed in the current study, which primarily indicated the protective efficacy of these vaccine candidates in terms of restricting the parasite growth. The data support the utilization of parental antigens as an ideal scaffold for the selection of epitopes using bioinformatics tools, which has enabled the generation of a vaccine approach that is superior to other approaches reported earlier using the individual parental antigens.

The *Leishmania* parasite completely hijacks the killing machinery of host cells viz. macrophages. A predominance of immuno-suppressive macrophages with a subdued capacity to generate optimum oxidative burst, which is necessary for the clearance of parasites, is seen in active *Leishmania* disease [97]. Thus, the protective response of any vaccine would envisage the reversal of this process and conversion of immuno-suppressive to activated macrophages having enhanced capacity to generate enough ROS to clear the intracellular parasites. The peritoneal macrophages from the immunized animals displayed the characteristics of normalized functions with enhanced capability to clear a second parasite challenge ex-vivo very effectively, along with the generation of significantly higher amounts of ROS. One possible explanation for this observation could be underlying hyperresponsive adaptation in myeloid cells. Hyper responsive adaptation in macrophages is an increased expression of inflammatory genes after re-stimulation of macrophages. This depends upon increased expression of signaling molecules, metabolic reprogramming, and the epigenetic change at *cis*-regulatory elements. Perseverance of these features, even after elimination of stimulus (i.e., removal of initial antigen can facilitate or potentiate secondary activation [98,99]). Additional experiments will be required to understand the nature of these responses [99,100,101]. Although the details of the exact mechanism for this state in our study remains to be explored, the functioning of the iNOS pathway in immunized animals seems evident for their ability to clear in vitro reinfection such as the ROS pathway, which in turn, is connected to inflammasome activity in macrophages [102,103]. This is in agreement with previous studies where ROS generation has been shown in support of *L. donovani* clearance [104,105]. The protective response in our study is supported by a high level of inflammation and T cell activation in hamsters immunized with the designed multiepitope proteins. The data indicate an upregulated expression of IFN-γ, IL-12, and TNF-α, which forms the signature for the Th1 type of immune response being induced in the immunized animals, necessary for the protective response as seen in these animals. This type of response was most evident in animals immunized with the 64 kDa protein, which induced a near sterile immunity. Development of memory T cell responses was supported by elevated expression of genes like TBX21, IL-21, IL-23r, IL-12, and L-selectin in the spleen tissue of all the immunized animals, especially with the 64 kDa protein (Figure S22). Among these, the IL-21 has been shown to mediate inflammatory axis in parasite-infected organs; mice deficient in IL-21 are unable to clear parasite in a TBX21, STAT-4, and IL-12 dependent manner [106,107]. IL23r is present on T cells and NK cells and has been shown to have a protective role in VL infection via IL-17 mediated pathways [108]. Further T cell functioning and development of proliferative T cells was evident from the high levels of CXCL9 and CXCR3 in the case of the 64 kDa immunized group, further indicative of Th1 polarization (Figure S22). The CXCL9 increases transcription of T-bet and RORγ, leading to the polarization of Foxp3 type 1 regulatory (Tr1) cells or T helper 17 (Th17) cells from naive T cells via STAT1, STAT4, and STAT5 phosphorylation [109]. The Treg component was low in immunized animals in our study as indicated by significantly downregulated expression of IL-10 and TGF-β. This type of response is important for any vaccine to be efficacious against this parasite, since the pathogenesis of *Leishmania* infection is marked by the presence of Treg cells, which cause a broad level of immune suppression. 

A further elaboration of protective responses in the human context was shown by the activation of polyfunctional T cells. Polyfunctional T cells have been correlated with protection in multiple studies [110,111]. It was interesting to find that the designed protein constructs were able to induce the polyfunctional T-cells in the PBMC from healthy human donors to produce IFN-γ, TNF-α, and IL-2 simultaneously. Similarly, cytokine levels and frequency of IFN-γ producing T cells were significantly high for all the proteins asserting the role of dominant and IFN-γ specific epitopes as the inducer of Th1 responses, justifying our epitope-selection process (Figure S24). The induction values were equivalent and higher in some cases when compared to other candidates tested in other studies [112,113,114]. These data further emphasize the protective potential of our antigen constructs via the induction of polyfunctional T cells.

In summary, among the three designed multi-epitope constructs, the 64 kDa candidate contained the largest number of dominant epitopes, which were both strong binders of MHC-I and MHC-II, along with regions of IFN-γ specific epitopes, and this protein induced a near-sterile immunity in hamsters when used as a vaccine. The protective nature of the 64 kDa protein was also evident by the generation of polyfunctional T cells and a very high frequency of IFN-γ and TNF-α producing dual-positive T-cells on activation of human PBMC with this protein. High-affinity dominant epitopes are generally considered immune-responsive with the potential to activate cytotoxic T lymphocytes (CTLs) and a skewness toward the Th1 cytokine profile [25,26]. The 36 kDa protein, designed with IFN-γ specific epitopes with a preference for epitopes that had an affinity toward MHC-I, but also contained some dual-specificity epitopes for both MHC-I and MHC-II, was also able to reduce parasite burden significantly with inflammatory response and absence of any immune-suppressive responses, however, a sterile immunity could not be achieved with this protein. The results from human PBMC stimulation indicated polyfunctional T cell activation along with IFN-γ and TNF-α production capability. The levels of IFN-γ in suboptimal concentrations have been shown to have a vital role in the optimization of innate immune cell functions including phagocytosis and destruction of reminiscent pathogens [115]. Previously, other researchers have also shown a limited role of CTL responses in the clearance of parasites [116]. The 29 kDa protein was designed from IFN-γ inducing epitopes with affinity to MHC-II, and also contained some dual-specificity epitopes for both MHC-I and MHC-II. Immunization with the 29 kDa protein induced a lower level of protection, although it showed increased IFN-γ production, but had a very insignificant effect on IL-10 producing T cells. The 29 kDa protein contained only 24 core epitopes compared to 63 epitopes in 36 kDa and 1317 epitopes in 64 kDa proteins in various overlapping configurations, which seem to have limited its protective efficacy. 

## 5. Conclusions

VL has remained without a protective vaccine probably due to the lack of a candidate that could generate a predominant Th1 polarized response while keeping the immuno-suppressive responses to a minimum. We have shown the possibility of developing a vaccine candidate, the 64 kDa protein, with tailored Th1 responses through the selection of T cell-specific high-affinity and IFN-γ inducing epitopes, which could induce a possible sterile immunity against a lethal challenge. Our other two proposed candidates were also able to induce a significant level of protection in the hamster model but with limited efficacy. The exact mechanism of these responses, along with the kinetics of T cell epitopes, remains to be further evaluated. Further confirmation of complete protection and absence of parasite after immunization with the said protein will be needed in the same or a different animal model. This approach opens up the possibility of developing tailored immune response-specific vaccines against other infectious agents such as HIV, malaria, coronaviruses, and even cancer.

## Figures and Tables

**Table 1:** Multiple overlaying regions of epitopes used in designing the multiepitope constructs.

**64 kDa**	**B4/1 (AY 161269)**	**F2/1 (AY 180912)**	**A2/1 (AY 377788)**
1317 epitopes	aa 1–83	aa 8–65	aa 12–103
		aa 77–206	aa 125–223
			aa 242–310
			aa 364–403
**36 kDa**	**B4/1 (AY 161269)**	**F2/1 (AY 180912)**	**A2/1 (AY 377788)**
63 epitopes	aa 23–52	aa 41–58	aa 14–28
	aa 69–89	aa 186–196	aa 46–70
	aa 99–109		aa 81–94
			aa 98-119
			aa 139–178
			aa 185–225
			aa 242–283
			aa 372–384
**29 kDa: Core multiepitope sequence and flanking regions included in 29 kDa construct as indicated in brackets**			
24 epitopes	**B4/1 (AY 161269)**	**F2/1 (AY 180912)**	**A2/1 (AY 377788)**
	aa 36–66 (30–69)	aa 1–9 (1–12)	aa 15–27 (1–31)
		aa 41–53 (38–56)	aa 63–106 (60–109)
			aa 166–176 (166–179)
			aa 239–271 (236–277)
			aa 288–295 (281–305)
			aa 392–399 (389–403)

## Data Availability

All the data is available in the ‘supplementary material’ file as well as with the authors on request.

## Supplementary Materials

The following are available online at https://www.mdpi.com/article/10.3390/vaccines9101058/s1, Figure S1: Sequence alignment and epitope prediction for AY180912(F2/1); Figure S2: Sequence alignment and epitope prediction for AY161269(B4/2); Figure S3: Sequence alignment and epitope prediction for AY377788(A2/1); Figure S4: Selection of epitopes for designing of construct 1 (64 kDa); Figure S5: Selection of epitopes for designing of construct 2 (36 kDa); Figure S6: Selection of epitopes for designing of construct 3 (29 kDa); Figure S7: 64 kDa, 36 kDa & 29 kDa protein structure; Figure S8: Plasmid vector Maps; Figure S9: Linear Plasmid separation on 1.5% Agarose gel; Figure S10: Double digestion of plasmid, product separation on 1.5% Agarose gel; Figure S11: LB agar plates with overnight culture of transformed *E. coli*(M15); Figure S12: 1% Agarose gel separation of Colony PCR products; Figure S13: 12% SDS-PAGE, Coomassie stain and Western Blot using Poly-HIS antibody, for different steps of protein formation and isolation from inclusion bodies of *E. coli*(M15), using 0.1 mM IPTG overnight at 37 °C; Figure S14: Western blot analysis of Recombinant Constructs based on Poly-His antibodies; Figure S15: Gating Strategy For identification of polyfunctional CD3+ T cells; Figure S16: Histogram analysis of IFN-γ, B: TNF-α, C: IL-2 & D: IL-10 secreting CD3+ T cells; Figure S17: Histogram analysis of double positive CD3+ T cells; Figure S18: Bar-graph analysis of double positive CD3^+^ T cells; Figure S19: Giemsa-stained touch impressions of spleen from different animal groups; Figure S20: Representative image for flowcytometry analysis of CFSE signal from adherent peritoneal cells infected with CFSE stained Leishmania donovani; Figure S21: Representative image for flowcytometry analysis for 2,7-dichlorofluorecein levels from peritoneal adherent cells of different animal groups; Figure S22: Relative fold change in gene expression levels of CCR7, IL-23r, CXCR-3, CD-274 & CXCL-9 from immunized and unimmunized animals; Figure S23: IFN-γ levels as measured from the culture supernatant of human PBMC’s stimulated with vaccine candidate proteins, Figure S24: Frequency of T cells positive for polyfunctionality and cytokine secretion when induced with the three proteins. Table S1: Epitope prediction summary; Table S2: Protein and nucleotide sequence of 64 kDa, 36 kDa, and 29 kDa multiepitope constructs; Table S3: Nucleotide Base pair size of inserts and respective final clones; Table S4: Immunization scheme of hamsters; Table S5: Parasite loads and splenic index from the spleen of infected and immunized animals; Table S6: Splenic culture assays for promastigote detection; Table S7: List of Immune-response Genes selected for Quantitative RT-PCR analysis; Table S8: Parasite clearance levels and relative fold change in levels of ROS production at different time intervals from adherent peritoneal cells of immunized and unimmunized hamsters; Table S9: Mean frequency of CD3+ T cells producing the IFN-γ, TNF-α, IL-12 & IL-2 cytokines.
