# Supplementary material for: A T-Cell Epitope-Based Multi-Epitope Vaccine Designed Using Human HLA Specific T Cell Epitopes Induces a Near-Sterile Immunity against Experimental Visceral Leishmaniasis in Hamsters
Source: Vaccines (Basel). 2021 Sep 23;9(10):1058. doi: 10.3390/vaccines9101058 (PMC8537199; doi:10.3390/vaccines9101058)
Supplement: Supplementary file 1 [file vaccines-09-01058-s001.zip › vaccines-1337665-supplementary.pdf]

Supplementary Data

Epitope identification:

Epitopes predicted from both IEDB tools for respective MHC-I and MHC-II alleles fell in an overlapping pattern. For MHC-I & MHC-II alleles 31 & 53 epitopes were detected in AY-161269, 57 & 44 epitopes were present in AY-180912, and 107 & 52 epitopes in AY-377788 respectively. NETMHC-1 and NETMHC-2 predictions remained similar to IEDB with a majority of epitopes overlapping each other. For AY161269 26 & 149 epitopes were detected as MHC-I and MHC-II specific epitopes respectively. For AY-180912 41 & 267 epitopes and AY-377788 cDNA 107 & 400 epitopes were detected for MHC-I and MHC-II alleles respectively **(Supplementary Figures 1-3, Supplementary Table 1)**.

Results obtained from IFNepitope contained 49 epitopes positive for IFN-γ induction in AY-161269, 54 epitopes in AY-180912, and 177 epitopes in AY-377788. Out of which based on Propred and Propred1 for AY-377788, 23 epitopes were MHC-I allele-specific and 129 were MHC-II allele-specific. For AY-161269, 4 were MHC-II allele-specific and 31 were MHC-I allele-specific. For AY-180912, 3 were MHC-II allele-specific while 38 were MHC-I allele-specific.

Multiple epitopes were identified for more than one MHC allele. The majority of epitope sequences predicted by both IEDB and NETMHC for MHC-I and MHC-II had sequence similarities and fell in an overlapping pattern forming epitope-rich regions or islands. More than once the epitope/s were both MHC-I and MHC-II binders along with IFN-γ inducer/s.

Selection of epitopes and designing of construct 1: Construct 1 was designed based on the epitopes predicted as strong binders of respective MHC-I and MHC-II alleles by IEBD and NETMHC-1 and NETMHC-2. Overlaying regions of multiple epitopes were selected to design the construct. The calculated molecular weight of the multiepitope construct was 64458.67 dalton, with a theoretical pI of 6.97. The recombinant construct contained a poly Histidine site (6×His) at the N-terminal for identification purposes. The protein contained a total of 593 amino acids with 65 positively charged and 64 negatively charged. The construct contained no Pyrrolysine Pyl (O) and Selenocysteine Sec (U) amino acids while the Tyrosine Try (Y) was present at a single position (0.2%). **(Supplementary Figure 4, Supplementary Table 2 & 3)**

Selection of epitopes and designing for construct 2: The second design was based on the results of IFNepitope and Propred1. To generate a stronger CTL response, we selected only those IFN-γ epitopes, which were MHC-I or CTL ($CD8^+$ T cells) binders. A total of 63 epitopes were considered out of which 17 were also MHC-II binders. The multiepitope candidate contained a poly His site at the N terminal for identification purposes. The calculated molecular weight of the protein was 36695.70 dalton. The total number of amino acids was 348 with 36 negatively charged and 38 positively charged. The calculated pI value was 8.35. The least numbered amino acid was Cysteine with 2 (0.6%) molecules while Try, Pyl, and Sec didn't occur at all. **(Supplementary Figure 5, Supplementary Table 2 & 3)**

Selection of epitopes and designing for construct 3: Construct 3 was based on the results of IFNepitope, Propred2, and NetChop 3.0. In our attempt to induce only $CD4^+$Tcells only those epitopes which were binders of MHC-II were taken with 24 epitopes in total, spanning 9 epitope regions of overlapping epitopes, out of 24 epitopes 7 were also CTL inducers. The candidate protein contained a poly-HIS site at the N terminal for identification purposes. The theoretical calculated molecular weight was 29007.66 dalton with 256 amino acids. With 33 amino acids

charged negative and 38 as positive, the theoretical pI value was 9.1. Out of 256 amino acids, W and O were absent while U and Y were present three times and C presented itself only once. **(Supplementary Figure 6, Supplementary Table 2 & 3)**

Molecular cloning and protein isolation:

For the generation of multi-epitope protein, the three multiepitope sequences were reverse translated into nucleotide sequences of 756 bp, 1026 bp & 1761 bp size. The inserts were obtained in pMAT and pMK-RQ cloning vectors through Gene Art ThermoFisher. One pMA-T plasmid contained 1761 bp insert, while another contained 756 bp insert, while pMK-RQ contained the 1026 bp insert **(Supplementary Figure 7, Supplementary Table 3)**. All the insert sequences were designed with the addition of BamHI and HindIII cloning sites at 5' & 3' terminals respectively. The inserts were isolated from cloning vectors for subcloning in expression vector pQE30 (Qiagen, Hilden, Germany) **(Supplementary Figure 8 & 9),** by double digesting the pMAT and pMK-RQ plasmids by restriction enzyme BamHI and HindIII (NEB, Ipswich, USA) for 45 minutes at 37°C in HF buffer system (NEB Ipswich, USA). Additionally, pQE30 was also double digested with the same set of enzymes to generate 5' & 3' sticky ends at the insertion site. The double digested products were separated of 1.5% agarose gel by electrophoresis and staining the DNA with Atlas Clearsight DNA stain (Bioatlas Estonia) **(Supplementary Figures 8 - 10)**. Respective bands of 756 base pairs (bp), 1026 bp, and 1761 bp were cut from the gel using sterile surgical blades. The DNA insert was isolated and purified from agarose gel by Monarch® DNA Gel Extraction Kit (NEB, Ipswich, USA) using the manufacture's protocol. The isolated DNA inserts were ligated to pQE30 expression plasmid in a 1:3 vector to insert molar ratio. The ligation was carried overnight at 16°C using T4 ligase (NEB, Ipswich, USA) in the respective buffer system (NEB, Ipswich, USA). The final transformation was carried out in ultracompetent M15 *E. coli* (Qiagen, Hilden, Germany) on ice for 30 minutes. The bacteria were plated on ampicillin and kanamycin LB Agar plates. The plates were placed inverted overnight for colony formation at 37°C. After 12 hrs, the plates were checked for bacterial colonies and random colonies were selected for colony PCR to test transformed bacteria **(Supplementary Figure 11)**.

Random colonies were used to amplify the insert sequence of respective size using primers flanking 5' and 3' insert sites in a PCR setup. Post amplification the PCR product were separated on 1% agarose gel stained with EtBr for detection of individual bands of size, 756+336= 1092 bp, 1026 + 336 = 1362 bp & 1761+336 =2097 bp (336 bp arising as flanking sequences to core sequence) **(Supplementary Figure 12)**. Colonies positive for respective insert were further validated by DNA sequencing. The colonies with confirmed cloning were stored as 40% glycerol stock at -80°C for further use.

Protein expression was induced in the bacteria grown from a single transformed colony at 37°C with 0.1mM IPTG. The protein expressed in inclusion bodies was isolated by solubilizing the inclusion bodies in tris buffer (pH 8). Membrane-bound protein was washed by repeatedly washing in a buffer containing Deoxycholic acid and Tris·HCl with EDTA. Finally, the protein was dissolved in 6M Guanidium HCL. The Guanidium HCL was removed by overnight dialysis against ddH_20. The final product was lyophilized, weighed, and stored at -20°C. The purified product was tested for endotoxin levels using Endotoxin detection kit (THG10-0250, Hi-Media, Mumbai, India), the levels were within an acceptable range i.e., less than 0.25 EU.

The recombinant proteins were confirmed for their molecular weight by 12% SDS-PAGE separation and western blotting using anti-his antibodies. Since the recombinant construct contained a poly Histidine tag at N-terminal, individual bands were visualized by staining with HRP conjugated anti-his antibody **(Supplementary Figure 13 & 14)**.

Supplementary Table 1. Summary of epitopes predicted by IEDB for MHC-I and II, NETMHC-1, NETMHC-2 & IFNepitope. The number of epitopes predicted with starting amino acid position and last amino acid position in the native sequence. In brackets are the number of epitopes from the sequence.

**B4/1 (AY 161269)**	**IEDB MHC-I (number of epitopes 31)**	**NET MHC-I AY (number of epitopes 26)**	**IEDB MHC-II (number of epitopes 53)**	**NET MHC-II (number of epitopes 149)**	**IFNepitope (number of epitopes)**
	aa 1-10 (5)	aa 1-20 (15)	aa 1-88 (53)	aa 1-95(149)	aa 9-110 (49)
	aa 12-83(26)	aa 35 -58(7)			
		aa 71-80(4)			
**F2/1 (AY 180912)**	**IEDB MHC-I (number of epitopes 57)**	**NET MHC-I (number of epitopes 41)**	**IEDB MHC-II (number of epitopes 44)**	**NET MHC-II (number of epitopes 267)**	**IFNepitope (number of epitopes)**
	aa 9-200 (57)	aa 11-33 (5)	aa 22-41(9)	aa 21-55(49)	aa 1-222 (54)
		aa 41-64(10)	aa 48-63 (3)	aa 80-115(90)	
		aa 87-96(2)	aa 83-202 (32)	aa 134-205(128)	
		aa 134-164(6)			
		aa 178-199(10)			
		aa 212-220(8)			
**A2/1 (AY 377788)**	**IEDB MHC-I (number of epitopes 107)**	**NET MHC-I (number of epitopes 118)**	**IEDB MHC-II (number of epitopes 52)**	**NET MHC-II (number of epitopes 400)**	**IFNepitope (number of epitopes)**
	aa 12-19 (1)	aa 14-27(6)	aa 9-45(19)	aa 3-33(61)	aa 7-400 (177)
	aa 26-47(11)	aa 31-65(6)	aa 154-404(33)	aa 56-74(10)	
	aa 55-66(2)	aa 78-102(5)		aa 84-102(14)	
	aa 75-91(3)	aa 125-153(5)		aa 120-223(177)	

	aa 104-112(1)	aa 157-166(1)		aa 250-333(98)	
	aa 139-168(18)	aa 169-178(2)		aa 342-359(4)	
	aa 170-185(4)	aa 189-222(33)		aa 374-408(36)	
	aa 190-272(34)	aa 241-309(18)			
	aa 275-308(26)	aa 342-350(1)			
	aa 364-377(5)	aa 363-402(41)			
	aa 383-399(2)				
**Additional Servers used**	**Propred (MHC-II)**	**Propred-I (MHC-I)**	**CTLpred**		
**B4/1 (AY161269)**	aa 36-66 (4)	aa 12-110 (31)	aa 37-45 (3)		
**F2/1 (AY180912)**	1-9, 41-54 (3)	aa 01-222 (38)	aa 01- 222 (12)		
**A2/1 (AY377788)**	aa 15- 400 (23)	aa 15- 400 (129)	aa 15- 400 (26)		

Supplementary Table 2. Protein and nucleotide sequence of 64 kDa, 36 kDa, and 29 kDa multiepitope constructs.

>Candidate_1_64_kDa

HHHHHHGSMSAETLACTYAALMLSDAGLPTSAENIAAAVKAAGVEMRPTLPIIFARFLEKKSVETLMAAA
AAQAPTAASAPSPAAGAASAAGGKREYEERLVDCLTKYSCVLFVGMDNVRSQQVHDVRRALRGKAEFIM
GKKTLQAKIVEKGGKRFNDQCEEYNLLSGNTGLIFTNNAVQEITSVLDAHRVKAPARVGAISPCDVVVPAG
STGMEPTQTSFFQALNIATKIAKGMVEIVTEKKVLSVGDKVDNSTATLLQKLNISPFYYQVNVLSVWDRGV
LFGGHRKLVFSFIRMIRRSQVENPERAEAVAQMLGEEFGVDPAGSGGLHDTEVDVLEAFKTALREHERRSG
VEQDEKFVCFVELLEKKGYFKGVEKGGPYEGLTAEQIKNKGNELMSQAKYKEAIAYYTKAIELQPDNAVFF
ANRAAAHTHLKDYNNAIIDCERAIIINPEYSKSYSRLGTALFYQENYSRAVDAFTGGAEEKAKATALSTGGG
MGGFPGMGGFPEMGGMPDMSQFANMMSNPQFMETAQRMMQNPEFSNLVANMASKGGKLAGIMADVQA
NGYGAFQKYLGDPDVMNLMMKFQNLMFTNKL

>Candidate_1_64_kDa

GGA TCC ATG AGC GCT GAA ACA TTG GCT TGT ACT TAC GCC GCA CTG ATG CTG TCT GAT GCC GGG
CTT CCG ACA TCA GCC GAG AAC ATT GCC GCG GCT GTA AAA GCTGCG GGC GTT GAA ATG CGT
CCC ACG CTT CCT ATT ATT TTC GCC CGT TTC CTT GAA AAG AAG TCG GTT GAA ACT TTG ATG GCA
GCC GCC GCA GCT CAG GCCCCA ACA GCA GCT AGT GCT CCA AGC CCT GCC GCC GGT GCT GCC TCT
GCG GCA GGT GGA AAA CGC GAG TAC GAG GAG CGT CTT GTA GAT TGC TTA ACT AAATAT AGC
TGC GTT CTG TTT GTT GGG ATG GAC AAC GTG CGC AGT CAA CAA GTG CAC GAT GTT CGT CGC GCA
CTG CGT GGC AAG GCT GAG TTC ATT ATG GGGAAA AAG ACC TTA CAA GCA AAA ATT GTT GAA
AAA GGG GGA AAA CGC TTC AAC GAT CAG TGC GAA GAA TAC AAC CTG TTG TCC GGA AAC ACC
GGT TTA ATTTTT ACC AAT AAT GCA GTT CAG GAA ATT ACC AGC GTC TTA GAT GCT CAC CGC GTC
AAA GCT CCC GCC CGC GTG GGA GCG ATT TCT CCT TGT GAT GTA GTAGTC CCT GCG GGT AGC ACG
GGG ATG GAG CCT ACC CAA ACA TCC TTT TTC CAA GCC TTG AAT ATT GCG ACT AAG ATT GCG AAA
GGC ATG GTA GAA ATC GTGACA GAG AAG AAG GTG CTT AGC GTC GGA GAT AAA GTA GAC AAT
TCG ACA GCA ACG TTG TTG CAG AAG TTG AAT ATT TCC CCC TTT TAC TAC CAA GTG AACGTT CTG
TCG GTC TGG GAC CGC GGT GTG TTG TTC GGA GGC CAC CGC AAA TTA GTC TTT AGT TTT ATT CGC
ATG ATC CGC CGC AGC CAA GTG GAG AAC CCGGAG CGC GCT GAG GCG GTC GCA CAG ATG CTT
GGG GAG GAA TTT GGC GTC GAT CCT GCG GGA AGT GGG GGC TTG CAT GAC ACT GAG GTT GAT
GTG CTT GAGGCC TTT AAA ACG GCG CTG CGC GAG CAT GAA CGC CGC TCA GGA GTT GAA CAG
GAC GAG AAG TTC GTC TGT TTT GTG GAG TTA CTT GAA AAG AAG GGA TATTTC AAG GGG GTA
GAA AAG GGC GGG CCG TAT GAG GGG TTA ACA GCG GAA CAA ATC AAG AAC AAA GGC AAT GAA
CTG ATG TCG CAA GCC AAG TAC AAA GAGGCC ATC GCT TAT TAT ACG AAG GCA ATC GAG TTA
CAG CCT GAC AAT GCG GTT TTC TTT GCG AAC CGT GCA GCT GCA CAC ACA CAC TTA AAG GAT
TAC AATAAC GCT ATT ATT GAT TGC GAG CGT GCT ATC ATC ATC AAC CCC GAA TAT AGT AAA TCC
TAT AGC CGC TTG GGG ACC GCT TTG TTT TAT CAG GAA AAC TACTCA CGC GCG GTC GAC GCC TTT
ACT GGA GGT GCG GAG GAA AAA GCT AAG GCC ACA GCA TTG AGC ACT GGC GGT GGT ATG GGC
GGA TTT CCT GGG ATG GGAGGA TTC CCA GAA ATG GGG GGA ATG CCC GAT ATG TCG CAA TTC
GCA AAT ATG ATG TCG AAC CCA CAA TTC ATG GAG ACG GCA CAG CGT ATG ATG CAA AATCCT
GAG TTT AGC AAC CTT GTA GCT AAT ATG GCG AGC AAA GGT GGT AAG TTG GCA GGA ATC ATG
GCG GAC GTG CAA GCA AAC GGC TAC GGA GCA TTT CAGAAG TAC CTG GGC GAC CCG GAC GTT
ATG AAT TTA ATG ATG AAG TTC CAG AAC TTA ATG TTC ACC AAC AAG CTT

>Candidate_2_36_kDa

HHHHHHGSVRRALRGKAEFIMGKKTLGGNISPFYYQVNVGGSAENIAAAVKAAGVEMRPTLPIIFAR
FLEKGGAASAPSPAAGAASAAAAGGKVGGEEGDDDMGFGLGGKLVFSFIRMIRRSQVGGVDPAGSG
GLHDTEVDVLEAFKTALGGQDEKFVCFVELLEKGGFKGVEKGSDEYAQRLEKAREKFGGNELMSQA
KYKEAIAYYTKAIELQPDNAVFFANRAAAHTHLGGIIDCERAIIINPEYSKSYSRLGTALFYQENYSRA
VDAFTKAGGAEEKAKATALSTGGGMGGFPGMGGFPEMGGMPDMSQFANMMSGGVQANGYGAFQ
KYLGGGLMMKFQNLMKL

>Candidate_2_36_kDa

GGA TCC GTG CGC CGT GCG TTA CGT GGT AAG GCT GAA TTT ATC ATG GGT AAG AAG ACA CTG GGG
GGA AAT ATC AGT CCG TTT TAC TAT CAA GTG AAT GTG GGT GGTTCT GCC GAG AAC ATT GCA GCA GCG
GTC AAA GCC GCC GGC GTT GAG ATG CGC CCC ACG TTA CCC ATC ATC TTC GCA CGT TTT CTT GAG AAG
GGC GGC GCAGCC TCC GCT CCG TCA CCA GCA GCC GGA GCG GCC AGC GCT GCG GCA GCA GGT GGA
AAG GTC GGC GGT GAG GAG GGC GAC GAT GAT ATG GGC TTC GGC TTAGGG GGA AAA TTG GTG TTC
TCA TTC ATT CGC ATG ATT CGC CGT TCT CAA GTG GGT GGC GTG GAC CCA GCG GGC TCG GGG GGA TTA
CAT GAC ACG GAA GTGGAC GTG TTA GAA GCG TTT AAG ACG GCA CTG GGC GGC CAG GAC
GAA AAG TTT GTC TGT TTT GTA GAG CTG TTG GAA AAA GGT GGT TTT AAA GGA GTA GAAAAA
GGG TCT GAT GAG TAT GCC CAG CGT CTT GAG AAG GCT CGC GAA AAG TTC GGC GGG AAT
GAG CTG ATG TCT CAA GCA AAG TAC AAG GAA GCG ATC GCCTAC TAC ACA AAA GCG ATC
GAA TTA CAA CCG GAC AAT GCA GTC TTT TTT GCT AAC CGC GCA GCG GCC CAT ACA CAT
TTA GGC GGT ATC ATC GAT TGT GAACGT GCG ATT ATT ATT AAT CCT GAA TAT TCC AAA TCC
TAT TCG CGC TTA GGG ACT GCG CTT TTC TAT CAG GAG AAT TAT TCT CGT GCT GTA GAT GCT
TTTACT AAG GCA GGC GGA GCA GAG GAA AAA GCT AAA GCG ACC GCT CTG TCC ACC GGG
GGC GGT ATG GGC GGA TTC CCT GGT ATG GGA GGA TTT CCT GAG ATGGGA GGT ATG CCA
GAT ATG TCG CAG TTC GCT AAC ATG ATG TCT GGT GGA GTC CAA GCG AAT GGT TAT GGA
GCC TTC CAA AAA TAT CTG GGT GGA GGA TTGATG ATG AAA TTT CAG AAT TTG ATG AAG CTT

>Candidate_3_29_kDa

HHHHHHGSMEERDLPITDDHRKLVFSFIRMIRRSQVENPVDVLEAFKTALREHERRSGVEQDEKFVCF
VELLEKKGYFKGVEKGSDEYAVFFANRAAAHTHLKKEDLKRAEEKAKATALSTGGGMGGFPGMGGF
PEMGGMPDMSQMMSNPQFMETAQRMMQNPEFSNLVAVMNLMMKFQNLMFTNMPSITTAKREYEV
HDVRRALRGKAEFIMGKKAVKAAGVEMRPTLPIIFARFLEKKSVETLMAAAAAQAPTAKL

>Candidate_3_29_kDa

GGATCCATGGAAGAACGTGATCTGCCGATTACAGATGATCACCGTAAACTGGTGTTTAGCTTTATT
CGTATGATTCGTCGTAGCCAGGTTGAAAATCCGGTTGATGTTCTGGAAGCATTTAAAACCGCACTG
CGTGAACATGAACGTCGTAGCGGTGTTGAACAGGATGAAAAATTTGTTTGCTTTGTCGAACTGCTG
GAAAAAAAAGGCTATTTCAAAGGTGTGGAAAAAGGCAGTGATGAATATGCCGTTTTTTTGCAAA
TCGTGCAGCAGCACATACCCACCTGAAAAAAGAAGATCTGAAACGCGCAGAAGAAAAAGCAAAA
GCAACCGCACTGAGCACCGGTGGTGGTATGGGTGGCTTTCCGGGTATGGGAGGTTTTCCGGAAAT
GGGTGGTATGCCGGATATGAGCCAGATGATGAGCAATCCGCAGTTTATGGAAACCGCACAGCGTA
TGATGCAGAATCCGGAATTTTCAAATCTGGTTGCCGTTATGAACCTGATGATGAAATTTCAGAACC
TGATGTTTACCAACATGCCGAGCATTACCACCGCAAAACGTGAATATGAAGTTCATGATGTTCGTC
GTGCACTGCGTGGTAAAGCAGAATTCATTATGGGTAAAAAAGCCGTTAAAGCAGCCGGTGTTGAA
ATGCGTCCGACCCTGCCGATTATCTTTGCACGTTTTCTCGAGAAAAAAAGCGTTGAAACCCTGATG
GCAGCAGCAGCAGCCCAGGCACCGACCGCAAAGCTT

Supplementary Table 3. Nucleotide Base pair size of inserts and respective final clones.

Multiepitope Construct	Nucleotide size (base pair)	Cloning vector Size (base pair)	Fragment + pQE30 (base pair)
29 kDa	756	3130	4175
36 kDa	1026	3304	4445
64 kDa	1761	4135	5180

Supplementary Table 4. Immunization scheme of hamsters. CFA: Complete Freund's Adjuvant. iFA: Incomplete Freund's Adjuvant. PBS: Phosphate Buffer Saline.

**Groups (n=8)**	Day 1	Day 14	Day 28	Day 42	Day 102
**64 kDa**	rAg+CFA	rAg+iFA	rAg+PBS	1×10^7 amastigotes	sacrifice
**36 kDa**	rAg+CFA	rAg+iFA	rAg+PBS	1×10^7 amastigotes	sacrifice
**29 kDa**	rAg+CFA	rAg+iFA	rAg+PBS	1×10^7 amastigotes	sacrifice

**Adjuvant control**	CFA	iFA	PBS	1×10^7 amastigotes	sacrifice
**Positive control**	PBS	PBS	PBS	1×10^7 amastigotes	sacrifice
**Negative control**	PBS	PBS	PBS	PBS	sacrifice

Supplementary Table 5. Parasite loads and splenic index from the spleen of infected and immunized animals.

**Group (n=8)**	**Mean LDU (Amount of amastigotes/Number of Nucleated cells) × Weight of organ(mg)).** Mean values ±SEM	**Mean Splenic Index**<br>**Splenic Index is measured as the ratio of spleen weight to whole body weight multiplied by a hundred.** Mean values ±SEM
**Unimmunized-Infected**	4277.3 ±661.1	0.322 ±0.0567
**CFA only**	4602.6 ±931.02 ns (*p*>0.9999)	0.368 ± 0.0534 ns (*p*=0.3402)
**Immunized with 64 kDa**	178.71 ±47.03*** (*p*=0.0002)	0.145 ±0.048** (*p*=0.0050)
**Immunized with 36 kDa**	332.2 ±105.32*** (*p*=0.0002)	0.216 ±0.067* (*p*=0.0303)
**Immunized with 29 kDa**	478.5 ±50.10*** (*p*=0.0002)	0.151 ±0.013** (*p*=0.0012)
**Healthy**	NA	0.168 ± 0.0548

Significant difference determined by Student's t-test, * $p < 0.05$ compared to unimmunized-infected group. Level of significance, n.s. $p>0.05$, * $p\leq0.05$, ** $p\leq0.01$, *** $p\leq0.001$

Supplementary Table 6. Splenic culture assays for promastigote detection. n=8. Mean count ±SD. The difference between groups was statistically significant at $p<0.0001$ as determined by ANOVA. *Dunnett's multiple comparisons test, (Level of significance, ns $p>0.05$, * $p\leq0.05$, ** $p\leq$ 0.01, *** $p\leq0.001$, **** $p\leq0.0001$)

	**Infected**	**CFA only**	**64 kDa**	**36 kDa**	**29 kDa**
**0**	11.11 ±26.67	8.89 ±14.53	0 ±0	0 ±0	0 ±0
**5**	184.44 ±120.4	104.44 ±74.68	0 ±0	2.22 ±6.67	0.11 ±0.33
**7**	602.22 ±432.60	395.56 ±269.23	8.89 ±14.53****	57.78 ±104.137****	104.44 ±253.77****
**14**	1162.22 ±385.8	884.44 ±304.27**	26.66 ±56.57****	151.11 ±159.72****	226.67 ±253.77****

Supplementary Table 7. List of Immune-response Genes selected for Quantitative RT-PCR analysis.

Gene	5'-3' sequence	amplicon size
γ-actin F	ACA GAG AGA AGA TGA CGC AGA TAA TG	70 bp
γ-actin R	GCC TGA ATG GCC ACG TAC A	
IL-10 F	GGT TGC CAA ACC TTA TCA GAA ATG	194 bp
IL-10 R	TTC ACC TGT TCC ACA GCC TTG	
IL-4	ACA GAA AAA GGG ACA CCA TGC A	95 bp
IL-4 R	GAA GCC CTG CAG ATG AGG TCT	
IFN-γ F	TGT TGC TCT GCC TCA CTC AGG	130 bp
IFN-γ R	AAG ACG AGG TCC CCT CCA TTC	
CCR7 F	TTGGCAGAAGCACACCTGAT	107 bp
CCR7 R	CAAATCCGTGATGGAACCGC	
TGF-β1 F	GGC TAC CAC GCC AAC TTC TG	81 bp
TGF-β1 R	GAG GGC AAG GAC CTT ACT GTA CTG	
TBE21 F	ACCGGGACCCAATTGTCAAC	96 bp
TBE21 R	GGAAGGTCGGGGTAGAAACG	
TNF-α F	TGA GCC ATC GTG CCA ATG	79 bp
TNF-α R	AGC CCG TCT GCT GGT ATC AC	
IL-12p40 F	AAT GCG AGG CAG CAA ATT ACT C	88 bp
IL-12p40 R	CTG CTC TTG ACG TTG AAC TTC AAG	
L Sell F	GGTTCACGTTAGGCAGACCT	129 bp
L Sell R	GGCTCCTGGAAGACAATGGAA	
CXCR-3 F	TGTTCAGTGGGTTTTCGGCT	136 bp
CXCR-3 R	GATCTGGGTGGCATGCACTA	
IL-21 F	GGACAGTGGCCCATAAAACAAG	80 bp
IL-21 R	TTCAACACTGTCTATAAGATGACGAAGTC	
CXCL-9 F	AATTCTGTTGGTGGAGGCGT	84 bp
CXCL-9 R	TCACCGGACCTCCTCTTCTC	

CD274 F	TTGCTAAATGCCCCATACCGA	70 bp
CD274 R	GTTCATGCTCGGACGTGACT	
IL23r F	TTTGGGGAACCTCATGCCTC	119 bp
IL23r R	ACTTTGCTGGGGACATCTGG	

Supplementary Table 8. Parasite clearance at different time intervals from adherent peritoneal cells of immunized and unimmunized hamsters. Relative fold change in levels of ROS production in peritoneal cells from immunized and unimmunized hamsters. For Parasite clearance, the significant difference was determined by Student's t-test between the healthy group and the test group. For RPI significant difference determined by Student's t-test between immunized vs unimmunized-infected group (Level of significance, ns $p>0.05$, *$p\leq0.05$, **$p\leq0.01$, ***$p\leq0.001$).

	**Parasite Clearance, measured as MFI value of CFSE signal intensity from Macrophages. Mean values ±SEM**			**ROS levels as measured by MFI value of DCF signal intensity from Macrophages. Values expressed a ROS Production Index. RPI= (MFI test group/MFI healthy group) Mean RPI values ±SEM**	
**Group (n=8)**	**Mean MFI at 12 hrs**	**Mean MFI at 24 hr**	**Mean MFI at 48 hr**	**12 hrs Mean relative fold change in ROS levels**	**24 hrs Mean relative fold change in ROS levels**
**Infected**	55.33 ±3.9	59.7 ±8.2	33.48 ±12.02	0.54 ±0.081	0.31 ±0.09
**CFA**	42.2 ±4.79 * (*p* =0.049)	86.86 ±7.05	36.27 ±8.7	0.68 ±0.302	0.52 ±0.10
**64 kDa**	19.26 ±1.46 ***(*p*=0.0002)	8.14 ±0.96 ***(*p*=0.0002)	10.58 ±2.39***(*p*=0.0002)	2.24 ±1.12 *** (*p*=0.0011)	1.23 ±0.68 *(*p*=0.0281)
**36 kDa**	16.39 ±2.16 ***(*p*=0.0002)	27.6 ±5.9 *(*p*=0.0193)	6.41 ±1.63 ***(*p*=0.0002)	2.33 ±0.8	1.11 ±0.32 **(*p*=0.0019)
**29 kDa**	26.18 ±2.0 ***(*p*=0.0002)	17.7 ±1.59 **(*p*=0.0011)	14.36 ±1.80 ***(*p*=0.0002)	4.44 ±1.92	0.93 ±0.31 *(*p*=0.0148)

**Healthy**	68.5 ±3.2	68.63 ±5.2	41.14 ±5.93	NA	NA

Supplementary Table 9. Mean frequency of CD3+ T cells producing the IFN-γ, TNF-α, IL-12 & IL-2 cytokines.

**Responses**				The average percentage of total CD3+T cells. T-test for statistical analysis done between LPSA and Protein (64 kDa, 36 kDa, and 29 kDa) stimulated groups. n=4. Mean values ± SD. Significance level determined by Student's t-test, *p < 0.05, compared to LPSA treated group. Level significance, ns $p > 0.05$, * $p \leq 0.05$, ** $p \leq 0.01$.		
	**LPSA**	**64 kDa**	**36 kDa**	**29 kDa**	**PMA ionomycin**	**PHA**
**Polyfunctional T cells IFN-γ TNF-α & IL-2 secretory**	0.55 ±0.07	0.49 ±0.105	0.96 ±0.47	0.78 ±0.35	0.46 ±0.11	0.86 ±0.64
**IFN-γ & TNF-α secretory**	0.53 ±0.11	3.97 ±0.71*(*p*=0.0286)	3.39 ±1.4*(*p*=0.0286)	1.67 ±0.20*(*p*=0.0286)	3.95 ±0.11*(*p*=0.0286)	1.5 ±1.0*(*p*=0.0286)
**IFN-γ**	1.87 ±0.23	8.27 ±0.88*(*p*=0.0286)	9.85 ±1.54*(*p*=0.0286)	5.57 ±0.52*(*p*=0.0286)	8.8 ±1.3*(*p*=0.0286)	5.5 ±1.96*(*p*=0.0286)
**TNF-α**	5.32 ±0.8	20.9 ±2.4** (*p*=0.0079)	18 ±5.03** (*p*=0.0079)	7.35 ±3.06	21.9 ±2.56**(*p*=0.0079)	10.7 ±2.82**(*p*=0.0079)
**IL-2**	2.09 ±0.7	4.30 ±0.37* (*p*=0.0286)	9.24 ±1.4* (*p*=0.0286)	3.9 ±0.93	3.85 ±1.2	5.96 ±4.9
**IL-10**	1.2 ±0.29	.94 ±0.33	1.39 ±0.57	3.29 ±0.38* (*p*=0.0286)	1.7 ±1.19	4.09 ±1.81*(*p*=0.0286)

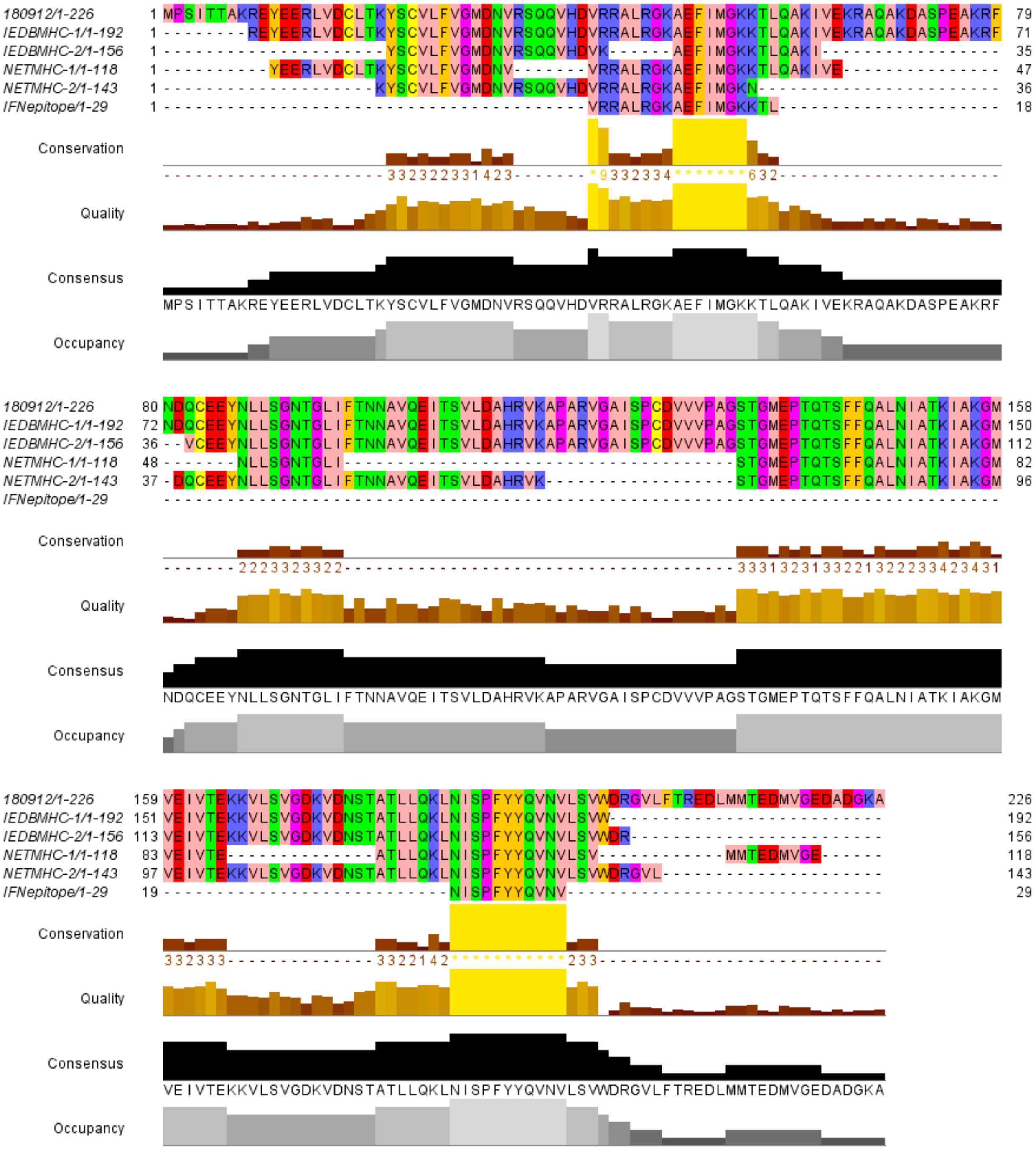

Supplementary Figure 1. Sequence alignment and epitope prediction for AY180912(F2/1). From top, Protein sequence AY180912, followed by T cell epitopes predicted by IEDB(MHC-I), IEDB(MHC-II), NETMHC-I, NETMHC-II & IFNepitope. Sequence alignment by Clustal Omega (EMBL-EBI).

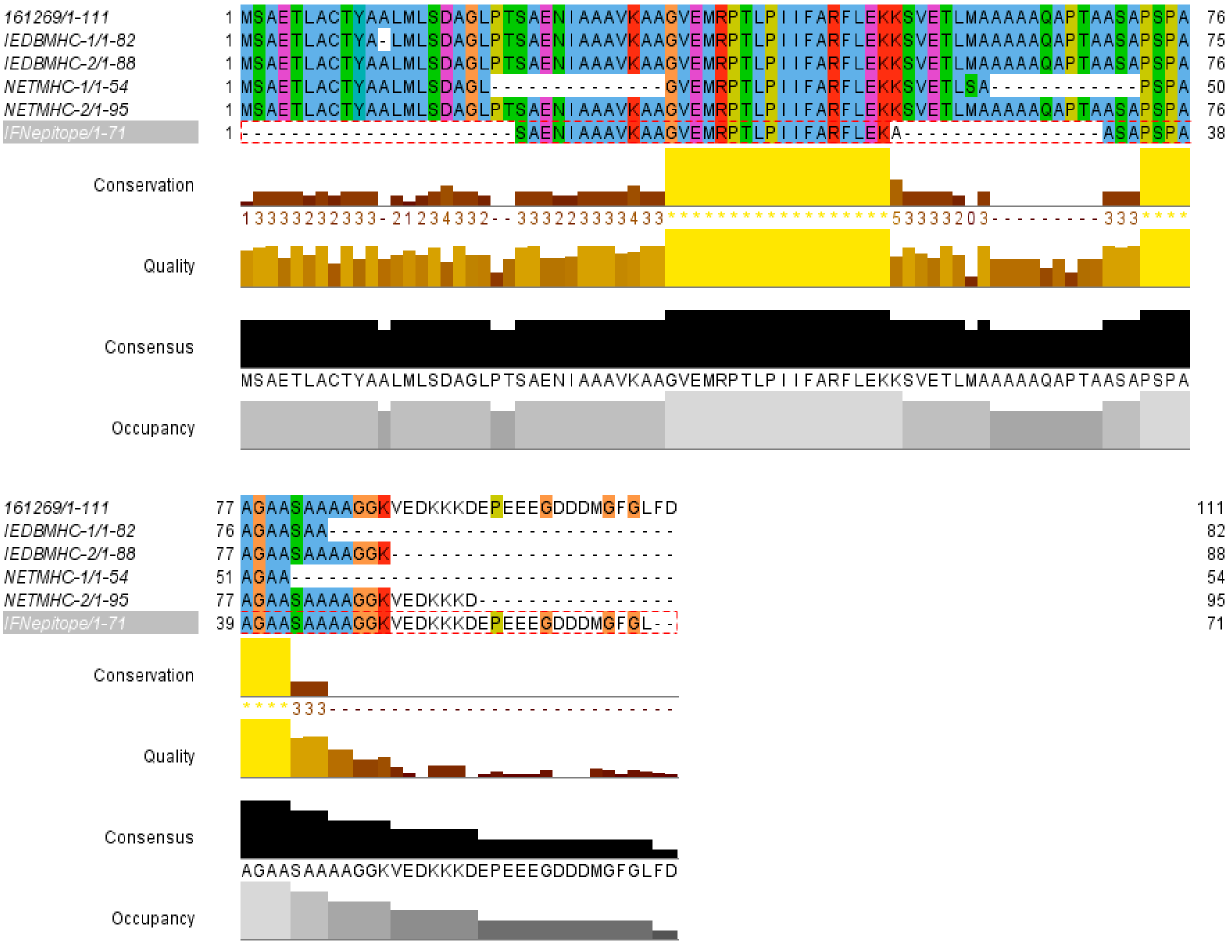

Supplementary Figure 2. Sequence alignment and epitope prediction for AY161269(B4/2). From top, Protein sequence AY161269, followed by T cell epitopes predicted by IEDB(MHC-I), IEDB(MHC-II), NETMHC-1, NETMHC-2 & IFNepitope. Sequence alignment by Clustal Omega (EMBL-EBI).

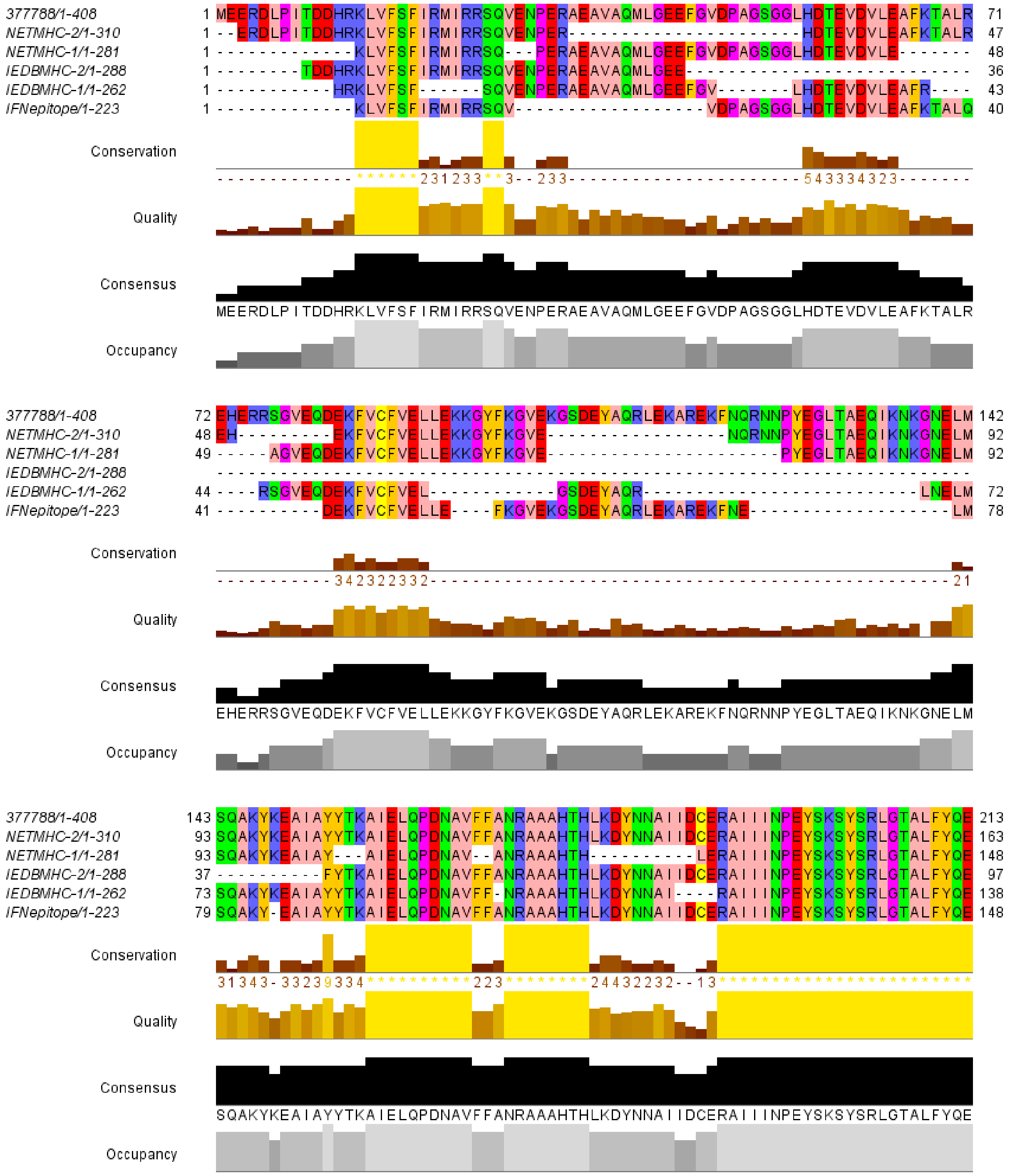
377788/1-408
NETMHC-2/1-310
NETMHC-1/1-281
IEDBMHC-2/1-288
IEDBMHC-1/1-262
IFNepitope/1-223
Conservation
Quality
Consensus
Occupancy

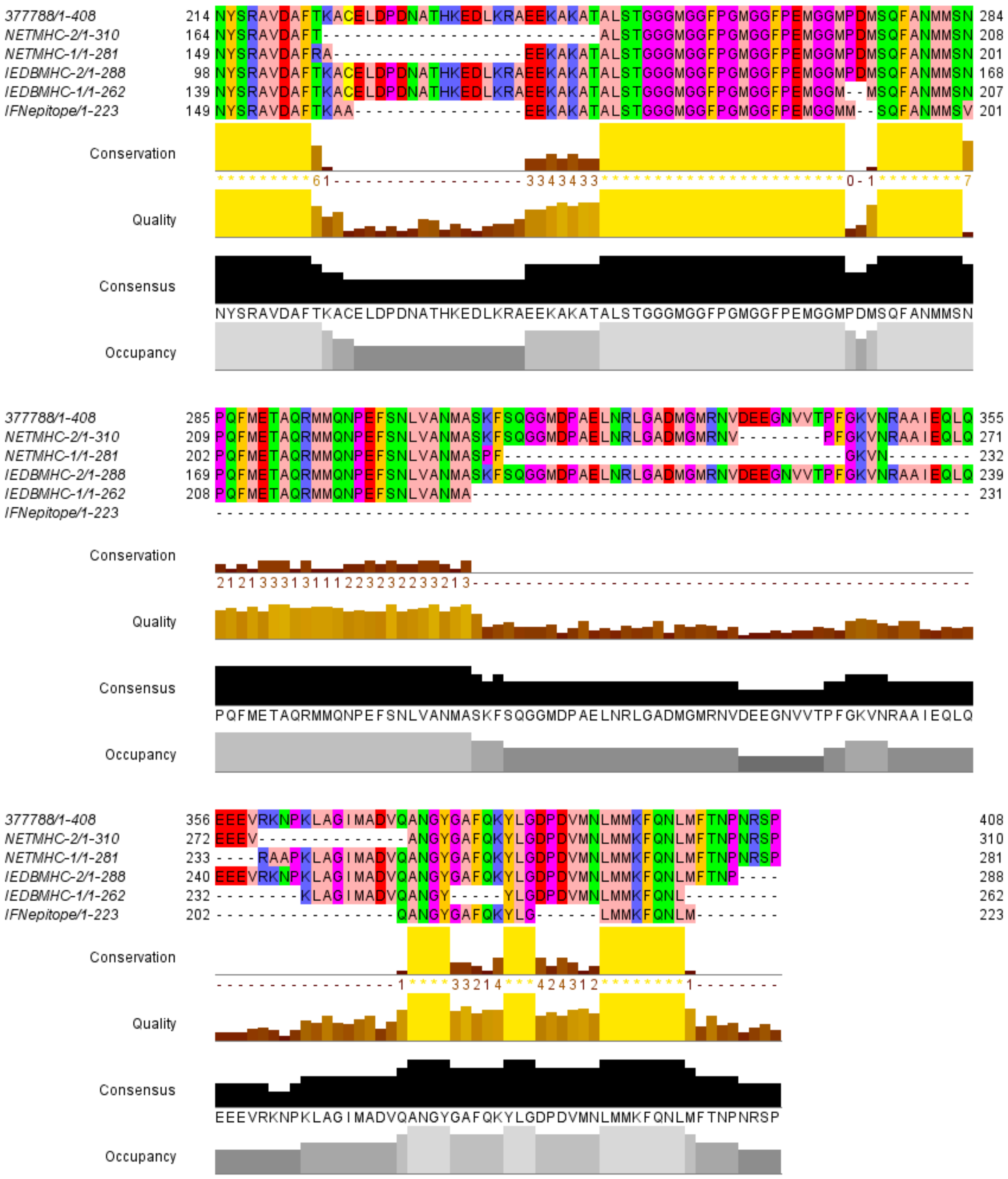

Supplementary Figure 3. Sequence alignment and epitope prediction for AY377788(A2/1). From top, Protein sequence AY377788, followed by T cell epitopes predicted by IEDB(MHC-I), IEDB(MHC-II), NETMHC-1, NETMHC-2 & IFNepitope. Sequence alignment by Clustal Omega (EMBL-EBI).

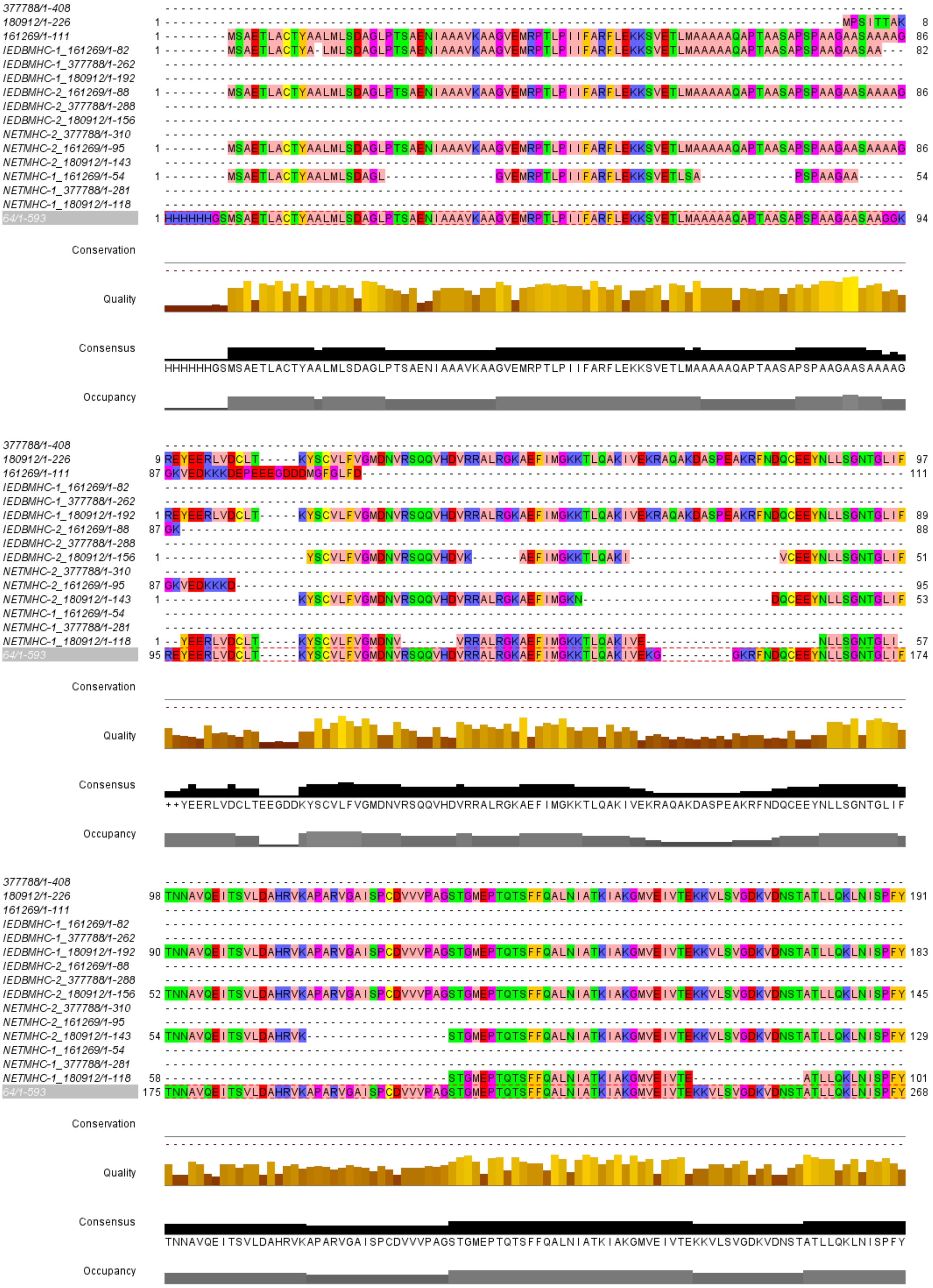
377788/1-408
180912/1-226
161269/1-111
IEDBMHC-1_161269/1-82
IEDBMHC-1_377788/1-262
IEDBMHC-1_180912/1-192
IEDBMHC-2_161269/1-88
IEDBMHC-2_377788/1-288
IEDBMHC-2_180912/1-156
NETMHC-2_377788/1-310
NETMHC-2_161269/1-95
NETMHC-2_180912/1-143
NETMHC-1_161269/1-54
NETMHC-1_377788/1-281
NETMHC-1_180912/1-118
64/1-593
Conservation
Quality
Consensus
HHHHHHGSMSAETLACTYAALMLSDAGLPTSAENIAAAVKAAGVEMRPTLPIIFARFLEKKSVETLMAAAAAQAPTAASAPSPAAGAASAAAAG
Occupancy
++YEERLVDCLTEEGDDKYSCVLFVGMDNVRSQQVHDVRRALRGKAEFIMGKKTLQAKIVEKRAQKDASPEAKRFNDQCEEYNLLSGNTGLIF
TNNAVQEITSVLDAHRVKAPARVGAISPCDVVVPAGSTGMEPTQTSFFQALNIATKIAKGMVEIVTEKKVLSVGDKVDNSTATLLQKLNISPFY

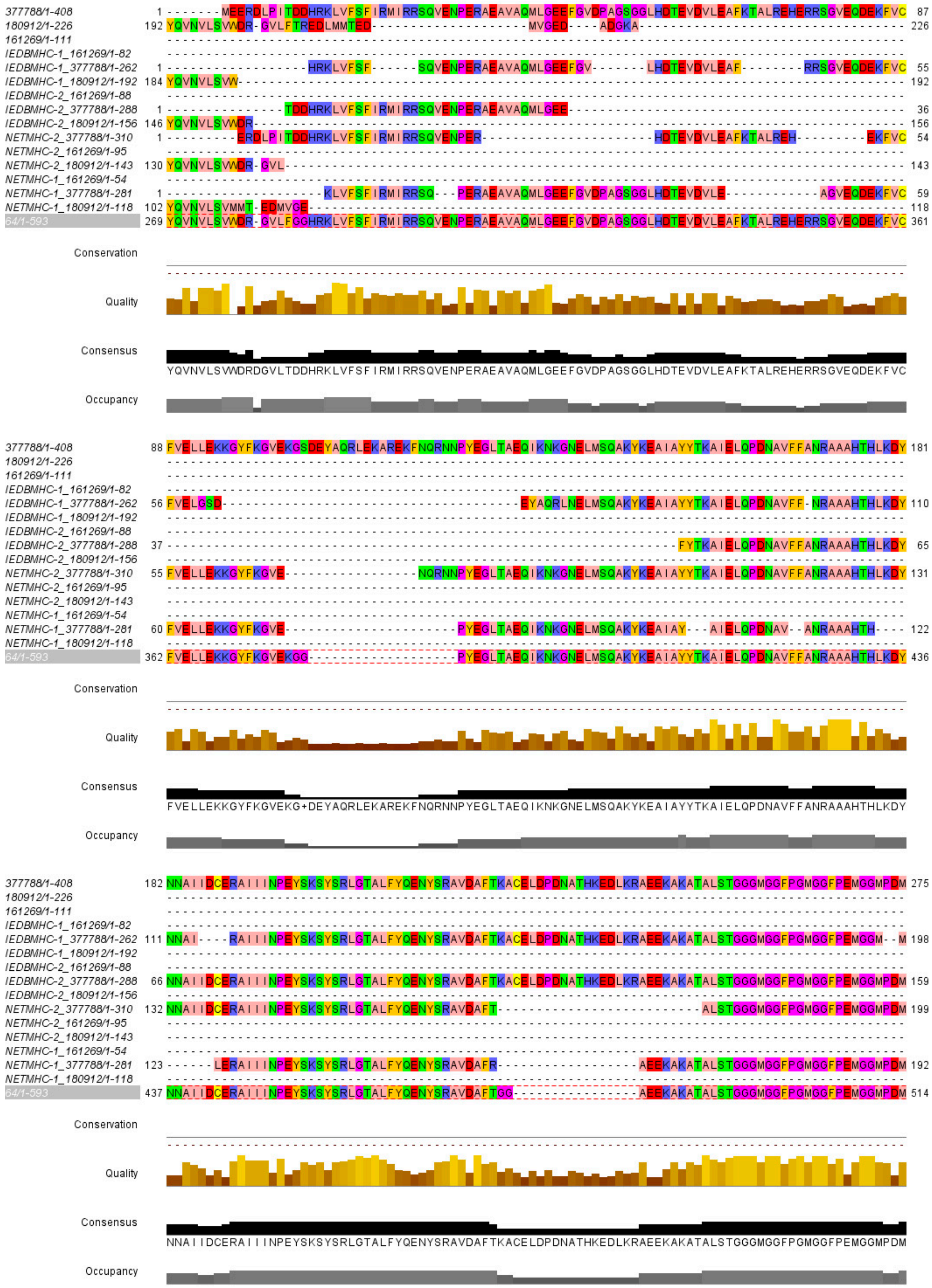
377788/1-408
180912/1-226
161269/1-111
IEDBMHC-1_161269/1-82
IEDBMHC-1_377788/1-262
IEDBMHC-1_180912/1-192
IEDBMHC-2_161269/1-88
IEDBMHC-2_377788/1-288
IEDBMHC-2_180912/1-156
NETMHC-2_377788/1-310
NETMHC-2_161269/1-95
NETMHC-2_180912/1-143
NETMHC-1_161269/1-54
NETMHC-1_377788/1-281
NETMHC-1_180912/1-118
64/1-593
Conservation
Quality
Consensus
YQVNVLSWDRDGVLTDDHRKLVFSFIRMIRRSQVENPERAEAVAQMLGEEFGVDPAGSGGLHDTEVDVLEAFKTALREHERRSGVEQDEKFVC
Occupancy
FVELLEKKGYFKGVEKG+DEYAQRLEKAREKFNQRNNPYEGLTAEQIKNKGNELMSQAKYKEAIAYYTKAIELQPDNAVFFANRAAAHTHLKDY
NNAIIDCERAIIINPEYSKSYSRLGTALFYQENYSRAVDAFTKACELDPDNATHKEDLKRAEEKAKATALSTGGGMGGFPGMGGFPEMGGMPDM

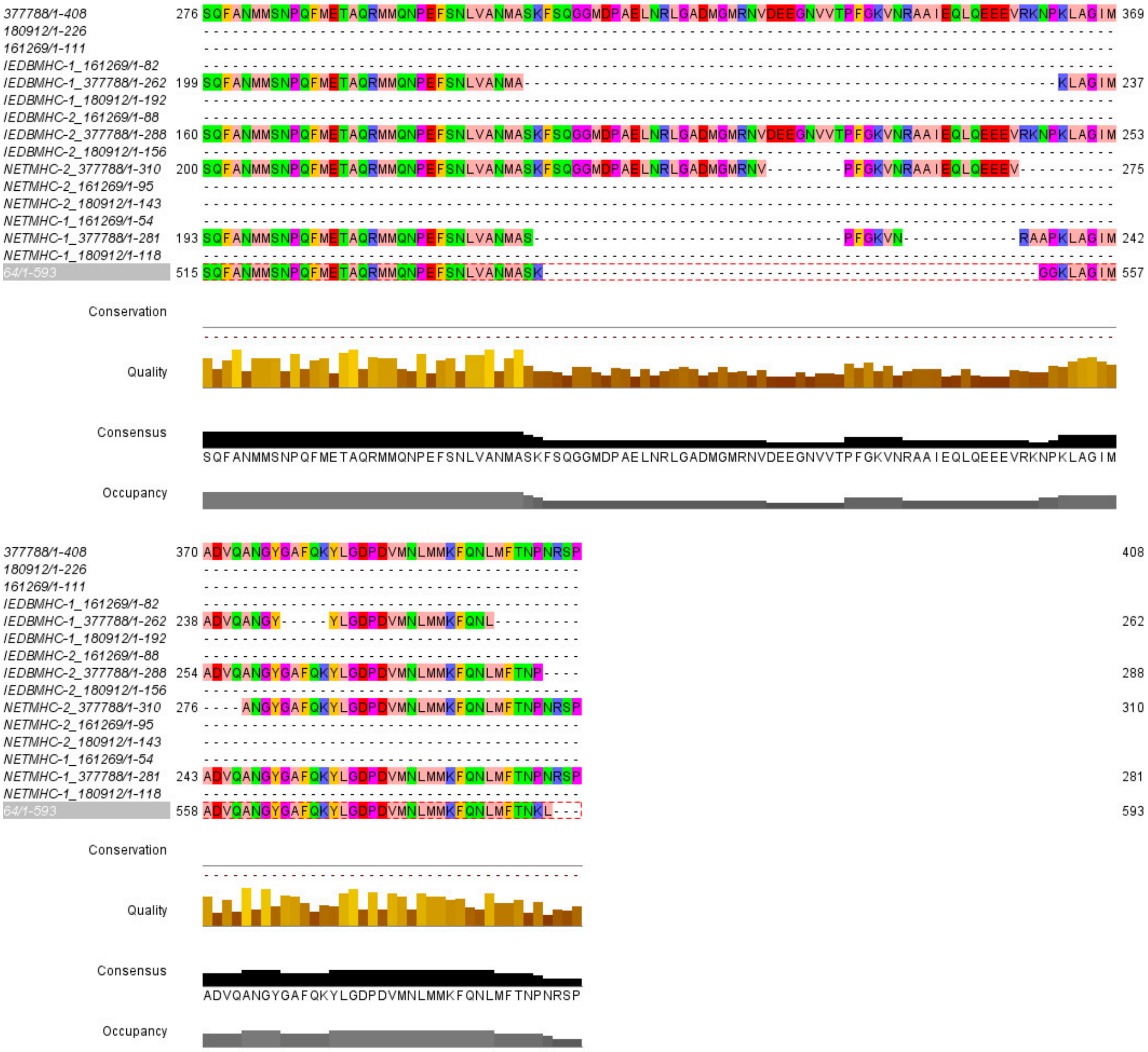

Supplementary Figure 4. Selection of epitopes for designing of construct 1 (64 kDa). Protein sequence alignment of 3 genes (A2/1, B4/1 & F2/1) with respective epitope prediction by IEDB MHC-I IEDB-MHC-II NETMHC-I & NETMHC-II for each gene, followed by selected sequences for multiepitope 64 kDa protein. Sequence alignment by Clustal Omega (EMBL-EBI).

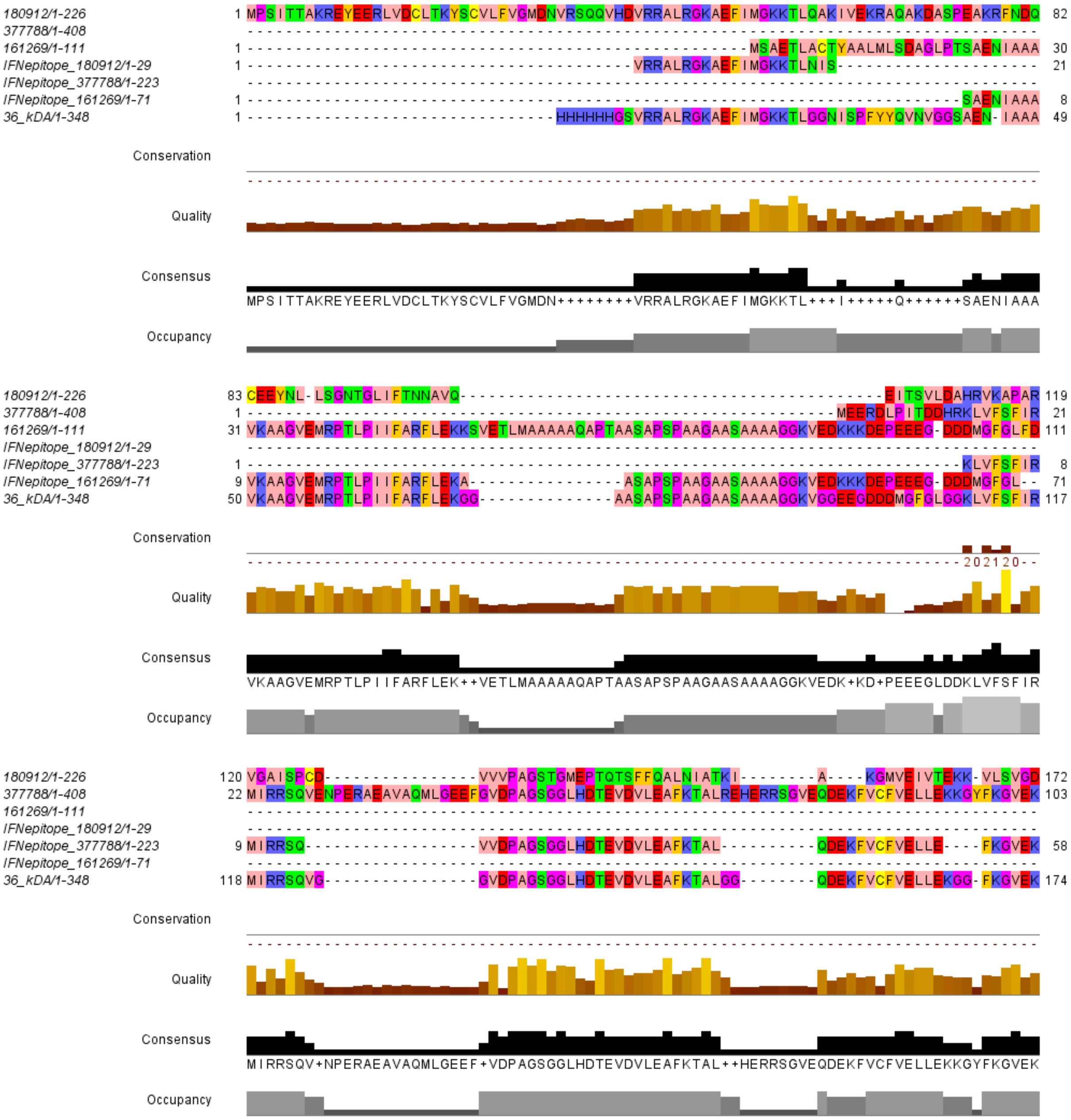
180912/1-226
377788/1-408
161269/1-111
IFNepitope_180912/1-29
IFNepitope_377788/1-223
IFNepitope_161269/1-71
36_kDA/1-348
Conservation
Quality
Consensus
Occupancy

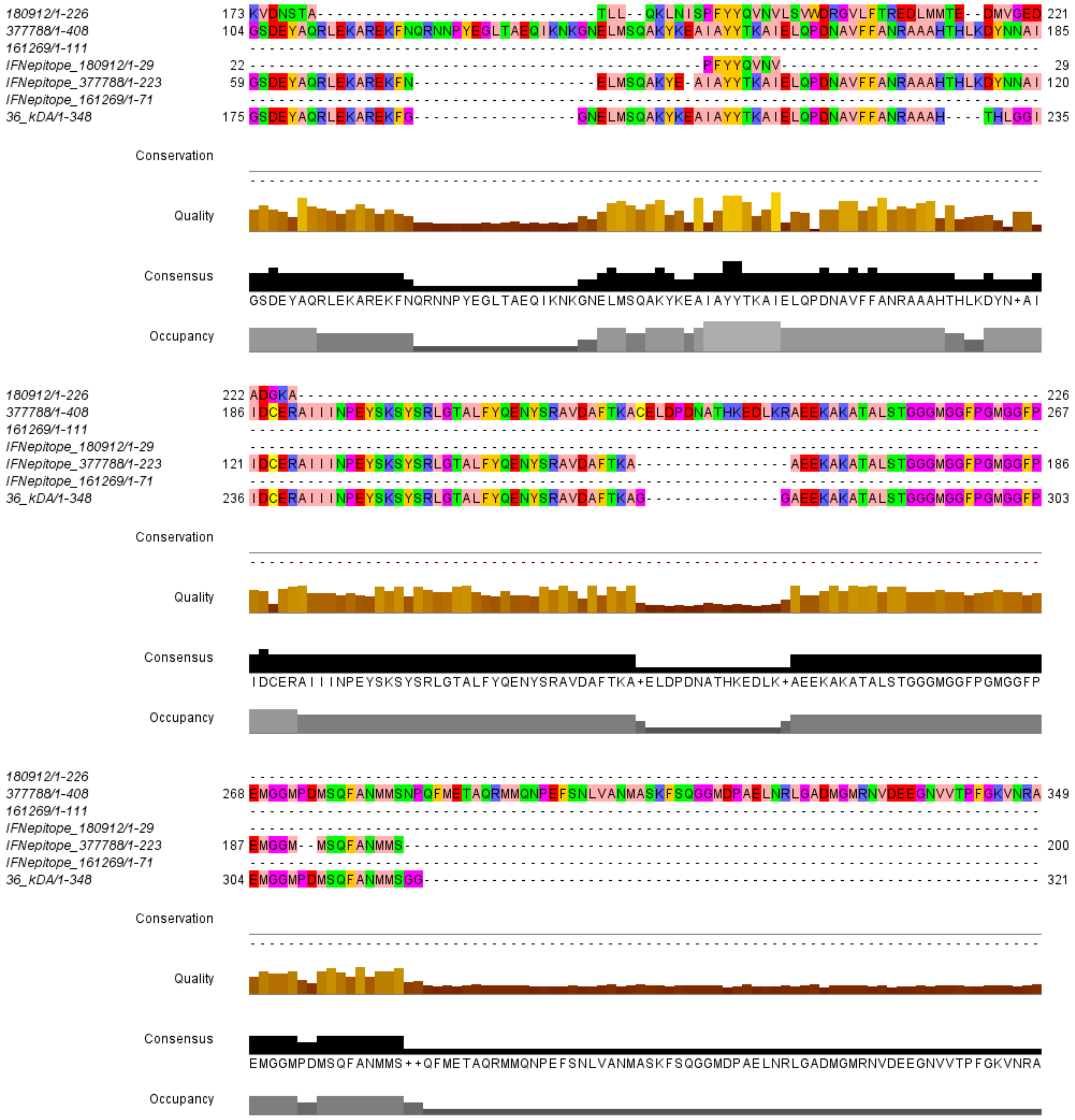
180912/1-226
377788/1-408
161269/1-111
IFNepitope_180912/1-29
IFNepitope_377788/1-223
IFNepitope_161269/1-71
36_kDA/1-348
Conservation
Quality
Consensus
Occupancy

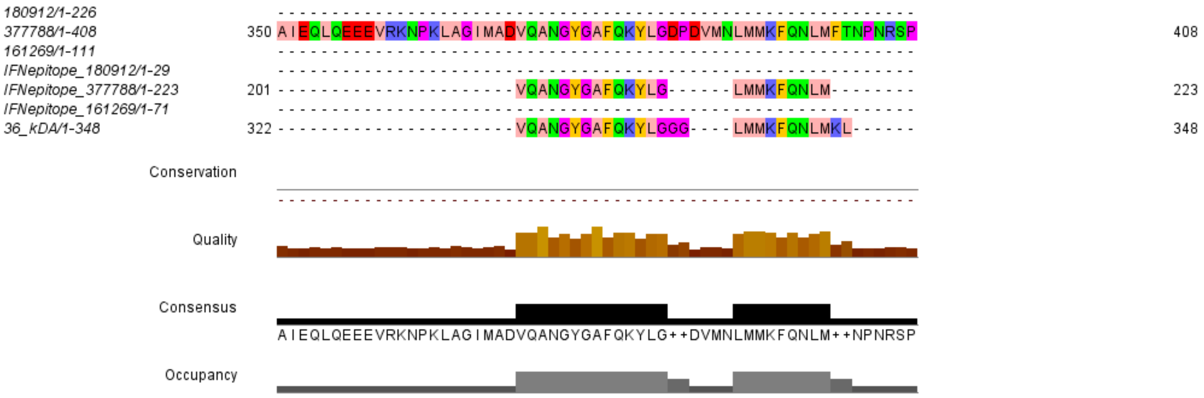

Supplementary Figure 5. Selection of epitopes for designing of construct 2 (36 kDa). Protein sequence alignment of 3 genes (A2/1, B4/1 & F2/1) with epitope prediction by IFNepitope for each gene, followed by selected sequences for multiepitope 36 kDa protein. Sequence alignment by Clustal Omega (EMBL-EBI).

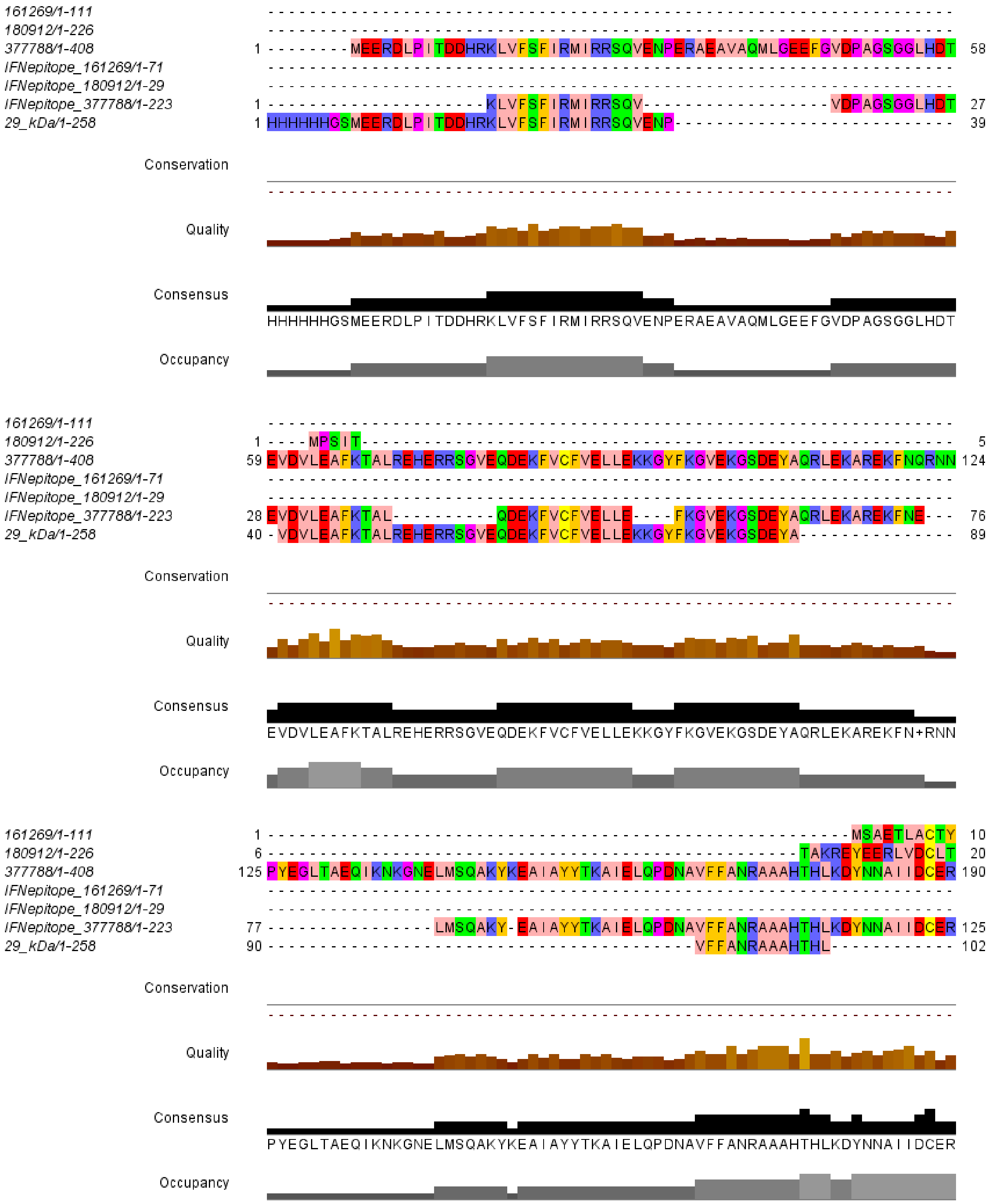
161269/1-111
180912/1-226
377788/1-408
IFNepitope_161269/1-71
IFNepitope_180912/1-29
IFNepitope_377788/1-223
29_kDa/1-258
Conservation
Quality
Consensus
HHHHHHGSMEERDLPITDDHRKLVFSFIRMIRRSQVENPERAEAVAQMLGEEFGVDPAGSGGLHDT
Occupancy
161269/1-111
180912/1-226
377788/1-408
IFNepitope_161269/1-71
IFNepitope_180912/1-29
IFNepitope_377788/1-223
29_kDa/1-258
Conservation
Quality
Consensus
EVDVLEAFKTALREHERRSGVEQDEKFVCFVELLEKKGYFKGVEKGSDEYAQRLEKAREKFN+RNN
Occupancy
161269/1-111
180912/1-226
377788/1-408
IFNepitope_161269/1-71
IFNepitope_180912/1-29
IFNepitope_377788/1-223
29_kDa/1-258
Conservation
Quality
Consensus
PYEGLTAEQIKNKGNELMSQAKYKEAIAYYTKAIELQPDNAVFFANRAAAHTHLKDYNNAIIDCER
Occupancy

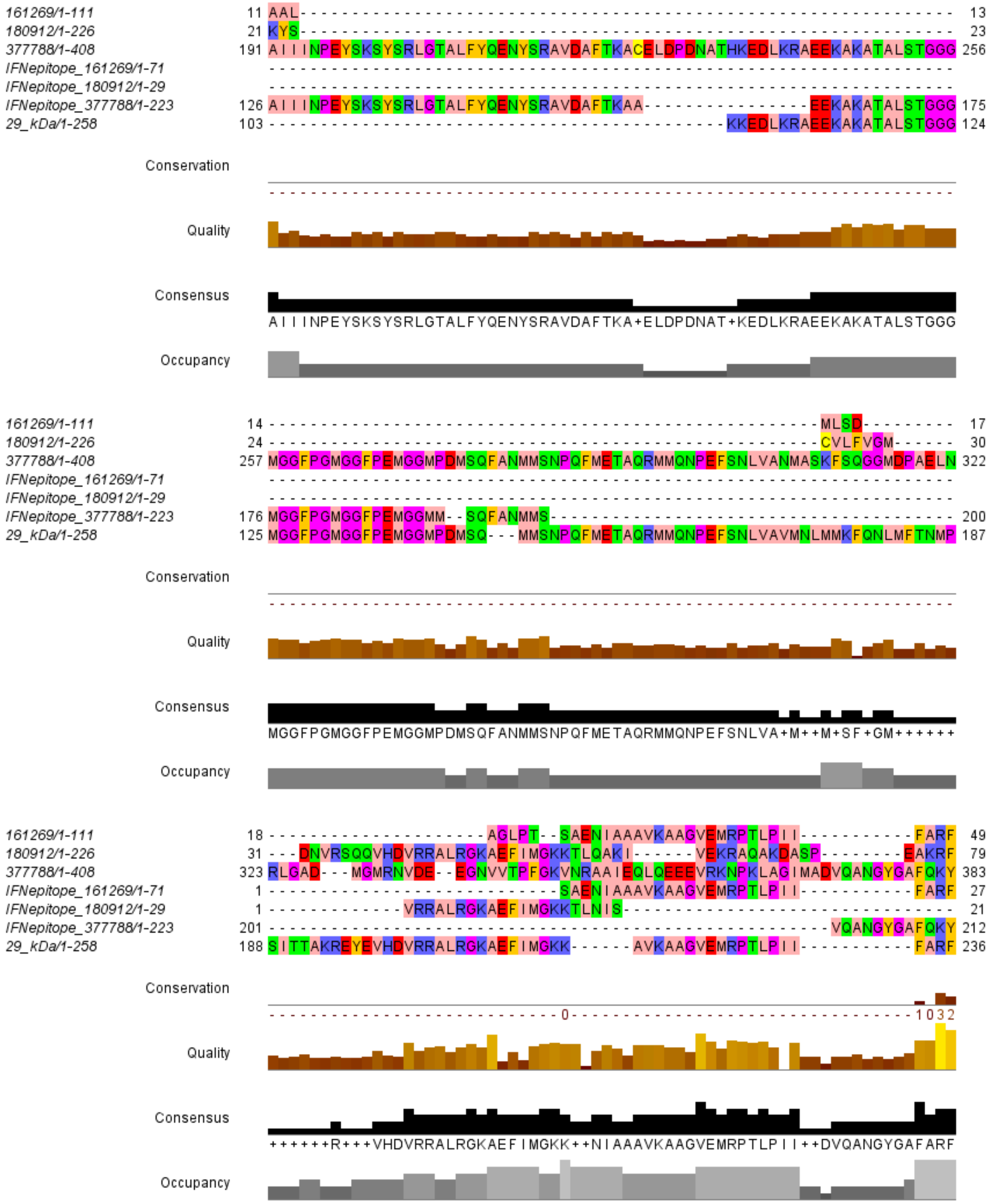
161269/1-111 11 AAL 13
180912/1-226 21 KYS 23
377788/1-408 191 AIIINPEYSKSYSRLGTALFYQENYSRAVDAFTKACELDPDNATHKEDLKRAEEKAKATALSTGGG 256
IFNepitope_161269/1-71
IFNepitope_180912/1-29
IFNepitope_377788/1-223 126 AIIINPEYSKSYSRLGTALFYQENYSRAVDAFTKAA EEKAKATALSTGGG 175
29_kDa/1-258 103 KKEDLKRAEEKAKATALSTGGG 124
Conservation
Quality
Consensus
AIIINPEYSKSYSRLGTALFYQENYSRAVDAFTKA+ELDPDNAT+KEDLKRAEEKAKATALSTGGG
Occupancy
161269/1-111 14 MLSD 17
180912/1-226 24 CVLFVGM 30
377788/1-408 257 MGGFPGMGGFPEMGGMPDMSQFANMMSNPQFMETAQRMMQNPEFSNLVANMASKFSQGGMDPAELN 322
IFNepitope_161269/1-71
IFNepitope_180912/1-29
IFNepitope_377788/1-223 176 MGGFPGMGGFPEMGGMM--SQFANMMS 200
29_kDa/1-258 125 MGGFPGMGGFPEMGGMPDMSQ---MMSNPQFMETAQRMMQNPEFSNLVAVMNLMMKFQNLMFTNMP 187
Conservation
Quality
Consensus
MGGFPGMGGFPEMGGMPDMSQFANMMSNPQFMETAQRMMQNPEFSNLVA+M++M+SF+GM++++++
Occupancy
161269/1-111 18 AGLPT--SAENIAAAVKAAGVEMRPTLPII FARF 49
180912/1-226 31 DNVRSQQVHDVRRALRGKAEFIMGKKTLQAKI VEKRAQAKDASP EAKRF 79
377788/1-408 323 RLGAD---MGMRNVDE--EGNVVTPFGKVNRAAIEQLQEEEVRKNPKLAGIMADVQANGYGAFQKY 383
IFNepitope_161269/1-71 1 SAENIAAAVKAAGVEMRPTLPII FARF 27
IFNepitope_180912/1-29 1 VRRALRGKAEFIMGKKTLNIS 21
IFNepitope_377788/1-223 201 VQANGYGAFQKY 212
29_kDa/1-258 188 SITTAKREYEVHDVRRALRGKAEFIMGKK AVKAAGVEMRPTLPII FARF 236
Conservation
0
1032
Quality
Consensus
++++++R+++VHDVRRALRGKAEFIMGKK++NIAAAVKAAGVEMRPTLPII++DVQANGYGAFARF
Occupancy

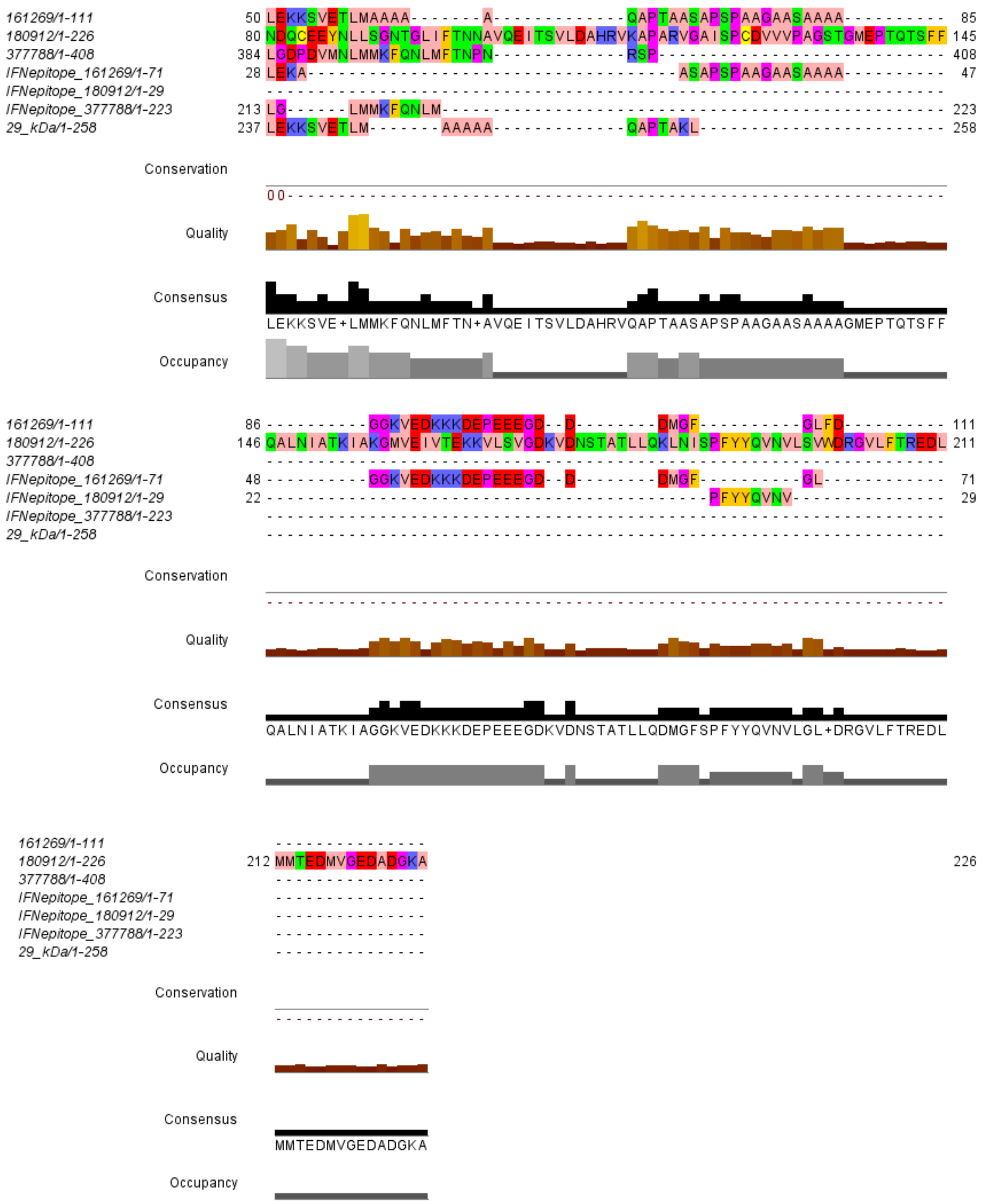

Supplementary Figure 6. Selection of epitopes for designing of construct 3 (29 kDa). Protein sequence alignment of 3 genes (A2/1, B4/1 & F2/1) with epitope prediction by IFNepitope for each gene, followed by selected sequences for multiepitope 29 kDa protein. Sequence alignment by Clustal Omega (EMBL-EBI).

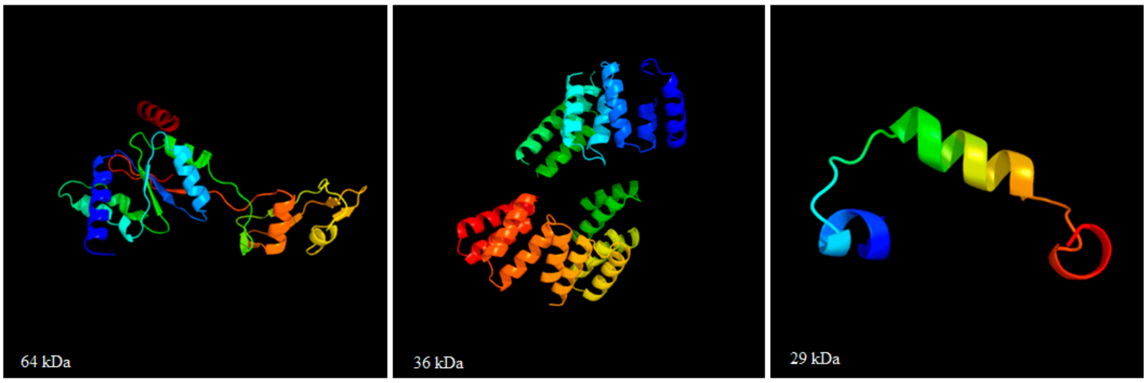

Supplementary Figure 7. 64 kDa, 36 kDa & 29 kDa protein structure. Structure prediction by Phyre2(Protein Homology/analogY Recognition Engine V 2.0).

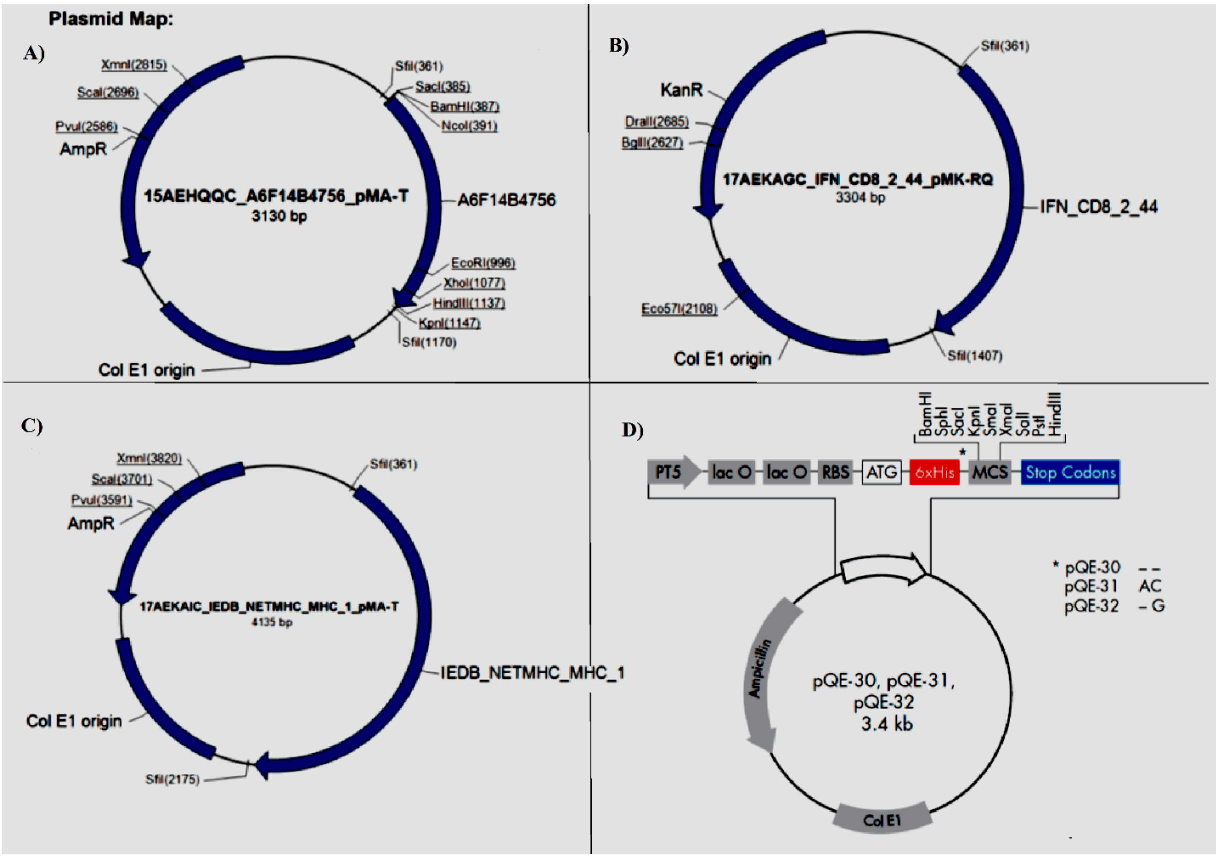

Supplementary Figure 8. A) Plasmid vector Map for 756 bp insert in pMA-T, total size 3130 bp, **B)** Plasmid vector Map for 1026 bp insert in pMK-RQ, total size 3304 bp, **C)** Plasmid vector Map for 1761 bp insert in pMA-T, total size 4135 bp & **D)** Plasmid vector Map for pQE30 (Qiagen), 3461 bp.

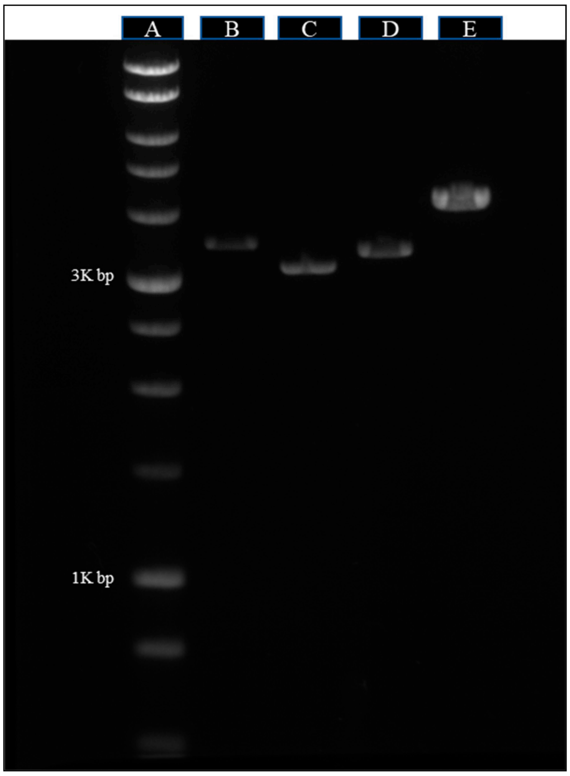

Supplementary Figure 9. Linear Plasmid separation on 1.5% Agarose gel: Lane A Ladder 1k bp, Lane B pQE30 3461 bp, Lane C pMA-T 3130 bp, Lane D pMK-RQ 3304 bp, & Lane E pMA-T 4135 bp. Ladder 1k bp, Cat. G5711 (Promega USA)

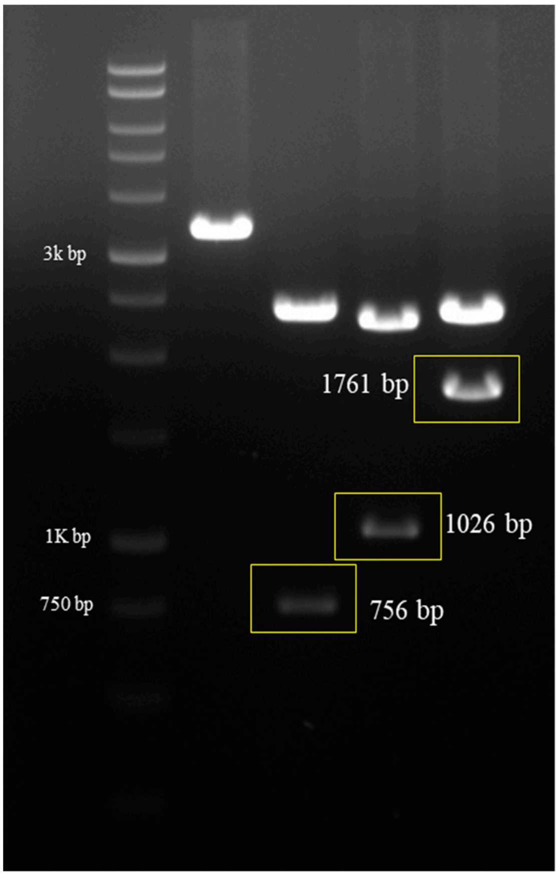

Supplementary Figure 10. Double digestion of plasmid, product separation on 1.5% Agarose gel: Lane A- Ladder 1k bp, Lane B- pQE30(3461), Lane C- pMA-T (2374+756), Lane D- pMk-RQ (2278+1026), lane E pMA-T (2374+1761). Ladder 1k bp, Cat. G5711 (Promega USA)

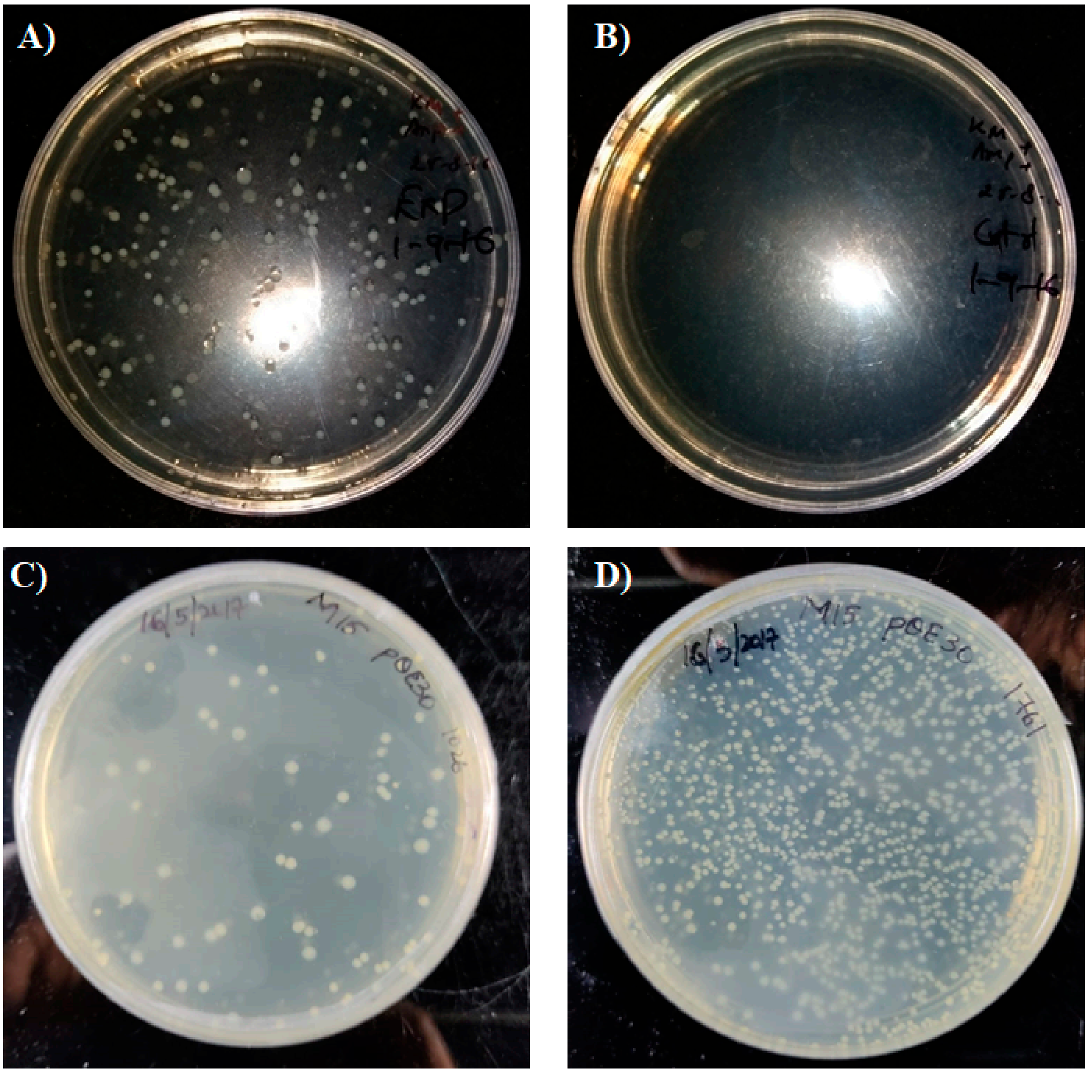

Supplementary Figure 11. LB agar plates with overnight culture of transformed *E. coli(*M15). **A)** transformation with vector plus 756 bp inserts, **B)** empty vector (w/o insert), **C)** transformation with vector plus 1036 bp insert & **D)** transformation with vector with 1761 bp insert.

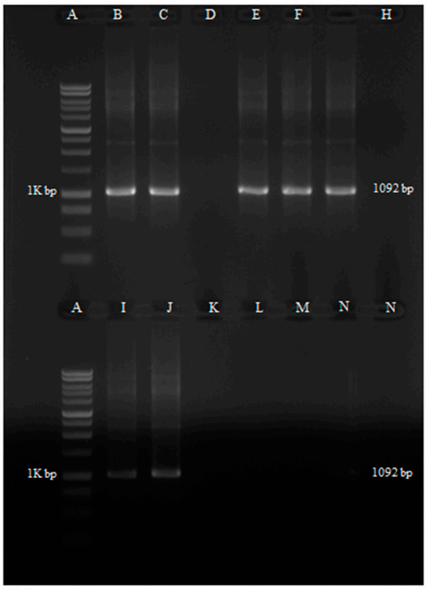

A)

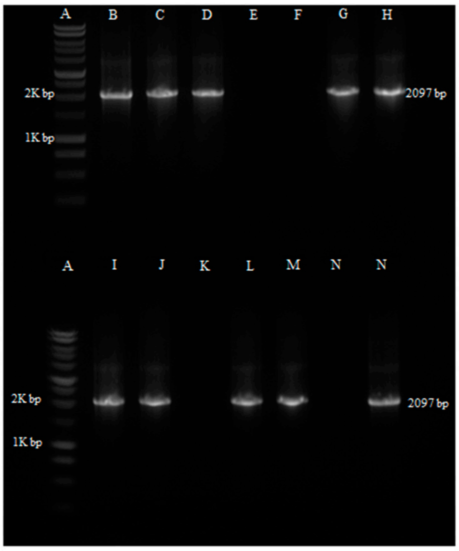

B)

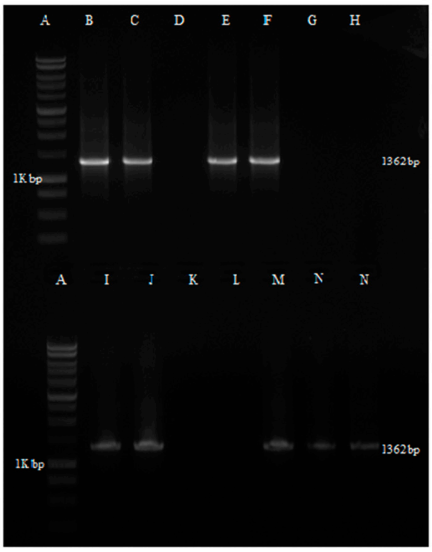

C)

Supplementary Figure 12. A) 1 % Agarose gel separation of Colony PCR products, for 756 bp insert confirmation, of expected band size 336+ 756= 1092 bp. Lane A 1kb Ladder, Lane B to N bacteria colony. **B)** 1 % Agarose gel separation of Colony PCR products, for expected band size of 2097 bp (336+ 1761). **C)** 1 % Agarose gel separation of Colony PCR products, for expected band size 1362 bp (336+1026) on right. Lane A 1kb Ladder, Lane B to N, bacteria colony. Ladder 1k bp, Cat. G5711 (Promega USA)

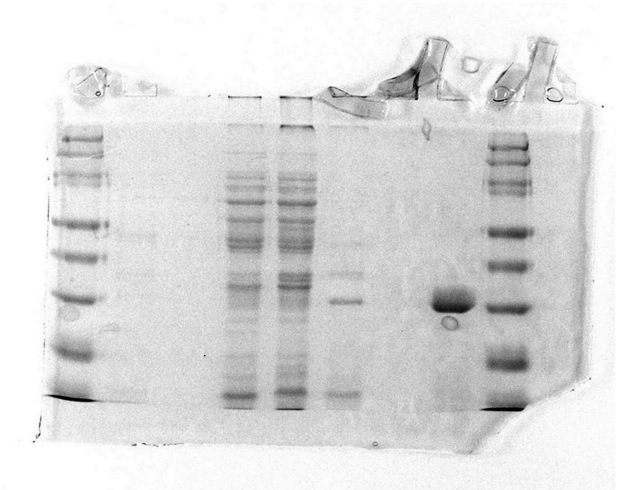

1)

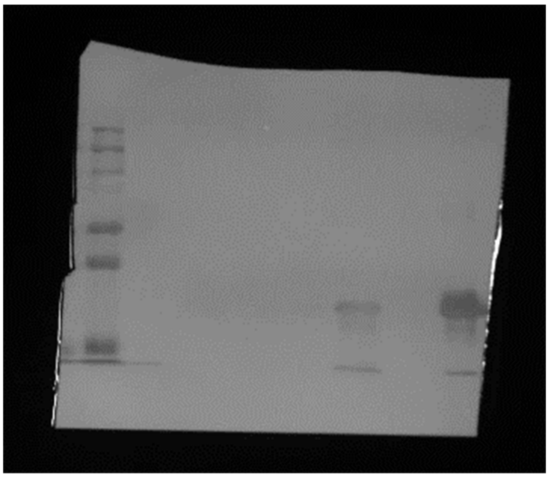

2)

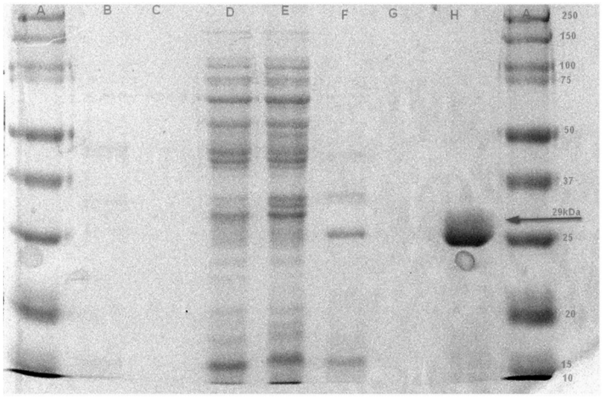

12% SDS-PAGE, Coomassie Blue Stained

3)

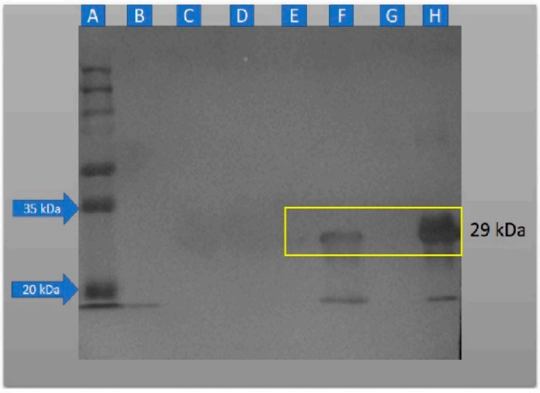

Western BLot for Poly-HIS antibody, Substrate System 3,3′,5,5′-Tetramethylbenzidine (TMB)

4)

Supplementary Figure 13 A. 12% SDS-PAGE, Coomassie stain and Western Blot using Poly-HIS antibody, for different steps of protein formation and isolation from inclusion bodies of *E coli*(M15), using 0.1 mM IPTG overnight at 37°C**.** Lane A: Ladder 250 kDa (BioRad 1610374) B: LB broth after IPTG induction C: Empty Lane, D: Bacterial lysate after 10,000 g spin E: Resuspension buffer discard, F: Deoxycholic acid wash discard, G: Final wash discard, and H: Resuspended Protein 29 kDa pellet in ddH_2O. Western blot, poly-HIS antibody with substrate system 3,3′,5,5′-Tetramethylbenzidine (TMB). 1) & 2). Uncropped images. 3) & 4). Cropped Images.

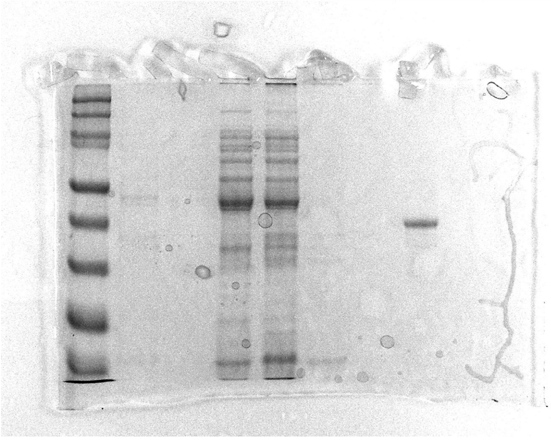

1)

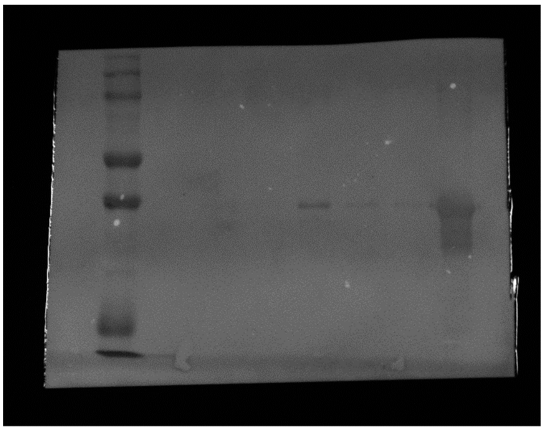

2)

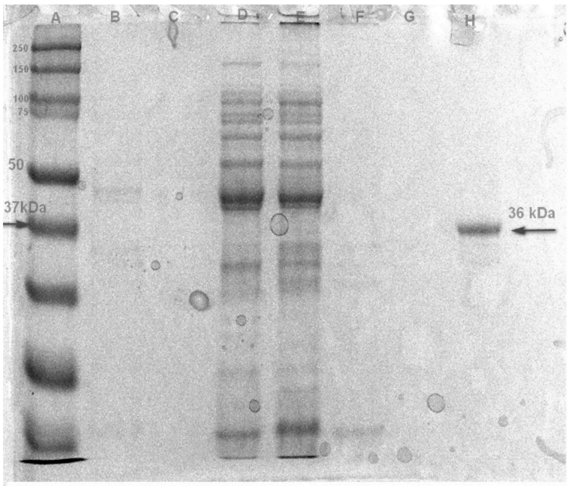

12 % SDS-PAGE Coomassie blue Stained

3)

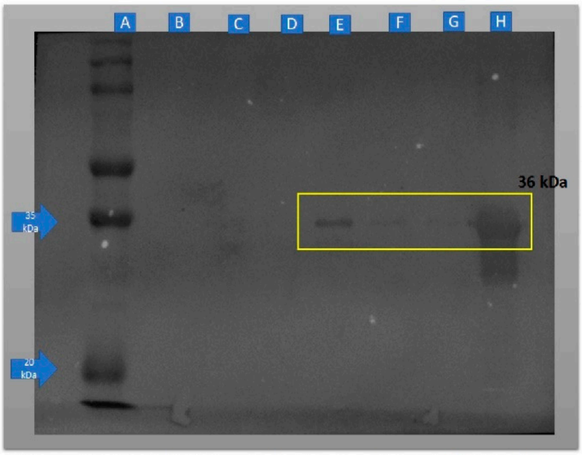

Western Blot for Poly-HIS antibody, Substrate System TMB (3,3′,5,5′-Tetramethylbenzidine)

4)

Supplementary Figure 13 B. 12% SDS-PAGE, Coomassie stain and Western Blot using Poly-HIS antibody, for different steps of protein formation and isolation from inclusion bodies of *E coli*(M15), using 0.1 mM IPTG overnight at 37°C**.** Lane A: Ladder 250 kDa (BioRad 1610374) B: LB broth after IPTG induction C: Empty Lane, D: Bacterial lysate after 10,000 g spin E: Resuspension buffer discard, F: Deoxycholic acid wash discard, G: Final wash discard, and H: Resuspended Protein 36 kDa pellet in ddH_2O. Western blot, poly-HIS antibody with substrate system 3,3′,5,5′-Tetramethylbenzidine (TMB). 1) & 2). Uncropped images. 3) & 4). Cropped Images.

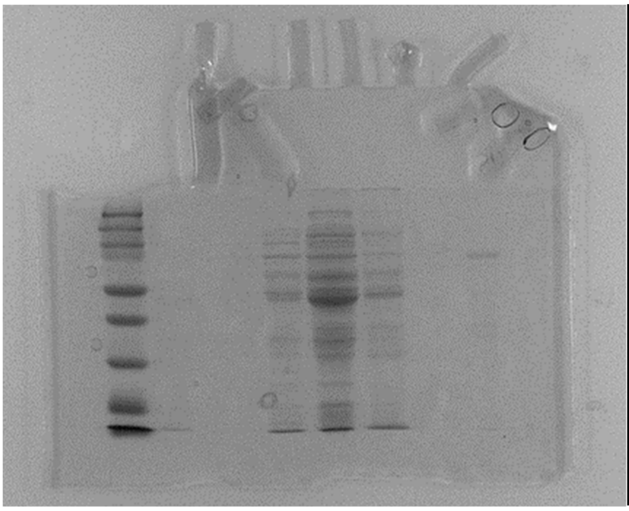

1)

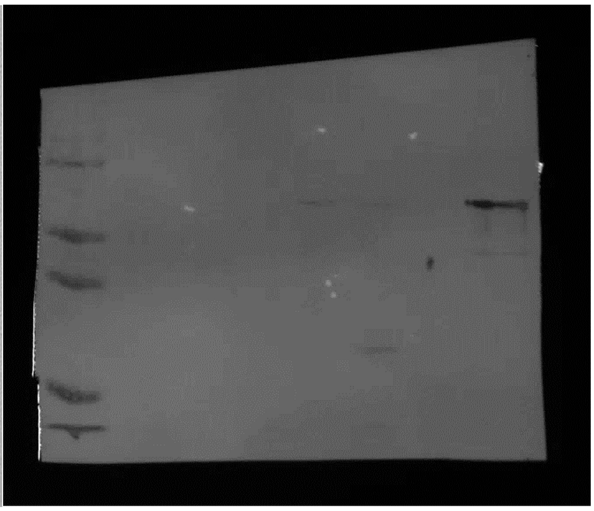

2)

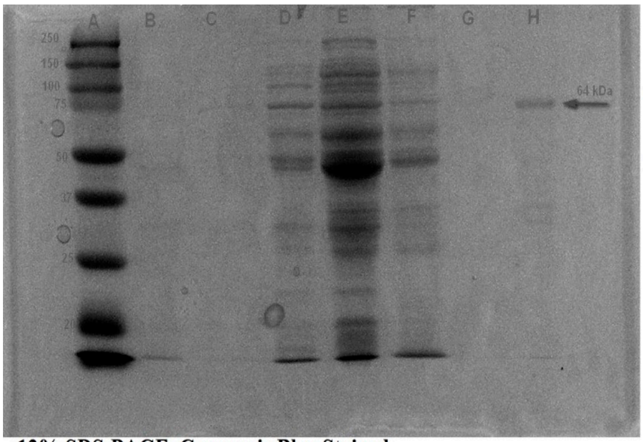

12% SDS-PAGE, Coomassie Blue Stained

3)

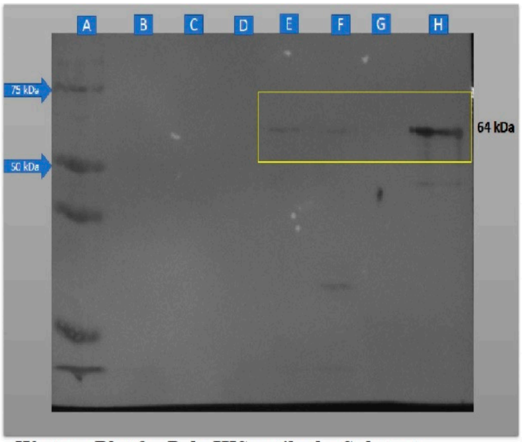

Western Blot for Poly-HIS antibody, Substrate System3,3′,5,5′-Tetramethylbenzidine (TMB)

4)

Supplementary Figure 13 C. 12% SDS-PAGE, Coomassie stain for different steps of protein formation and isolation from inclusion bodies of *E. coli*(M15), using 0.1 mM IPTG overnight at 37°C. Lane A: Ladder 250 kDa (BioRad 1610374) B: LB broth after IPTG induction C: Empty Lane, D: Bacterial lysate after 10,000 g spin E: Resuspension buffer discard, F: Deoxycholic acid wash discard, G: Final wash discard, and H: Resuspended Protein 64 kDa pellet in ddH$_2$O. Western blot, poly-HIS antibody with substrate system 3,3′,5,5′-Tetramethylbenzidine (TMB). 1) & 2). Uncropped images. 3) & 4). Cropped Images.

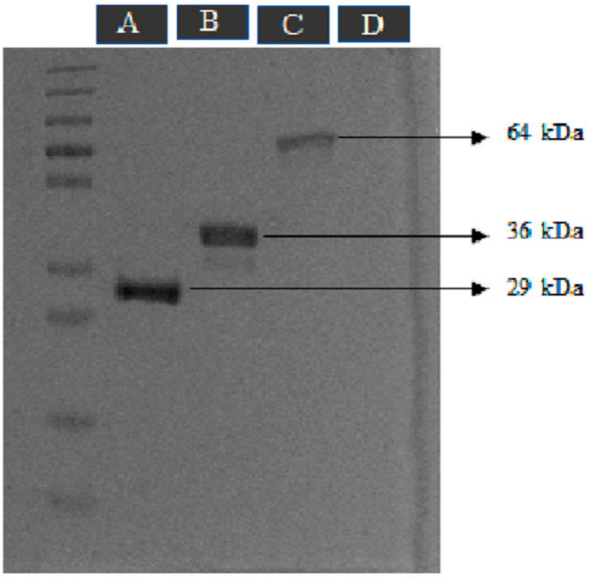

Supplementary Figure 13 D. Protein separation on 10% SDS-PAGE Lane A: protein ruler (Page Ruler™ Prestained, Cat. 26617, ThermoFisher, USA), lane B: 29 kDa, lane C: 36 kDa & lane D: 64 kDa.

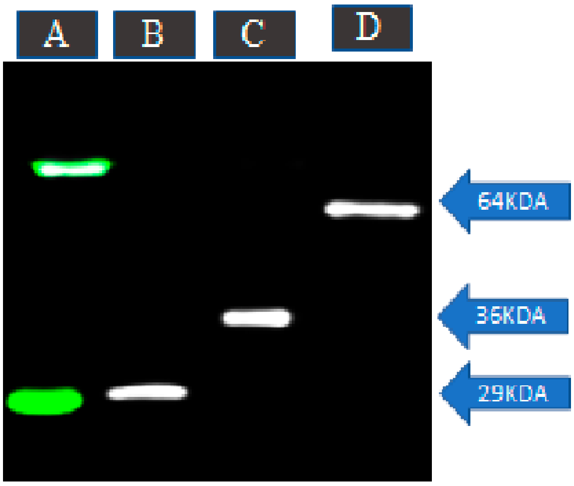

1)

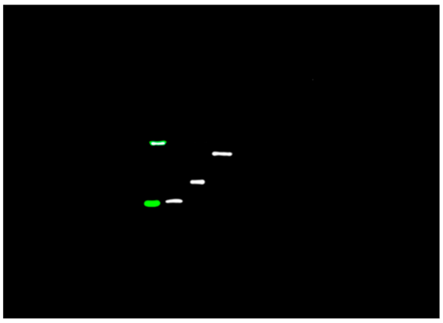

2)

Supplementary Figure 14. Western blot analysis of Recombinant Constructs based on Poly-His antibodies. Lane A Protein ruler (#1610374 Bio Rad Precision plus™ USA), lane B 29 kDa construct, lane C 36 kDa construct & lane D 64 kDa construct. Substrate system Clarity™ Western ECL Substrate (#1705060 BioRad). 1) Cropped Image, 2) Uncropped image.

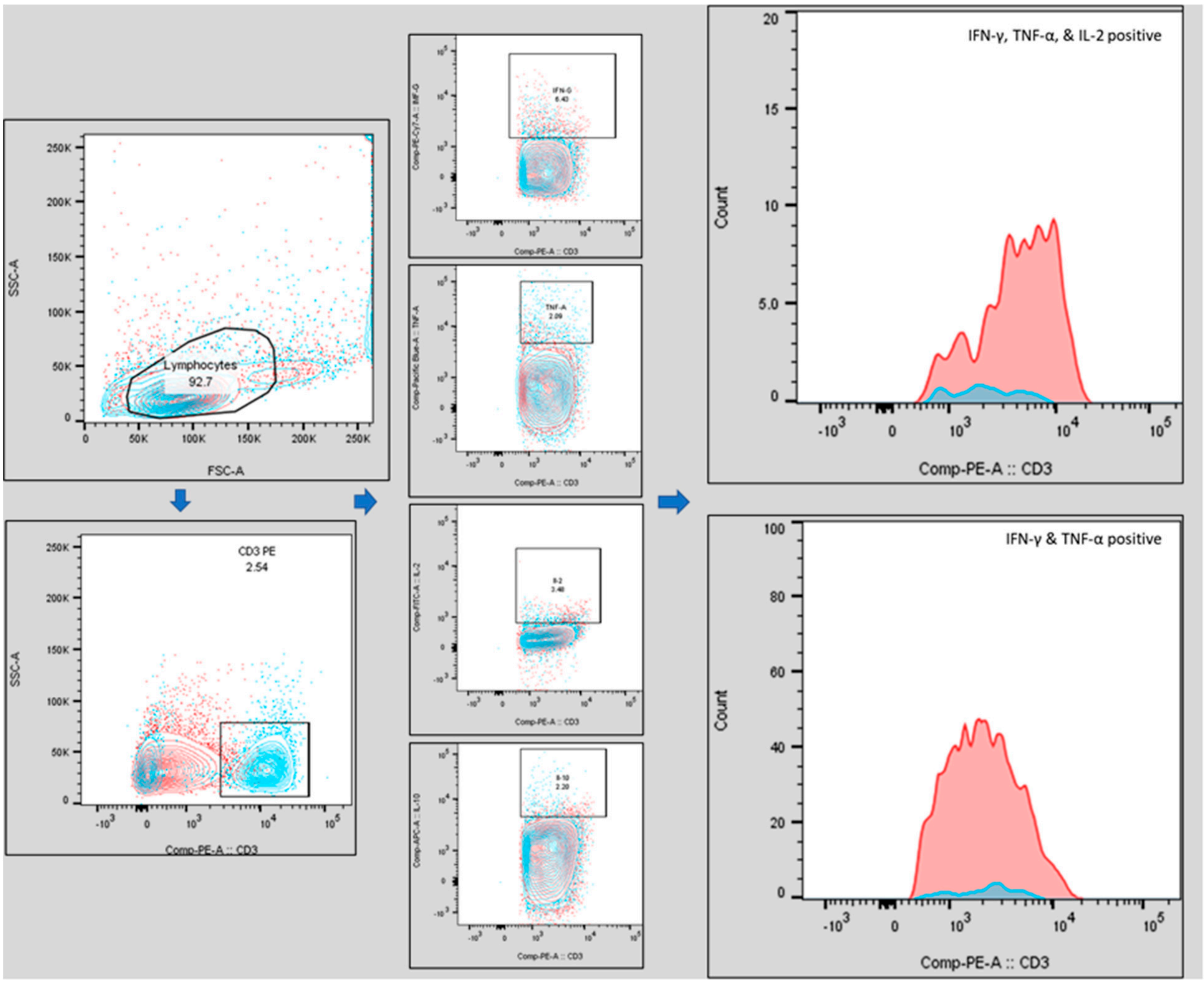

Supplementary Figure 15. Gating Strategy For identification of polyfunctional CD3$^+$ T cells. Blue: Unstimulated Stained cells, Red: Stimulated Stained cells. PBMC gated for Lymphocytes based on FSC and SSC. Lymphocytes gated for CD3$^+$ T cells based on PE-CD3 antibody. IFN-γ inducing cells gated on the basis of PE-cy-7, for TNF-α Pacific-Blue, for IL-2 FITC and for IL-10 APC. Negative gates set on the basis of stained unstimulated samples. Triple positive and dual positive cells identified on the basis of Boolean gating. Data analysis done using FlowJo™ V.10 (BD Bioscience US).

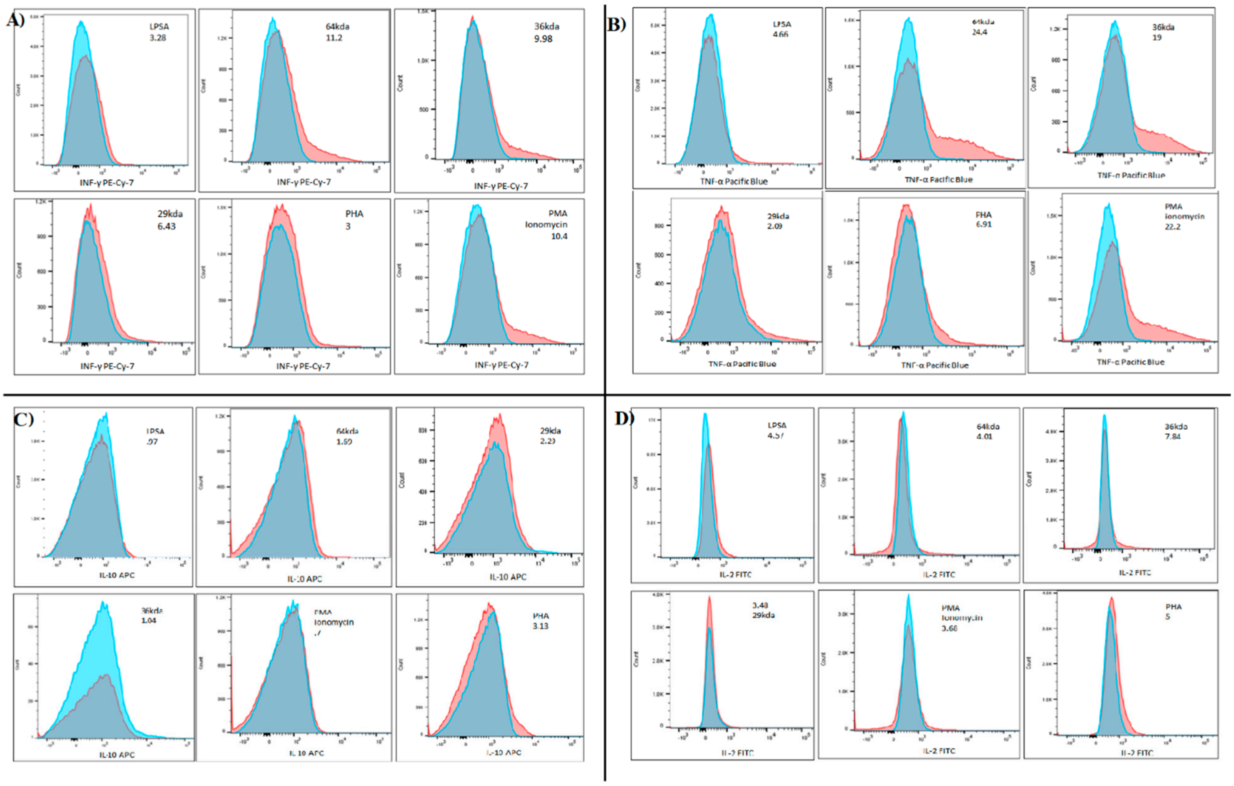

Supplementary Figure 16. Histogram analysis of IFN-γ, B: TNF-α, C: IL-2 & D: IL-10 secreting CD3$^+$ T cells. **A)** Frequency of IFN-γ secretory cells, **B)** Frequency of TNF-α secretory cells, **C)** Frequency of IL-2 secretory cells, & **D)** Frequency of IL-10 secretory cells. Red, stimulated stained cells. Blue, unstimulated stained cells. LPSA (Leishmania Promastigote Soluble Antigen), 64 kDa construct, 36 kDa construct, 29 kDa construct, Phorbol myristate acetate (PMA) 100ng/ml with Ionomycin (50ng/ml), Phytohemagglutinin (PHA) 10µg/ml.

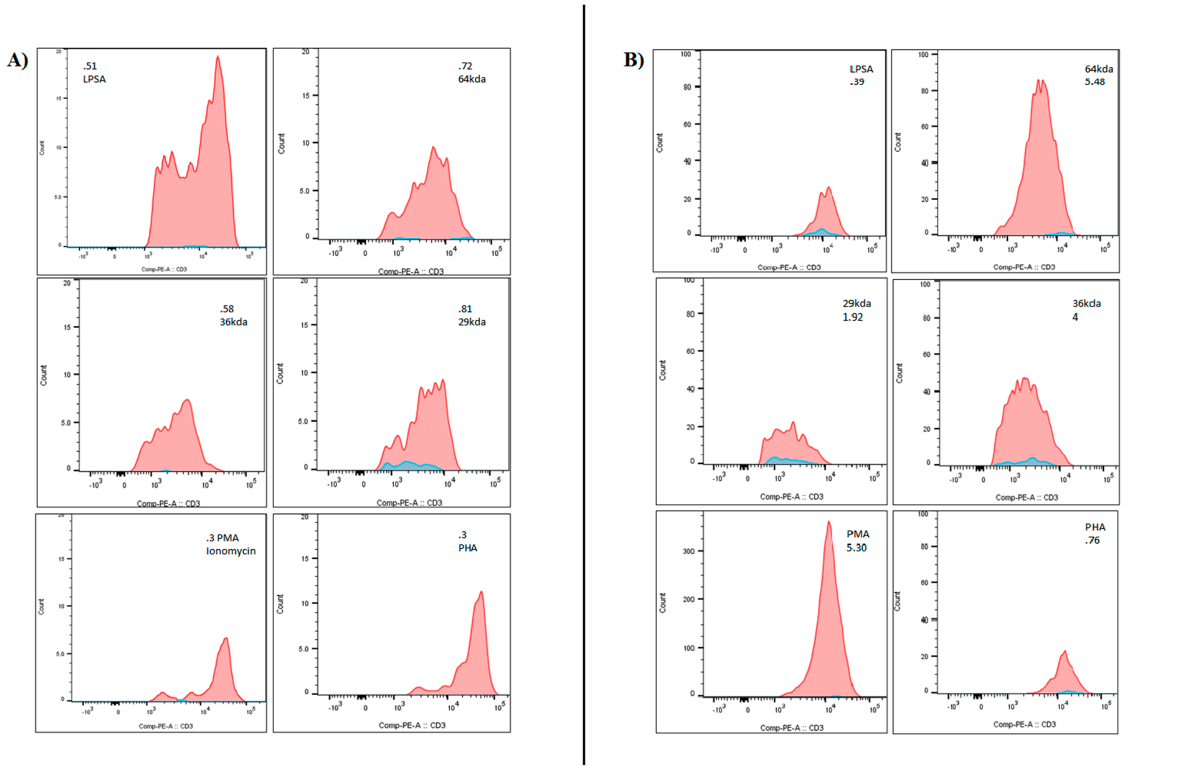

Supplementary Figure 17. Histogram analysis of **A)** simultaneous INF-γ, TNF-α, and IL-2 producing CD3^{+} T cells (Polyfunctional T cells) & **B)** IFN-γ and TNF-α producing double positive cells. Red, stimulated stained cells. Blue, unstimulated stained cells. Polyfunctional T cells percentage stimulation by, LPSA (*Leishmania* Promastigote Soluble Antigen), 64 kDa construct, 36 kDa construct, 29 kDa construct, Phorbol myristate acetate (PMA) 100ng/ml along with Ionomycin (50ng/ml), Phytohemagglutinin (PHA) 10µg/ml.

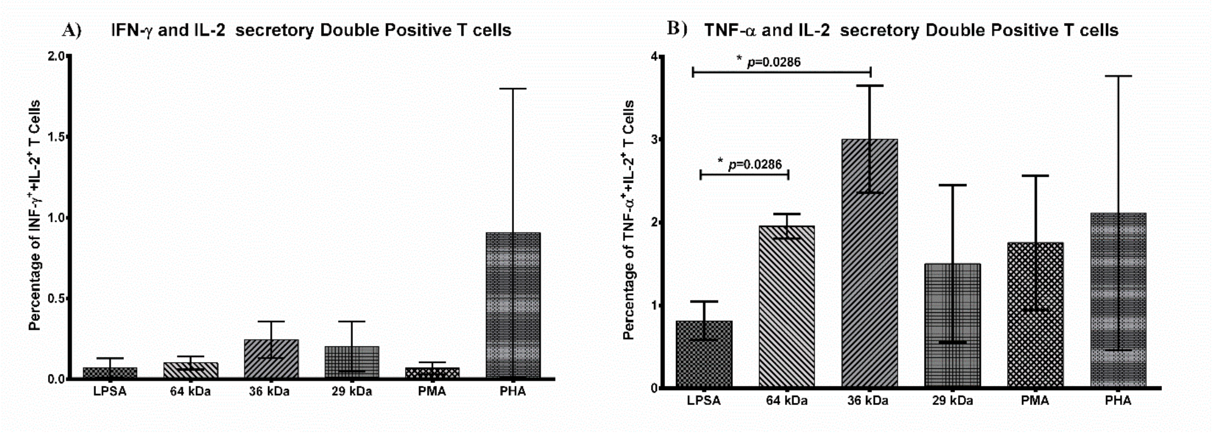

Supplementary Figure 18. Bar-graph analysis of double positive CD3+ T cells **A)** simultaneous INF-γ and IL-2 producing double positive cells $CD3^+$ T cells and **B)** TNF-α and IL-2 producing double positive cells $CD3^+$ T cells. Mean ±SD. Significance level determined by Student's t-test, *$p < 0.05$ compared to LPSA treated group, ns $p > 0.05$, * $p \leq 0.05$.

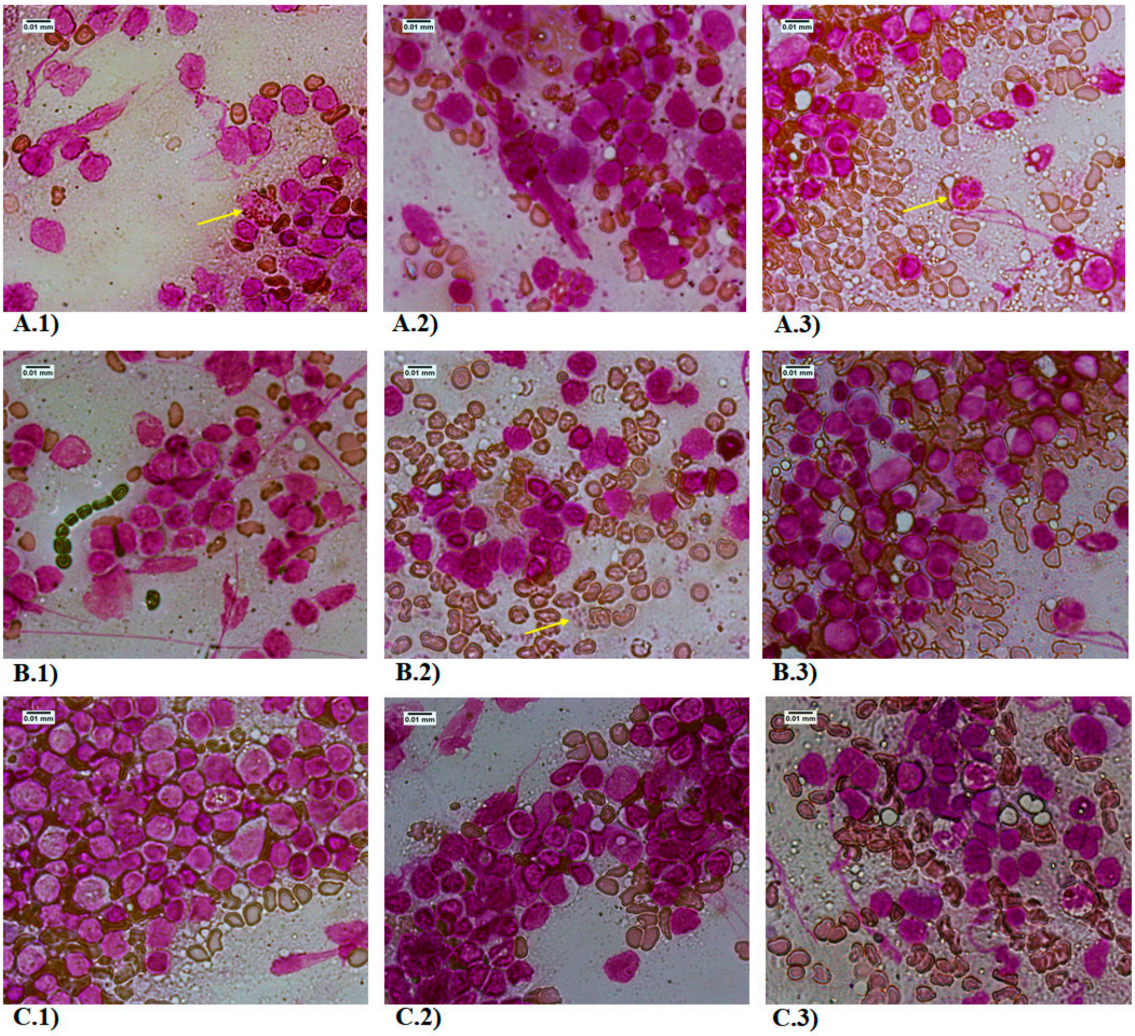

Supplementary Figure 19A. Giemsa-stained touch impressions of spleen from different animal groups. **A.1-3)** Unimmunized Infected animal, **B.1-3)** Complete Freund's adjuvant (CFA) only, & **C.1-3)** healthy animal. Arrows indicating the infected cells. *Magnification 10×100X* (oil immersion) (Olympus, Tokyo, Japan). **Supplementary Figure 19B.** Giemsa-stained touch impressions of spleen from different animal groups. **A.1-3)** 29 kDa immunized animal, **B.1-3)** 36 kDa immunized animal, & **C.1-3**) 64 kDa immunized animal. Arrows indicating the infected cells. *Magnification 10×100X* (oil immersion) (Olympus, Tokyo, Japan).

(A)

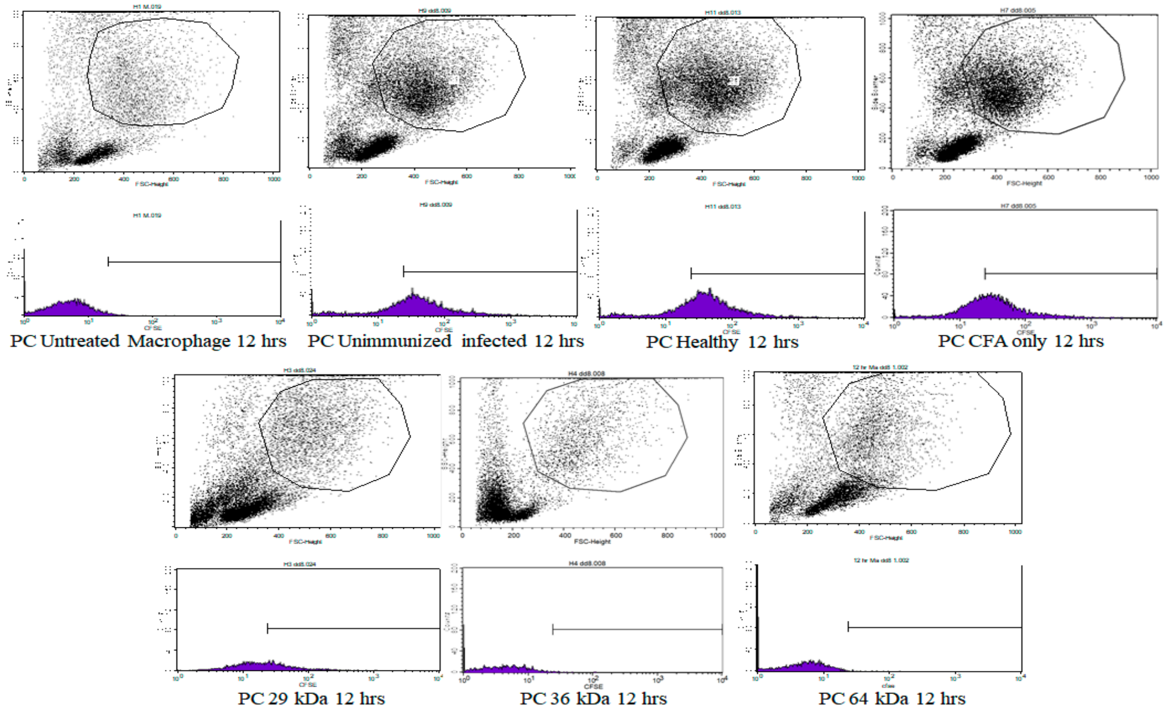
PC Untreated Macrophage 12 hrs
PC Unimmunized infected 12 hrs
PC Healthy 12 hrs
PC CFA only 12 hrs
PC 29 kDa 12 hrs
PC 36 kDa 12 hrs
PC 64 kDa 12 hrs

(B)

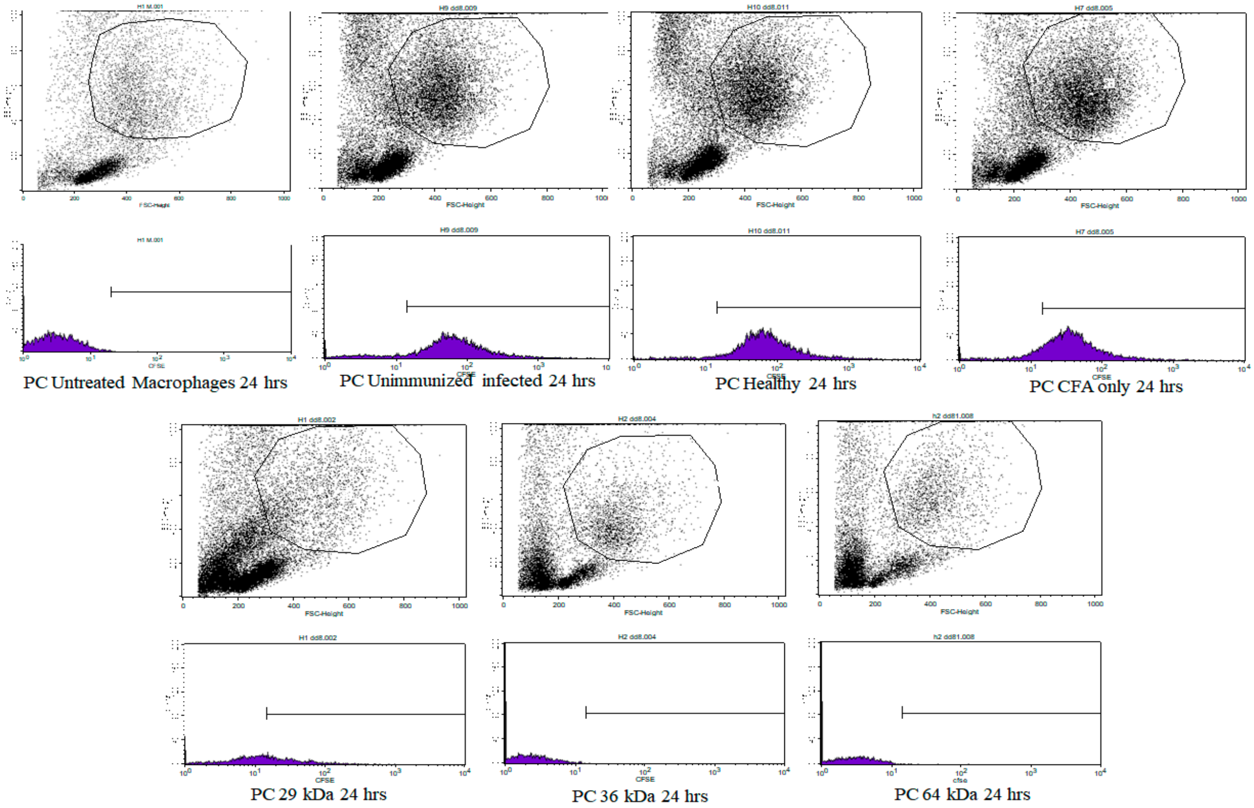
PC Untreated Macrophages 24 hrs
PC Unimmunized infected 24 hrs
PC Healthy 24 hrs
PC CFA only 24 hrs
PC 29 kDa 24 hrs
PC 36 kDa 24 hrs
PC 64 kDa 24 hrs

(C)

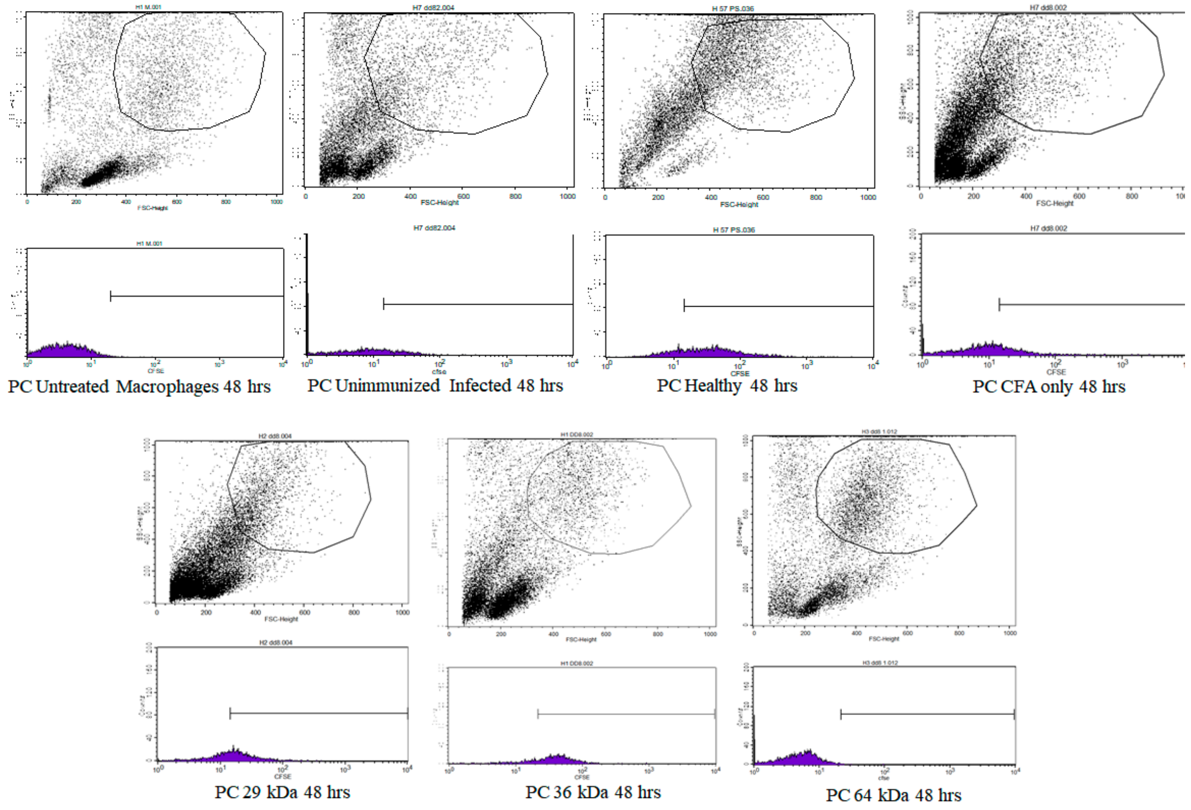
PC Untreated Macrophages 48 hrs
PC Unimmunized Infected 48 hrs
PC Healthy 48 hrs
PC CFA only 48 hrs
PC 29 kDa 48 hrs
PC 36 kDa 48 hrs
PC 64 kDa 48 hrs

(D)

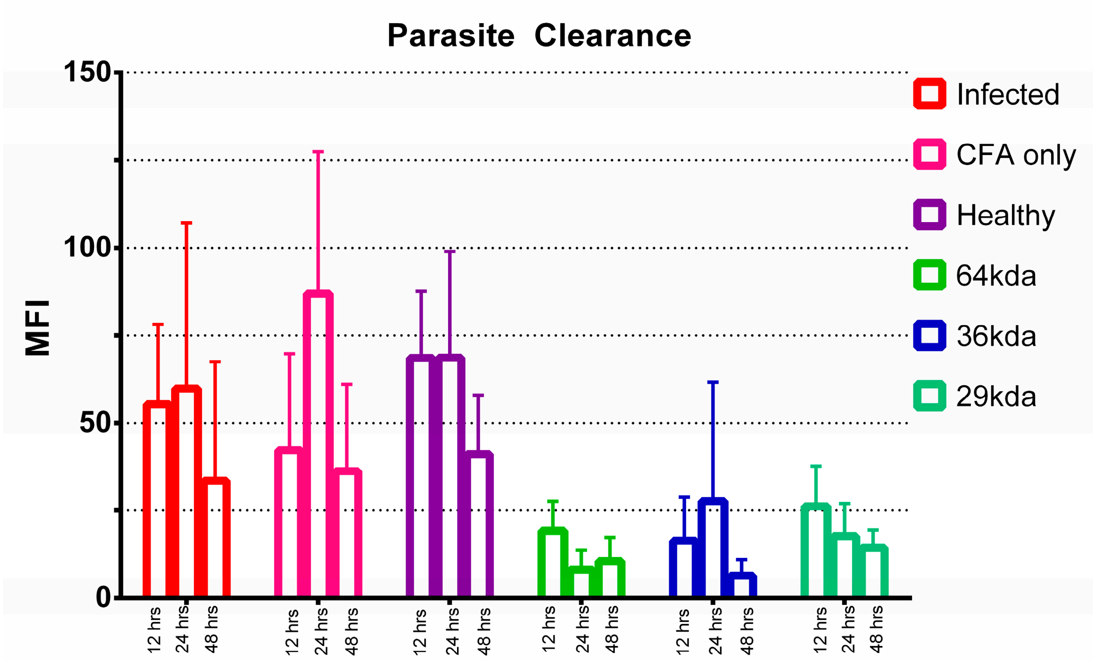
Parasite Clearance
MFI
150
100
50
0
Infected
CFA only
Healthy
64kda
36kda
29kda
12 hrs
24 hrs
48 hrs

Supplementary Figure 20. Representative image for flowcytometry analysis of CFSE signal from adherent peritoneal cells infected with CFSE stained *Leishmania donovani*. Scatter Plot based gating strategy for Polymorphonuclear cells based on FSC and SSC pattern. Histogram for CFSE signal from adherent peritoneal cells from, unimmunized animal, healthy animal, CFA only animal, 29 kDa immunized animal, 36 kDa immunized animal, and 64 kDa immunized animal, **20A)** Parasite clearance level at 12 hrs, **20 B)** Parasite Clearance level at 24 hrs, 20 C) Parasite Clearance level at 48 hrs. **20 D)** Parasite clearance at different time for all groups, Mean values ±SD.

(A)

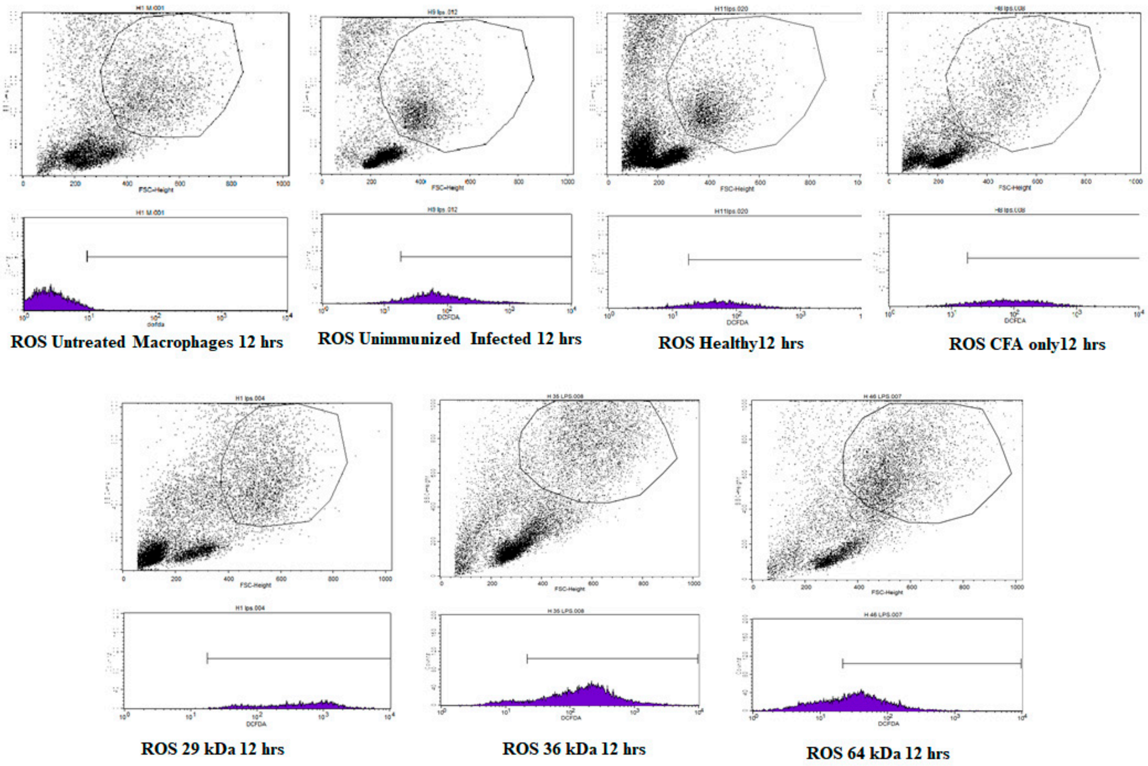

(B)

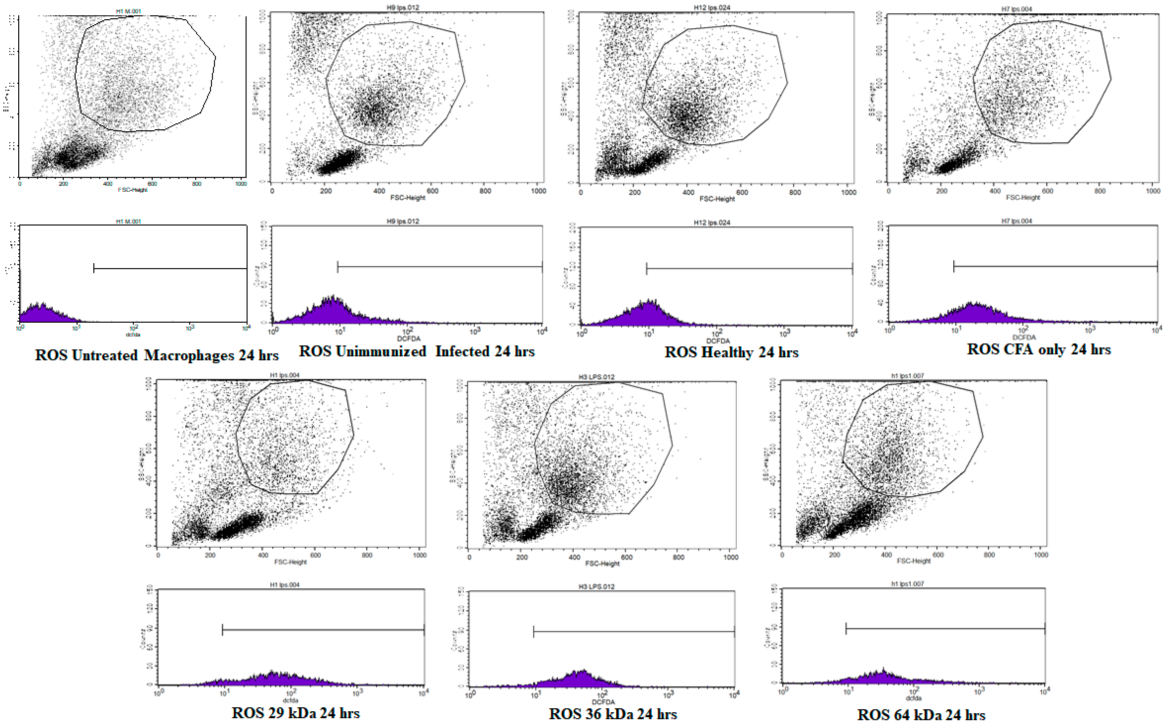

Supplementary Figure 21. Representative image for flowcytometry analysis for 2,7-dichlorofluorecein levels from peritoneal adherent cells of different animal groups. Scatter Plot based gating strategy for Polymorphonuclear cells based on FSC and SSC pattern. Histogram of 2',7'-dichlorofluorescein (DCF) intensity and number of cells, unimmunized infected animal, healthy animal, CFA alone animal, 29 kDa immunized animal, 36 kDa immunized animal, and 64 kDa immunized animal, **21A)** ROS production level at 12 hrs, and **21 B)** ROS production level at 24 hrs.

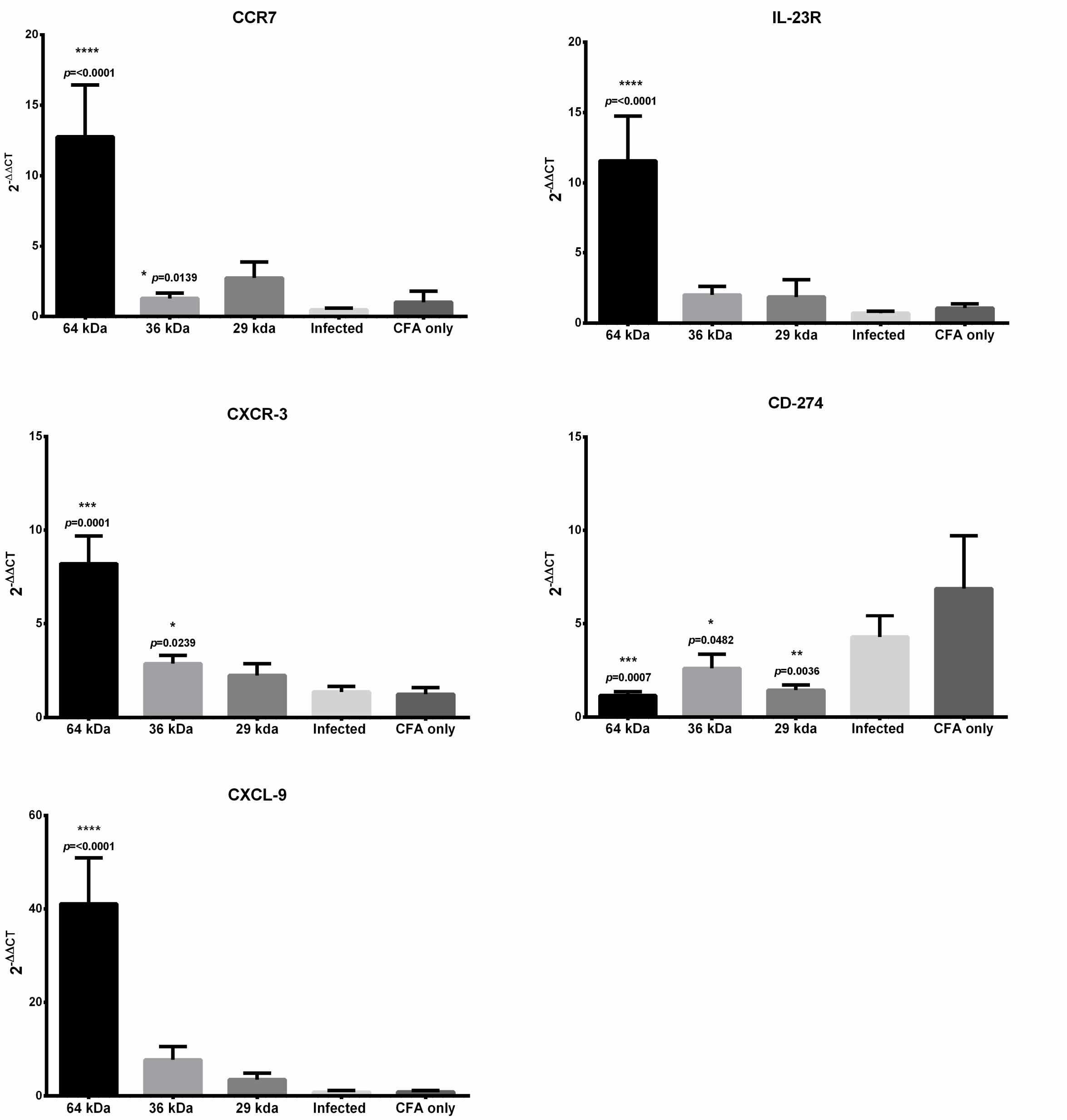

Supplementary Figure 22. Relative fold change in gene expression levels of CCR7, IL-23r, CXCR-3, CD-274 & CXCL-9 from immunized and unimmunized animals. Statistics: Student t-test. Comparison between test group and infected. Level of significances ns $p>0.05$, *$p\leq0.05$, **$p\leq0.01$, ***$p\leq0.001$, ****$p\leq0.0001$.

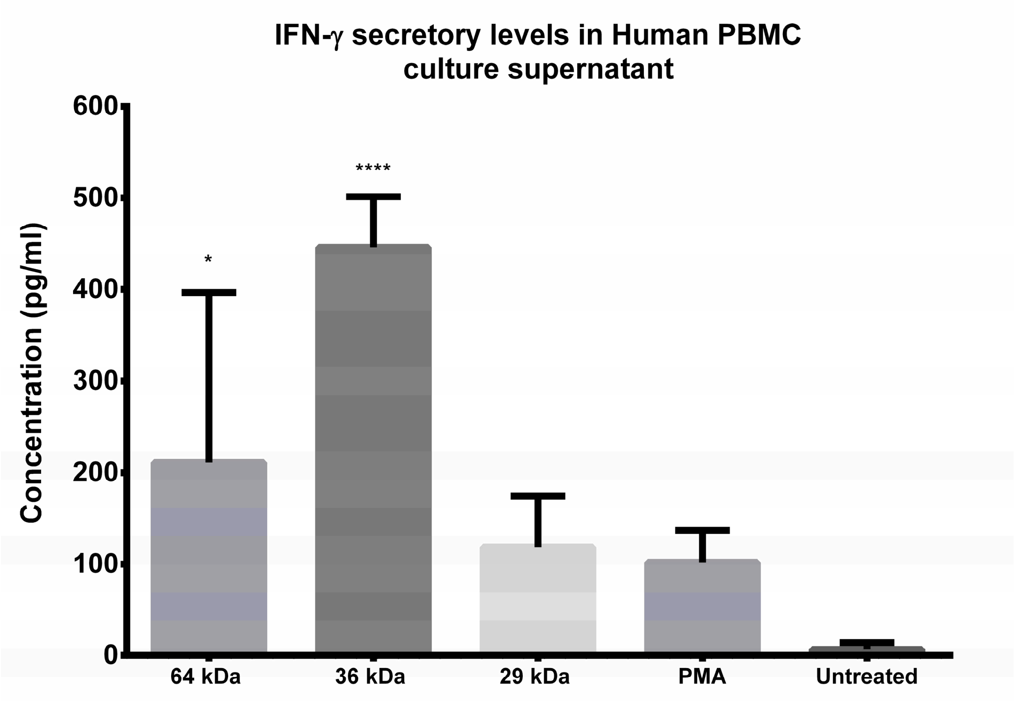

Supplementary Figure 23. IFN-γ levels as measured from the culture supernatant of human PBMC's stimulated with vaccine candidate proteins. Detection method ELISA. Difference statistically significant as determined by ANOVA ($p < 0.05$). (Level of significance, ns p>0.05, * $p \leq 0.05$, ****$p \leq 0.0001$).

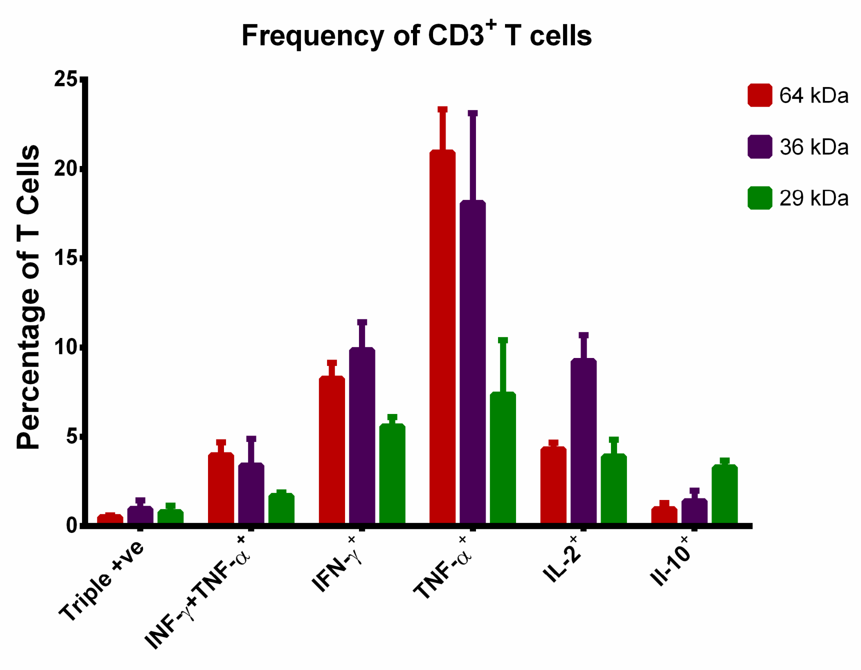

Supplementary Figure 24. Frequency of T cells positive for polyfunctionality and cytokine secretion when induced with the three proteins.
